# Diastereomeric Branched-Ester dBET1 Analogs Exhibit Conformation-Dependent Differences in Passive Membrane Permeability

**DOI:** 10.1021/acs.jmedchem.5c02791

**Published:** 2026-01-20

**Authors:** Mazin A. S. Abdelwahid, Eisuke Hayakawa, Keigo Hirai, Mayumi Ishii, Kayoko Kanamitsu, Saori Yasuda, Fumiaki Ohtake, Shinichi Sato, Shusuke Tomoshige, Minoru Ishikawa

**Affiliations:** 1 Graduate School of Life Sciences, 13101 Tohoku University, 2-1-1 Katahira, Aoba-ku, Sendai 980-8577, Japan; 2 Graduate School of Pharmaceutical Sciences, The University of Tokyo, 7-3-1 Hongo, Bunkyo-ku, Tokyo 113-0033, Japan; 3 School of Pharmacy and Pharmaceutical Sciences, Hoshi University, 2-4-41 Ebara, Shinagawa-Ku, Tokyo 142-8501, Japan; 4 Institute for Advanced Life Sciences, Hoshi University, 2-4-41 Ebara, Shinagawa-Ku, Tokyo 142-8501, Japan; 5 Frontier Research Institute for Interdisciplinary Sciences, 13101 Tohoku University, 6-3 Aramaki aza-Aoba, Aoba-ku, Sendai 980-8577, Japan

## Abstract

Proteolysis-targeting chimeras (PROTACs) represent a promising therapeutic modality, but their clinical translation is often hindered by poor pharmacokinetic properties associated with their location in the “beyond Rule of 5” chemical space. Using the BRD4 degrader dBET1 as a model, this study explored a dual approach to improve the cellular permeability of PROTACs by combining amide-to-ester substitution with the strategic linker methylation to induce stereochemistry-driven conformational modulation. Substitution with ester enhanced both permeability and degradation potency, while methylation afforded two diastereomers with different permeability profiles. Steered molecular dynamics and enhanced conformational sampling in polar and nonpolar environments revealed distinct chameleonic behaviors, with the more permeable diastereomer **2b** adopting folded conformations with a lower solvent-accessible 3D polar surface area in the nonpolar environment. These findings were supported by 2D NMR and hydrogen-bond acidity analyses (*A*_NMR_). Notably, low-energy “congruent conformation” accessible in both environments was identified for **2b**. This work establishes a viable strategy for the design of membrane-permeable PROTACs.

## Graphical abstract

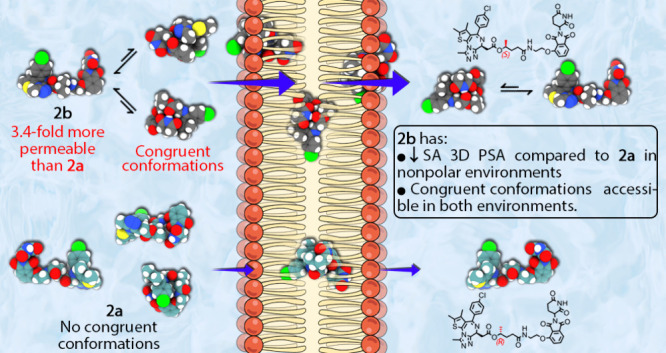

## Introduction

Targeted protein degradation (TPD) is a therapeutic approach that harnesses the body’s own cellular machinery to eliminate proteins, aiming to target hard-to-drug proteins through degradation rather than traditional inhibition.^1–3^ Among the various TPD strategies, proteolysis targeting chimeras (PROTACs) represent the pioneering modality and many PROTACs are currently in clinical trials, with at least three having advanced to Phase 3.^4–6^

However, the advancement of the majority of PROTACs toward clinical application and market approval is impeded by various pharmacodynamic and pharmacokinetic challenges. Since PROTACs act inside cells, they should have both good water solubility and adequate cell permeability. Owing to their hybrid structures consisting of three distinct components, PROTACs typically lie in the ″beyond Rule of 5″ (bRo5) chemical space, characterized especially by a molecular weight (MW) exceeding 500 Da and a polar surface area (PSA) greater than 200 Å^2^.^7^ This contributes to their limited water solubility and membrane permeability, as well as increased susceptibility to active transporter-mediated efflux, a mechanism through which multidrug resistance (MDR) can develop.^8,9^ Accordingly, studies addressing the poor drug-likeness of PROTACs are receiving increasing attention in the field.^10–17^

Lokey and co-workers recently suggested that amide-to-ester substitution is a promising approach for improving the permeability of bRo5 molecules.^18–20^ They applied this strategy to a cyclic hexapeptide model compound and achieved enhanced lipophilicity and membrane permeability compared to the amide counterpart. This was ascribed to the reduction of the total number of solvent-exposed amide NHs without significantly affecting the conformation, compared with the parent amide. *N*-Methyl (*N*-Me) substitution had a less pronounced effect than ester conversion. These strategies are predicted to enhance permeability by minimizing the energetic cost of desolvation—associated with hydrogen bond donation (as in the case of the amide NH)—when transitioning from an aqueous environment to the lipophilic interior of the cell membrane.^18,20,21^ The conversion of amides to esters did not impact the plasma stability of the cyclic peptides studied, as the macrocyclization effectively shields the ester bond from enzymatic degradation. Similarly, work from groups led by Lokey and Ciulli demonstrated that introducing amide-to-ester substitutions in a series of VHL-based BET degrading PROTACs led to enhanced permeability while maintaining intracellular stability.^18,19^

The conformational flexibility of bRo5 compounds is known to allow the reversible formation of environment-dependent intramolecular hydrogen bonds (IMHBs), which can help the molecules adapt to different biological environments, i.e., aqueous environments and lipophilic cellular membranes.^22^ This ability is referred to as ″molecular chameleonicity″, and bRo5 molecules with “chameleonicity” can adopt open, more polar conformations in aqueous environments, enhancing solubility, while transitioning into folded, less polar conformations as a result of shielding of the polar groups through intramolecular interactions within the nonpolar cellular membrane to enhance membrane permeability.^23^ Chameleonicity is well-established as a mechanism enabling the membrane permeation of high-molecular-weight compounds, including macrocyclic peptides and PROTACs.^24–26^

The impact of stereochemistry on molecular chameleonicity, permeability, and the formation of dynamic IMHBs is well-documented for various cyclic peptides and nonpeptide macrocyclic diastereomers.^27,28^ The fact that diastereomers can be separated by chromatography is a direct consequence of their different physical properties, such as polarity and solubility. These differences reflect variations in the intra- and intermolecular interactions, which can lead to differences in their conformational/chameleonic profiles and PK profiles. However, to our knowledge, no prior studies have explored the impact of stereochemistry on the PK profiles of PROTACs or the potential utility of the strategic introduction of chiral centers into PROTACs.

Here, we aimed to explore strategies to enhance the cellular permeability of PROTACs. Specifically, by using a BRD4-targeting PROTAC dBET1 and its ester derivatives as model compounds, we investigated the combination of the following two approaches: (1) amide-to-ester substitution, and (2) the induction of conformational changes via methyl group introduction to generate diastereomers with distinct physicochemical properties. We demonstrate that switching the amide to an ester improved the permeability without decreasing the stability compared to dBET1, in accordance with previous findings.^19^ All the ester derivatives showed stronger potency for BRD4 degradation than the parent compound dBET1, presumably driven by enhanced cell permeability. Interestingly, PK analyses showed that introduction of a methyl group adjacent to the ester functionality produced two diastereomers with dramatically different permeability. Computational analyses of the diastereomers provided insights into the reason for the differences in environment-responsive conformational change. Notably, our *in silico* conformational analyses suggest the existence of a congruent conformation that is likely responsible for permeation. These results indicate that combining amide-to-ester substitution of dBET1 and stereochemistry-driven induction of conformational change is an effective molecular design strategy for the development of permeable PROTACs.

## Results and Discussion

### Molecular Design

We hypothesized that introducing a methyl group on the carbon adjacent to ester oxygen in PROTACs would yield diastereomeric analogs with improved permeability. For proof of concept, we employed dBET1 ^29^ (Figure 1), a well-known BET bromodomain-targeting PROTAC, as a model. dBET1 is composed of BET inhibitor JQ1 (**4**, Scheme 1) conjugated to a linker via an amide bond and to a cereblon ligand, thalidomide, via an ether bond.

**Figure 1:**
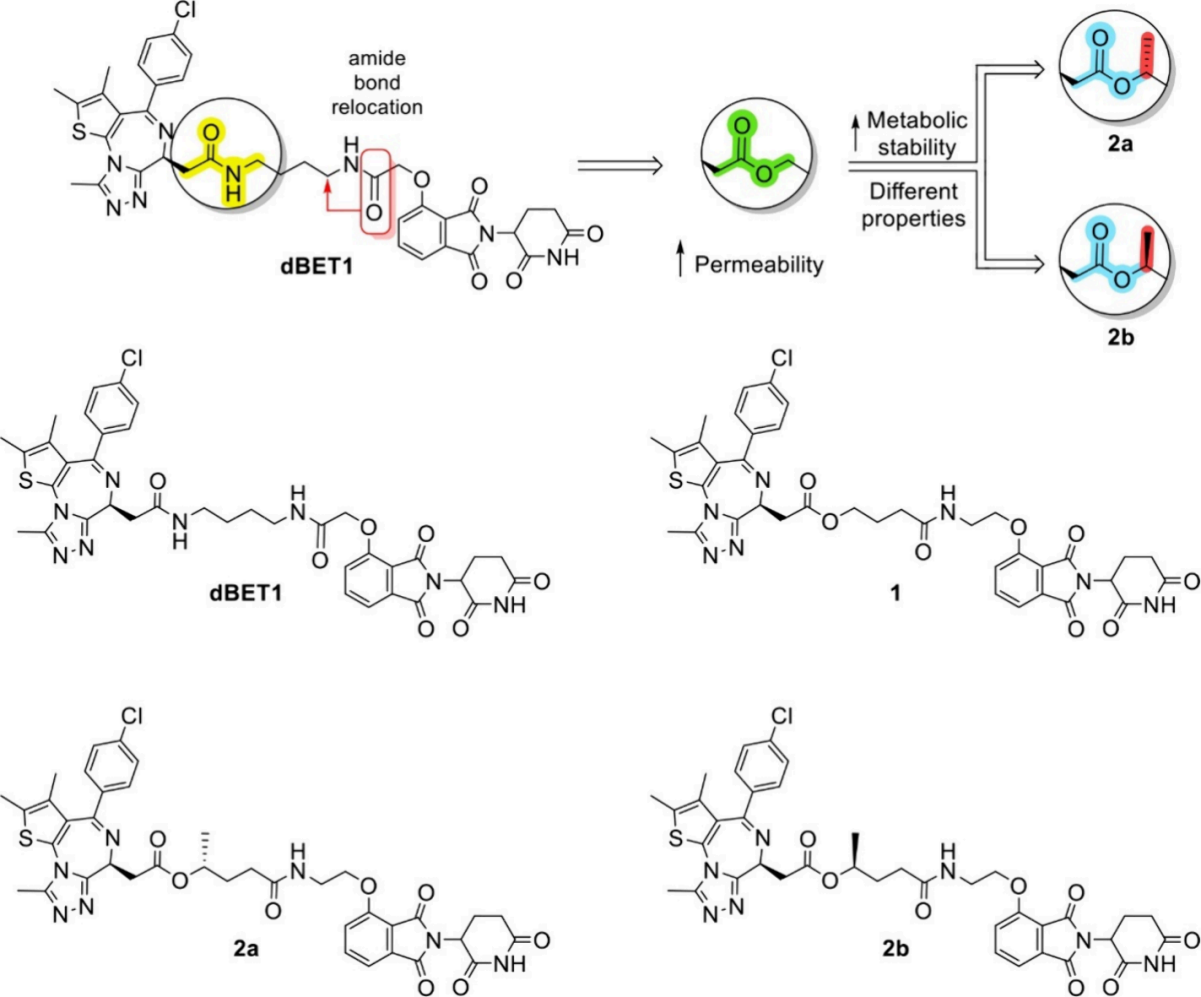
Molecular design and chemical structures of dBET1 and compounds **1** and **2**.

**Scheme 1:**
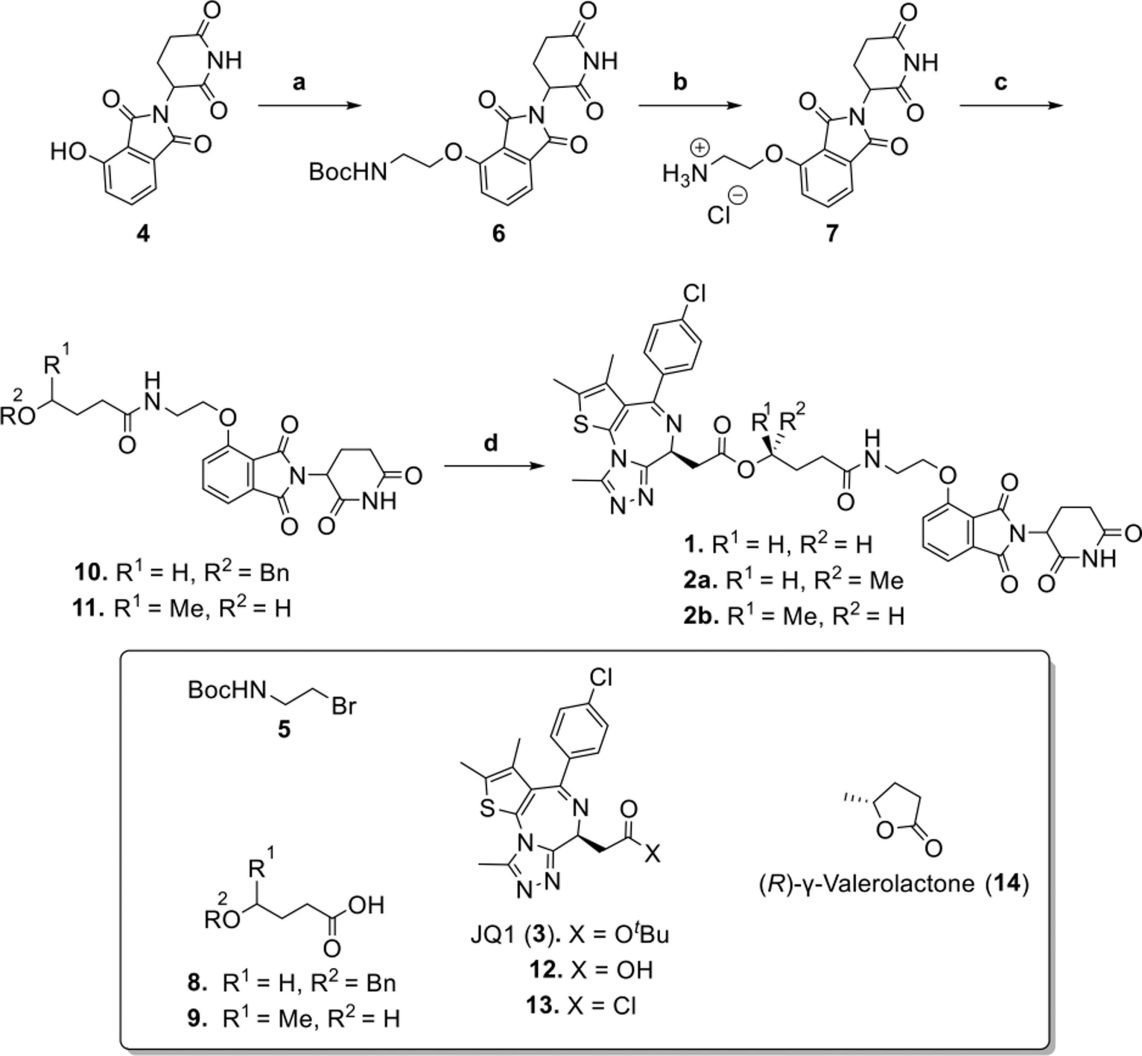
Synthesis of the Ester PROTACs **1**, **2a** and **2b** [Fn sch1-fn1]

a
**Reagents: a. 5**, KHCO_3_, DMF, 80°C, 24 h; **b.** HCl (in CPME), DCM, overnight, rt; **c.** **8** or **9**, HATU (5 equiv), DIEA (3 equiv), DMF, rt, 1.5 h; **d.** 1. **8**, Pd/C, H2, MeOH, 2. **12**, HATU, DIEA, DCM; or **9**, **13**, DIEA, DCM, rt, 24 h.

We selected the amide bond between JQ1 and the linker for conversion into an ester to follow the previous amide-to-ester conversion.^19^ Furthermore, we anticipated that the newly introduced chiral center near the ester would be sufficiently close to the chiral center in JQ1 to induce significant conformational changes, thereby influencing the properties of the resulting diastereomers. Our rationale stems from the principle that the resolution of diastereomers on achiral stationary phases is largely governed by the spatial proximity of their stereocenters; diastereomers with remote chiral centers often require chiral chromatographic methods because their three-dimensional differences are insufficient for effective separation.^25^ Based on the assumption that diastereomers separable on achiral columns are more likely to display distinguishable physicochemical properties, including membrane permeability, we directed our design strategy toward introducing structural modifications in the vicinity of the JQ1 chiral center to enhance their conformational divergence. Unfortunately, racemic, (*R*)-, and (*S*)-5-aminopentan-2-ol are not readily available and we had to design ester analogs with amide-bond relocation as indicated in Figure 1.

Therefore, we designed and synthesized compound **1** as an amide-to-ester analog and compounds **2a** and **2b** as branched ester analogs (Figure 1).

### Synthesis

Heating of 4-hydroxythalidomide (4) with compound **5** at 80 °C in the presence of KI and KHCO_3_ afforded compound **6**. Deprotection of the Boc group in **6**, followed by condensation with **8** or racemic **9** using HATU, provided compounds **10** and **11**, respectively. The benzyl group of **10** was removed via catalytic hydrogenation, followed by condensation with the carboxylic acid **12** to afford ester **1**. Meanwhile, the condensation of **11** with acid chloride **13** yielded a mixture of diastereomers **2a** and **2b** which were separated by preparative HPLC (Scheme 1). The configuration of the newly introduced chiral center was determined by repeating the synthesis using enantiomerically pure (*R*)-γ-valerolactone (**14**), which was hydrolyzed, then condensed with **7** and treated with acid chloride **13** to afford **2a**.

### Evaluation of BRD4-Degrading Activity and PK Profile

We evaluated the BRD4 degradation activity of dBET1 alongside its ester analogs **1, 2a** and **2b** by Western blot (Figure 2, Figure S1, and Table 1). Compared to dBET1, all ester-based PROTACs exhibited significantly lower half-maximal degradation concentration (DC_50_) values. Compounds **2a** and **1** showed the greatest improvement, with DC_50_ values of 16 nM and 19 nM, respectively, representing approximately 4- to 5-fold enhancement compared to the DC_50_ of 84 nM for dBET1. Although slightly less potent than its counterparts, **2b** still achieved a noteworthy improvement, showing a DC_50_ of 43 nM, representing a 2-fold increase in degradation activity relative to dBET1. The hook effect was observed for all these PROTACs. To quantitatively compare the degree of the hook effect, we calculated the Hill coefficients of dose–response curves fitted across the high-concentration range exhibiting this effect (Table S1). The Hill coefficients, i.e. degree of the hook effect, showed variations between compounds, even among the diastereomers **2a** and **2b**. These results were confirmed by HiBiT-BRD4 assay,^30^ which gave similar results (Figure S2).

**Figure 2:**
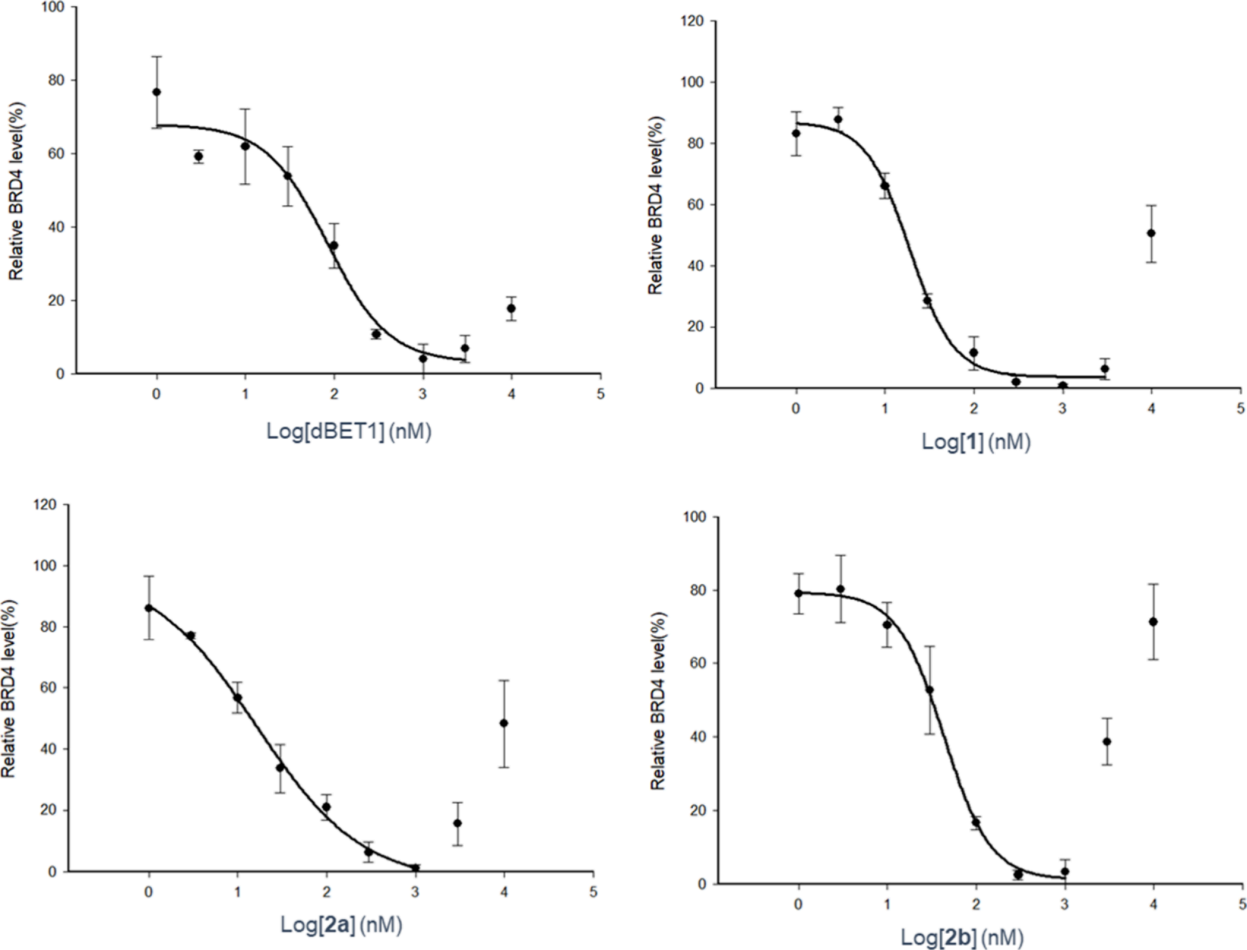
BRD4 degradation-inducing activity of dBET1 and its ester analogs. MCF-7 cells were treated with each compound at 10, 30, 100, 300, 1000, 3000, and 10000 nM for 4 h, followed by Western blot analysis of BRD4 abundance in the cells. Plots show the mean ± SE of three independent experiments, normalized to DMSO control. The curve was fitted to the data points excluding high-concentration range showing the hook effect.

**Table 1:** Degradation Activity, Permeability, and Stability Evaluation

					metabolic stability^a^			
	degradation activity	solubility [μM]		PAMPA	NADPH (+)		NADPH (−)	stability in media^b^
compound	DC_50_ ± SE (nM)	JP1 (pH 1.2)	JP2 (pH 6.8)	P_app_ [×10^–6^ cm/s]^c^	% remaining @ 30 min	CL_int,u_ [mL/min/kg]	% remaining @ 30 min	% remaining @ 6 h
dBET1	84 ± 1.4	>100	83.7	0.149	1.33	2876	86.7	14.2
**1**	19 ± 1.1	90.8	84.9	0.437	6.75	1785	86.7	40.7
**2a**	16 ± 1.6	87.6	82.6	0.348	1.70	2702	>90	>90
**2b**	43 ± 1.2	87.4	80.2	1.20	1.08	3034	>90	>90

a Stability in mouse liver microsomes.

b Stability in culture media supplemented with 10% FBS.

c Mean of two independent experiments.

Next, we evaluated the cell permeability and metabolic stability of our PROTACs. PAMPA assay showed that esters **1** and **2a** exhibited approximately 1.6- to 2.7-fold higher membrane permeability than dBET1 (Table 1). On the other hand, **2b** gave a P_app_ of 1.20, showing approximately 8-fold higher permeability than dBET1. The P_app_ value of **2b** is comparable to that of metoprolol (see Experimental Section), an FDA reference drug commonly used to classify drugs into low and high permeability categories, suggesting that **2b** possesses moderate permeability. Notably, **2b** is 3.4-fold more permeable than its diastereomer **2a**. This interesting finding underscores how even a subtle structural difference, such as the configuration of the chiral center bearing the methyl group, can profoundly impact the permeability of these highly flexible molecules.

As summarized in Table 1, all compounds exhibit relatively good aqueous solubility at both JP1 (pH 1.2) and JP2 (pH 6.8), with values close to or exceeding 80 μM, indicating that solubility is unlikely to be a limiting factor for cellular activity. Despite this, no clear correlation is observed between PAMPA permeability and degradation activity among the ester analogs. For example, compounds **1** and **2a** display the most potent degradation activities (DC_50_ = 19 and 16 nM, respectively), yet their permeability values are lower than that of compound **2b**, which shows the highest permeability but weaker degradation activity (DC_50_ = 43 nM). These observations indicate that passive membrane permeability is not predictive of cellular degradation activity in this series. This lack of correlation is consistent with the limitations of PAMPA,^31^ which does not account for active transport or efflux processes that can critically influence intracellular concentration. Together, these results suggest that while the enhanced passive permeability of the three analogs may contribute to their differences in biological activity compared to dBET1, the variations among the analogs themselves are likely driven by other factors beyond passive permeability play dominant roles in determining cellular degradation activity such as binding affinity, ternary complex stability and active uptake and efflux transportation.

To explore the possibility of different binding affinities toward BRD4 as a contributing factor, we performed a molecular docking study using JQ-1 s-amyl esters with both configurations at the chiral center, reflecting the stereochemical environment of **2a** and **2b**. The results showed very similar docking scores (Figure S4). This suggests that binding affinity alone is unlikely to account for the activity difference.

To validate the permeability data and to assess the potential contribution of active uptake and efflux transport processes, the intracellular concentrations of these PROTACs were quantified. (Figure S3). At the 0-h time point, where the medium containing the compounds was removed immediately post-treatment and the cells were washed, the measured concentrations reflect the amounts of the compounds adsorbed on the cell membrane. At this point, all ester PROTACs were detected at higher concentrations than dBET1, indicating superior membrane adsorption, presumably because of their increased lipophilicity. The data at the 0.5-h time point was considered to reflect the true membrane permeability of the compounds. At this time point, all ester analogs exhibited higher intracellular concentrations than dBET1. Consistent with the PAMPA assay results, **2b** demonstrated the highest intracellular concentration, followed by **2a** then **1**, with dBET1 showing the lowest concentration. At the 6-h time point, intracellular concentrations would be influenced by multiple factors, including membrane permeability, intracellular stability, and transporter-mediated efflux. Thus, the measured concentrations at 6 h reflect the dynamic equilibrium among these processes. The intracellular concentrations of dBET1, **1** and **2b** were lower at the 6-h interval than at the 0.5-h interval. In contrast, **2a** displayed an increase in intracellular concentration over time, suggesting better stability and reduced efflux, which may enable accumulation in an intracellular compartment. Notably, both diastereomers, **2a** and **2b**, exhibit comparable intracellular levels at 6 h, and these levels are markedly higher than those observed for dBET1 and compound **1** (Figure S3). Despite its lower intracellular concentration, compound **1** exhibited robust degradation activity, further demonstrating that membrane permeability alone does not dictate biological efficacy.

Returning to the hook effect data (Table S1), a positive correlation was observed between the degree of the hook effect and the compound’s permeability. Although the hook effect is theoretically attributed to the excessive local concentration of PROTACs, this study to our knowledge provided the first experimental evidence confirming this correlation.

To assess the metabolic stability of the compounds, we conducted mouse liver microsome metabolic stability and FBS (fetal bovine serum) stability assays (Table 1).

In the metabolic stability assay, the compounds were evaluated in the presence and absence of NADPH to assess the contribution of cytochrome P450-mediated metabolism (NADPH­(+)) versus noncytochrome P450 enzymatic metabolism (NADPH(−)). Under the NADPH­(+) condition, all compounds exhibited low residual percentages, indicative of significant metabolism. Notably, **1** demonstrated slightly better stability compared to the others, though the reason for this unexpected observation remains unclear. In contrast, under the NADPH(−) condition, both diastereomers **2a** and **2b** displayed slightly greater stability than the other compounds. Next, the stability of these PROTACs in medium containing 10% FBS was evaluated over a 6-h period, corresponding to the incubation time used in the BRD4 degradation activity assays. All ester PROTACs exhibited higher residual percentages compared to dBET1, indicating greater stability in the culture medium. Interestingly, **1** also demonstrated improved stability relative to dBET1, which displayed the lowest stability among the tested compounds. The relocation of amide may account for this observation. In dBET1, the phenolic oxygen positioned on the α-carbon of the amide imposes a relatively stronger electron-withdrawing (negative inductive) effect compared to the other compounds. This difference could contribute to altered stability and may therefore contribute to the observed differences in behavior. Collectively, in accordance with our hypothesis, these findings highlight that introducing a new chiral center in proximity to the chiral center in JQ-1 generates two diastereomers (**2a** and **2b**) with good stability and distinct permeability profiles.

### The Enigma of the Diastereomeric Difference in Membrane Permeability

Why does **2b** show superior membrane permeability compared to its diastereomer **2a**? The permeability difference between dBET1 and its ester analogs can be attributed to the amide-to-ester substitution, which reduces the energetic cost of desolvation during the transfer from an aqueous environment to the lipophilic cell membrane. In addition, relocating the amide may influence permeability by enabling the formation of favorable IMHBs in nonpolar environments where such interactions were previously disfavored, thereby potentially facilitating membrane passage relative to the parent dBET1. Although lipophilicity is an important contributor to passive permeability, it is not the sole determinant. For instance, while diastereomers **2a** and **2b** are more lipophilic than compound **1**, **2a** exhibits slightly lower permeability than **1**, whereas **2b** shows higher permeability. More importantly, **2a** and **2b** themselves display a pronounced difference in permeability. These observations indicate that, within the bRo5 chemical space, additional factors such as molecular chameleonicity play a substantial role in determining cellular permeability. Consequently, the observed difference in passive permeability between **2a** and **2b** is likely driven primarily by differences in their molecular chameleonicity. To investigate this, we applied three computational techniques, summarized in Table 2. We began with steered molecular dynamics (SMD) simulation^15,32^ to generate conformational ensembles of **2a** and **2b** in explicit water, toluene, and a 1:1 DMSO-water solvent system. We focused on the radius of gyration (*R*_gyr_) as a descriptor of molecular size and the solvent-accessible 3D polar surface area (SA 3D PSA) as an indicator of polarity that reflects molecular conformation.

**Table 2:** Summary of Computational Methods and Key Conformational Findings

method	purpose	findings
SMD simulation	generation of conformational ensembles of both diastereomers in toluene and water	Both compounds adopted more folded conformations in toluene compared to water. The folded conformations of **2b** demonstrated lower SA 3D PSA in toluene than **2a**. Common conformations were identified for **2b** in both solvents.
iMTD-GC conformational search	generation of low-energy conformers in water and toluene	identified low-energy, congruent conformations for compound **2b**
LowModeMD conformational search	generation of low-energy conformers in water and toluene	supported the findings of the iMTD-GC conformational search

The analysis of density distributions derived from SMD conformers suggests that **2b** exhibits molecular chameleonicity, as indicated by the clustering of its conformers in toluene within regions characterized by lower *R*_gyr_ and reduced SA 3D PSA compared to those in water, where the molecule adopts more extended (semifolded) conformations, as shown in Figure 3a,b. Around 90% of **2b** conformations in toluene have an SA 3D PSA below 215 Å, whereas in water, only around 25% of the population exhibits values below this threshold (Figure 4a). Additionally, all **2b** conformations in toluene have an *R*_gyr_ lower than 5.5 Å, while only about 25% of the water ensemble conformations fall below this value (Figure 4a). These results show that **2b** adopts polar and extended conformations in aqueous environments, whereas in toluene, the molecule exhibits more compact and hydrophobic behavior.

**Figure 3:**
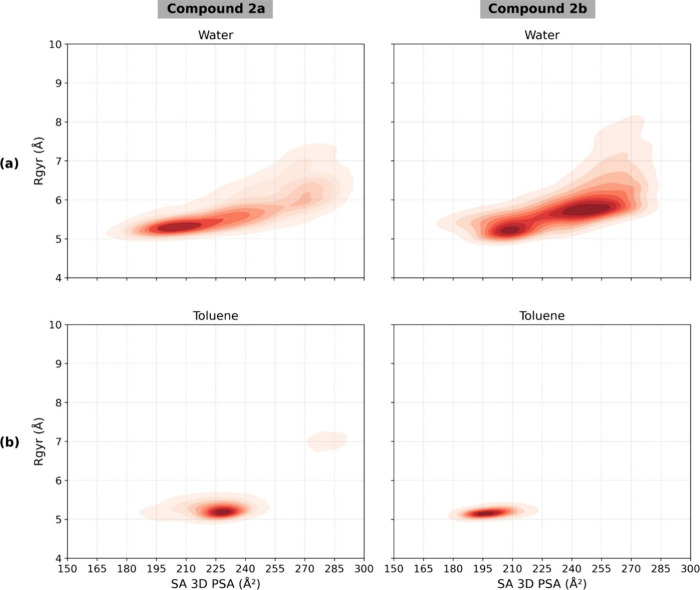
A comparative analysis of the conformational ensembles of **2a** and **2b** generated by SMD. The plots show the relationship between *R*_*g*yr_ and the SA 3D PSA. Data are presented for **2a** (left column) and **2b** (right column) simulated in water (**a**) and toluene (**b**). Darker red regions indicate a higher population density of conformations.

**Figure 4:**
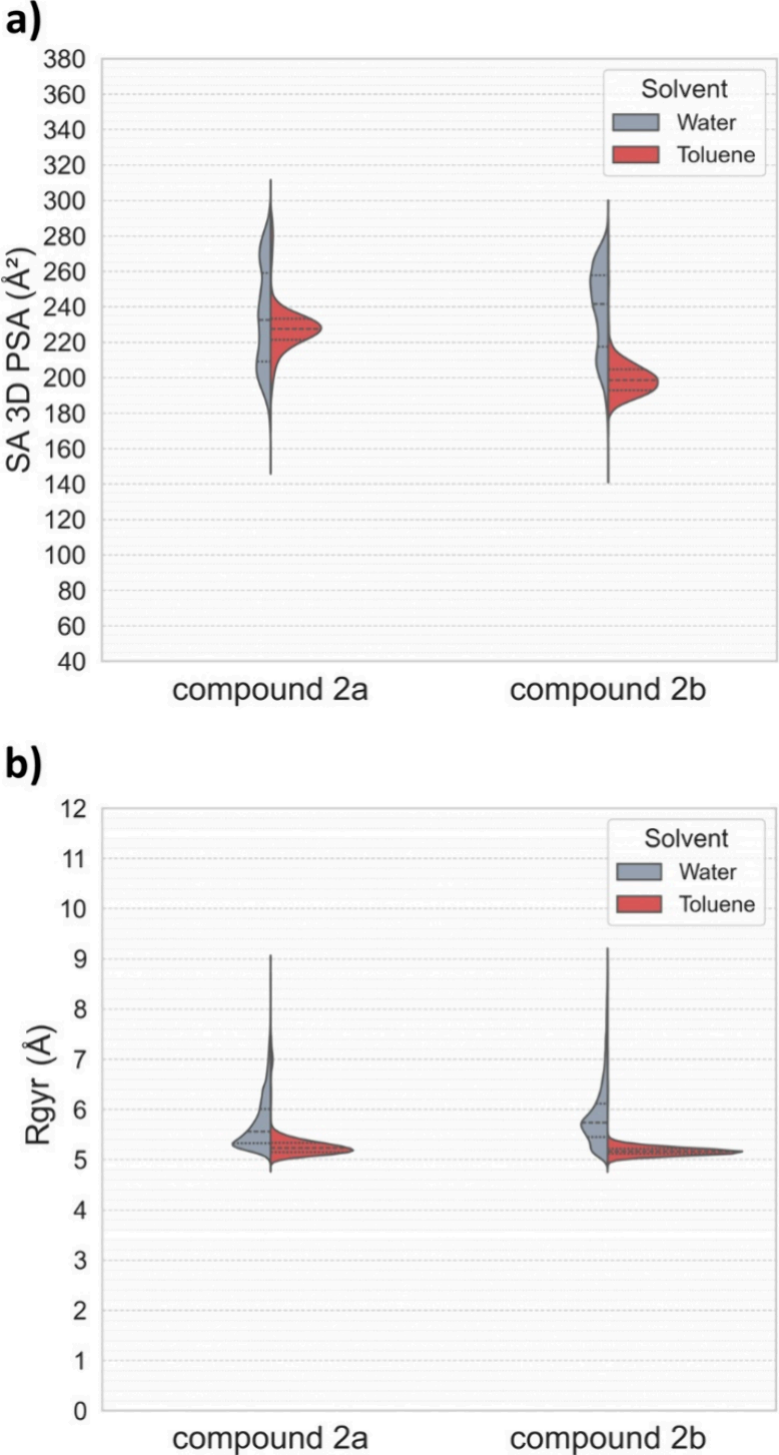
Distribution of key structural descriptors for **2a** and **2b** in water and toluene. The violin plots compare the distributions of SA 3D PSA (**a**) and *R*_*gyr*_ (**b**) for the two compounds. The width of each plot represents the probability density of the observed values in water (gray) or toluene (red). Dashed lines inside the violins indicate the quartiles.

Further inspection of the conformations obtained from each ensemble and the clusters obtained shows that in toluene, **2b** predominantly (>90%) adopts a folded conformation stabilized by dynamic intramolecular interactions that are not observed in the water ensemble. These include hydrogen bonding between the amide NH in the linker and the imide C = O in thalidomide, as well as n−π* interactions involving lone-pair electrons of triazole basic nitrogen atoms and the π* orbital of phthalimide C = O. Notably, this interaction satisfies the Bürgi–Dunitz angle conditions in approximately 50% of the population (Figure 5a). Additionally, T-shaped π-stacking interactions (Figure 5b) contribute to structural stabilization by minimizing the exposure of polar functional groups to the hydrophobic environment. The methyl group around the ester further contributes to this stability by forming a C–H/π interaction with the chlorophenyl ring.

**Figure 5:**
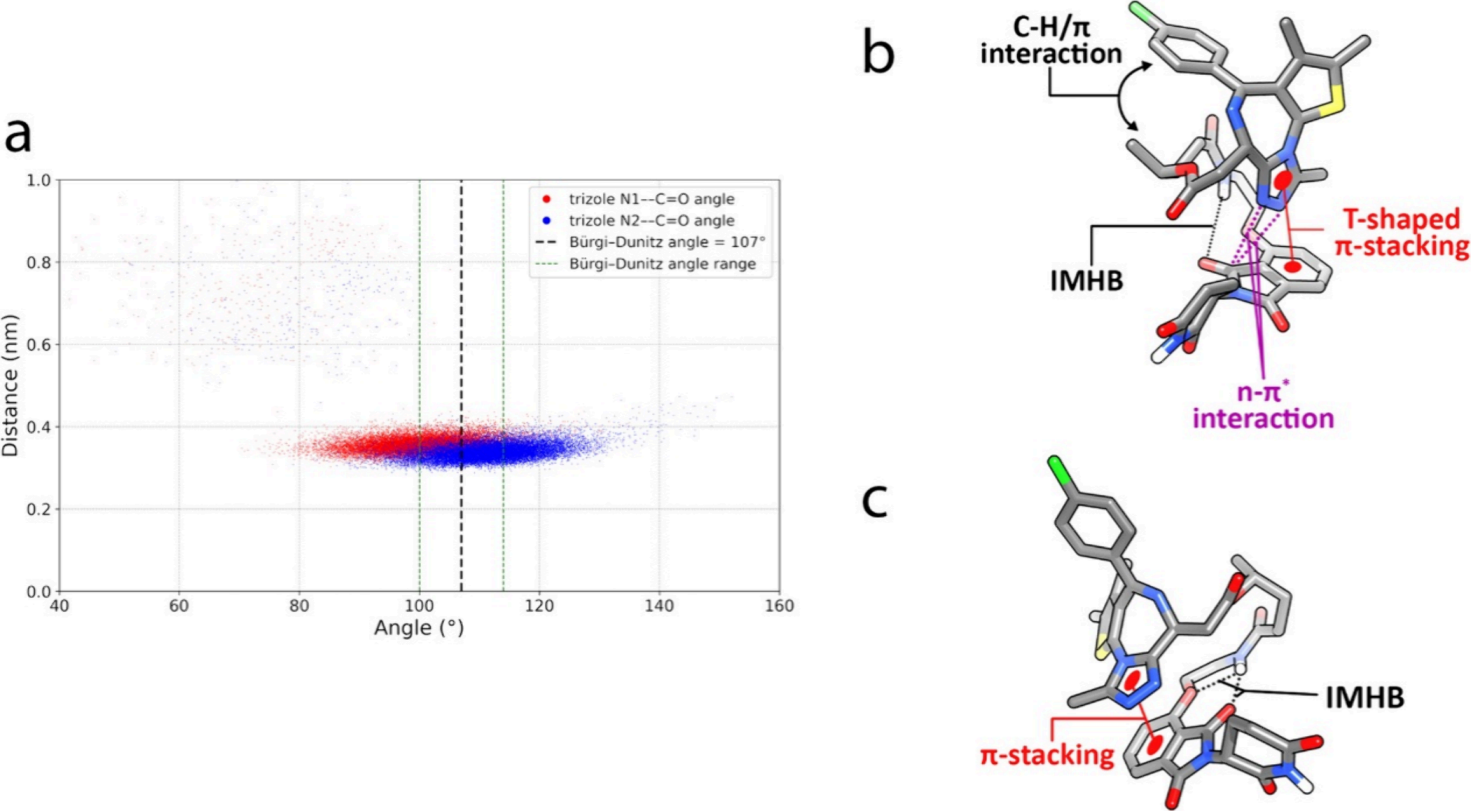
**a.** Scatter plot of Bürgi–Dunitz angle-distance between triazole basic nitrogen atoms and phthalimide C = O of compound **2b** illustrated as n-π* interaction in part **b** of this figure. **b.** The dominant conformation of compound **2b** in the toluene ensemble generated by SMD. **c.** The dominant conformation of compound **2a** in the toluene ensemble generated by SMD.

The results also indicate that **2b** tends to favor semifolded S-shaped (conformers no. 5, 7, and 10) and U-shaped (conformers no. 1, 2, 4, 6 and 9) conformations (Figure S5) in water, rather than adopting linear conformations (conformer no. 3). This preference can be attributed to the competition between intermolecular hydrogen bonding with water and intramolecular interactions within **2b**.

The density plots suggest that compound **2a** also displays chameleonic behavior to some extent (Figure 3a,b). However, the influence of its conformational changes on SA 3D PSA in toluene is significantly less pronounced than that observed for compound **2b**. In water, the conformer distributions of both **2a** and **2b** are closely aligned in terms of SA 3D PSA and *R*_gyr_, as shown in Figures 3a and 4a. That said, compound **2a** tends to preferentially occupy a region where *R*_gyr_ ranges from 5.0 to 5.5 Å and SA 3D PSA falls between 185 and 230 Å^2^, accounting for approximately 50% of the population. In contrast, compound **2b** is more concentrated in regions with higher values for both properties. Although compact population of **2a** is observed in water, this behavior is distinct from hydrophobic collapse.^33^ Analysis of the molecular property space (Figure 4a,b) shows that **2a** maintains a broad and dynamic conformational ensemble, with a significant population exhibiting high SA 3D PSA and increased *R*_gyr_, in contrast to the more restricted ensemble in term of those descriptors observed in toluene. Rather than forming a kinetically trapped collapsed state, **2a** displays reversible and dynamic conformational sampling characteristics of a flexible, solvated molecule in water. The aqueous solubilities of **2a** and **2b** are comparable (Table 1), and NOESY data show similar solution behavior for both compounds in DMSO (see below), consistent with this interpretation.

In toluene, both compounds adopt more folded conformations, as indicated by their lower *R*_gyr_ values (Figures 3b and 4b). However, compound **2b** exhibits significantly lower SA 3D PSA values, with approximately 95% of its conformational population falling within the range of 180 to 220 Å^2^. In contrast, its diastereomer **2a** displays SA 3D PSA values ranging from 200 to 240 Å^2^, even though it showed more frequent IMHBs in the toluene ensemble (Table 3). This suggests that IMHBs is not the only factor that contributes to lowering the SA 3D PSA, and other intramolecular interactions also contribute. The results also suggest that compound **2b** exhibits more efficient chameleonic behavior, with a greater ability to adopt less polar conformations in toluene, characterized by lower SA 3D PSA values. This property and the low *R*_gyr_ of the toluene ensemble are likely the key factors contributing to the observed differences in permeability between the two diastereomers.

**Table 3:** IMHBs Frequency in the SMD Conformational Ensembles

	in water		in toluene	
compound	1 IMHB	2 IMHBs	1 IMHB	2 IMHBs
**2a**	26.2%	8.2%	36.4%	6.4%
**2b**	7%	0%	21.5%	0.5%

Structurally, the polar groups of **2a** tend to be shielded from toluene through the formation of IMHBs, with the conformations being further stabilized by π-stacking interactions (Figure 5c). In water, compound **2a** adopts semifolded U- and S-shaped conformations, similar to those of compound **2b**, as seen in conformers 2, 3, 4, 5, 6, and 9 in Figure S6. However, compound **2a** forms IMHBs more frequently than **2b** in the water ensemble (Table 3), which likely explains the tendency for its SA 3D PSA values to accumulate around the 185–230 Å^2^ range (Figure 3a).

Overall, the conformational ensembles of both diastereomers reveal varying degrees of chameleonicity. While both compounds adopt semifolded conformations in water and compact folded conformations in toluene, compound **2b** shows significantly lower polarity in toluene compared to **2a**, despite similar degrees of folding. These results are consistent with experimental data showing comparable water solubility (Table 1), but enhanced membrane permeability for compound **2b**.

These findings were further supported by simulation in a 1:1 DMSO-water system, which is expected to disrupt all types of intramolecular interactions in both diastereomers **2a** and **2b**. Indeed, this effect was clearly observed in the theoretical conformation ensemble of **2b** (Figures S7 and S8b), where **2b** predominantly adopts extended and linear conformations compared to those in water. The clustering results in the DMSO-water ensemble show that more than 50% of **2b** conformations are fully linear (conformers no. 1, 3, 5, 6, and 10 in Figure S9). For **2a**, as in the case of **2b**, the DMSO-water system facilitates the shift toward more semifolded and extended conformations, exposing some hydrophobic groups that were previously shielded in pure water (Figure S10). However, more than 75% of the conformers of **2a** in the DMSO-water ensemble remains within the region of *R*_gyr_ lower than 7 Å compared to less than 50% of the conformers of **2b** (Figure S8b), suggesting that **2a** has a greater tendency than **2b** to maintain some semifolded conformations in polar solvents. This indicates the presence of stronger intramolecular interactions in **2a** that resist polar solvent-induced conformational unfolding. It is expected that the equilibrium shift from semifolded to unfolded conformations is highly dependent on the solvent’s ability to overcome the intramolecular forces that stabilize each molecule’s semifolded conformation. Consequently, the observed greater extent of this equilibrium shift in diastereomer **2b** evidenced by its higher *R*_gyr_ in the DMSO-water ensemble suggests superior conformational adaptability and better dynamic exposure of polar regions to the solvent environment.

To further validate our observations and confirm reproducibility, a second run of SMD simulations in water and toluene was performed. Notably, the second run reproduced the results of the first run, producing overall highly similar molecular property spaces with only minor differences in toluene and water (Figure S11). Interestingly, compound **2b** in the second replicate occupied a distinct region of the molecular property space, characterized by lower SA 3D PSA and *R*_gyr_ values, with around 4.4% of the ensemble falling within the PSA range recommended by Veber’s rule for good membrane permeability.^34^ The lowest observed SA 3D PSA for this compound was 126.5 Å^2^. This highlights the ability of compound **2b** to adopt significantly more lipophilic conformations in nonpolar environments compared to compound **2a**.

The findings of the SMD simulations highlight how solvent-induced changes in intramolecular interactions control the conformations of both **2a** and **2b**, ultimately influencing their cell membrane permeability behavior. The trends observed in *R*_gyr_ and SA 3D PSA in different SMD ensembles for both diastereomers align well with the experimental permeability results. It is worth emphasizing that the conformational differences between diastereomers **2a** and **2b** are also clearly reflected in their NOESY spectra recorded in CDCl_3_ and DMSO-*d*_6_. In CDCl_3_, both compounds display long-range NOE correlations consistent with more folded conformations compared to DMSO-*d*_6_ (Figure 6). However, the specific long-range NOE patterns observed in CDCl_3_ differ between the two diastereomers, indicating that each adopts a distinct folded conformation. This observation is in excellent agreement with the SMD simulation results.

**Figure 6:**
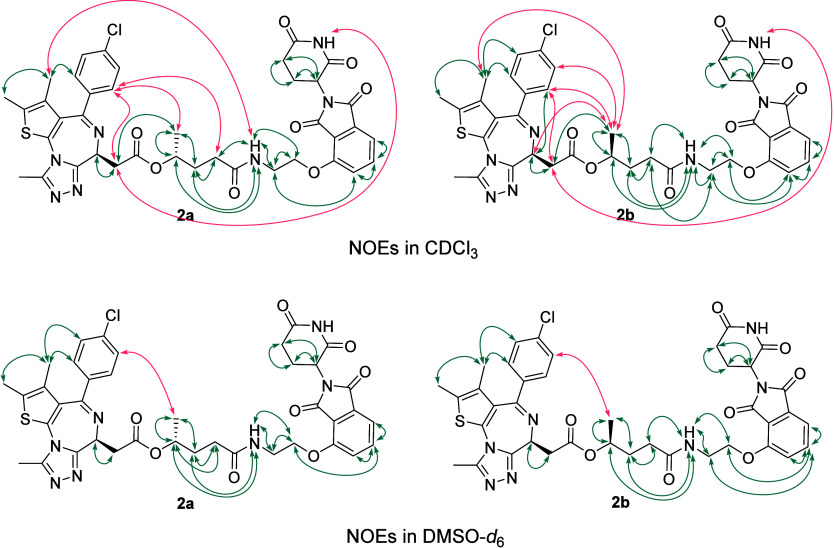
Overview of the experimentally determined nuclear Overhauser effects (NOEs) of both diastereomers (2a and 2b) in CDCl_3_ and DMSO-*d*_6_, highlighting long-range (red) and short-range (green) NOE correlations.

In contrast, the NOE correlations detected in DMSO-*d*_6_ are relatively similar for both compounds, suggesting that they adopt overall comparable conformations in this solvent. Notably and interestingly, both diastereomers exhibit very similar ^1^H NMR spectra in DMSO-*d*_6_, whereas their spectra diverge in CDCl_3_ and MeOH-*d*_4_ (Figures S45–S47). This behavior can be rationalized by the ability of both molecules to access linear or semilinear conformations in DMSO-*d*_6_, while shifting toward different more folded conformations in less polar solvents. A similar trend is observed when comparing the ^1^H NMR spectra of compound **1** in DMSO-*d*_6_ and CDCl_3_ with those of compounds **2a** and **2b**, which show a striking overall similarity in DMSO-*d*_6_ with differences confined mainly to the signals associated with the carbon bearing the additional methyl group, but display pronounced spectral differences in CDCl_3_ (Figures S45 and S47).

The SMD simulations accurately captured this flexibility, predicting predominantly linear and semifolded conformations in polar solvents and more compact, folded conformations in nonpolar environments. Importantly, the changes in *R*_gyr_ observed across the SMD ensembles for both diastereomers show strong consistency with NOESY data. Furthermore, the Δδ values (δDMSO – δCDCl_3_) and the hydrogen-bond acidity descriptor (*A*_NMR_), previously established as reliable metrics for identifying and quantifying IMHB involving OH and NH protons,^35^ provide additional insight into the behavior of the linker amide NH of the diastereomers. For **2b**, both Δδ (0.64 and 0.58 ppm, calculated from two measurements of the sample dissolved in CDCl_3_ on different days) and *A*_NMR_ (0.09 and 0.08) are notably lower than those of compounds **2a** (Δδ = 1.1 and 1.0 ppm; *A*_NMR_ = 0.15 and 0.14) and **1** (Δδ = 0.91 ppm; *A*_NMR_ = 0.13). Compounds with *A*_NMR_(NH) < 0.05 are known to exhibit strong IMHBs, whereas values >0.15 are characteristic of the absence of IMHBs. Our NMR analysis therefore indicates that the linker amide NH of **2b** engages in stronger IMHB compared with **2a** and **1**, whose values lie near the boundary of the threshold. These findings align well with our SMD results, which show that **2b** adopts conformations exhibiting lower SA 3D PSA relative to **2a,** and with the higher reverse-phase HPLC (RP-HPLC) retention time (RT) of compound **2b**.

Next, we performed a conformational search for compounds **2a** and **2b** using the iterative metadynamics and genetic crossing method (iMTD-GC) workflow in CREST, aiming to generate ensembles of low-lying conformers and determine the lowest-energy conformer, i.e., the global minimum structure, in both implicit water and toluene. We also sought to identify possible low-energy congruent conformations of these compounds across the two solvents.

CREST employs metadynamics in combination with GFN2-xTB, enabling it to push the molecule out of local minima and explore new geometries. It applies a bias potential to specific collective variables, which helps the search avoid revisiting previously sampled conformers and escape shallow energy wells. This exploration proceeds in multiple rounds, with each round using the most promising structures from the previous one to probe new regions of conformational space.^36^

The results revealed that the lowest-energy conformers of compound **2b** in water and toluene were nearly identical (Figure 7a), with a root-mean-square deviation (RMSD) of only 0.1 Å. In contrast, compound **2a** showed greater structural variability in both solvents within a 4 kcal/mol window from its global minimum conformer. This conformational similarity of **2b** ensembles suggests a common “congruent” conformation between the two environments.^37,38^ The significance of this lies in the reduction of the energetic penalty that compound **2b** would otherwise incur during transitions between different solvents, thus potentially contributing to its better membrane permeability. It is worth noting that this conformation was also identified as the most stable one through an independent conformational search in implicit water and the fourth most stable one in toluene using the LowModeMD method implemented in MOE software (Figure 7b,c). The congruent conformation of compound **2b** features two IMHBs: one between the imide NH and the ester carbonyl, and the other between the amide NH and the triazole ring of JQ-1. These interactions effectively shield the NH groups, contributing to a reduction in the SA 3D PSA. This lower PSA and the low energetic penalty facing the transition between different environments are associated with improved membrane permeability, which likely facilitates more efficient lipid membrane crossing.

**Figure 7:**
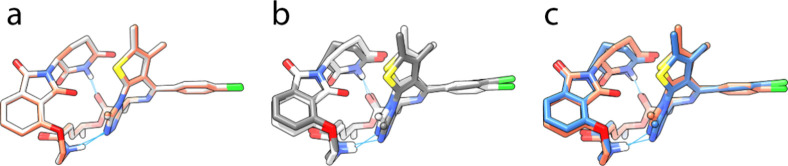
Overlay of (**a**) the most stable conformers of compound **2b** generated by iMTD-GC in implicit water (white) and toluene (salmon), (**b**) the most stable conformers of compound **2b** generated by iMTD-GC (white) and LowModeMD (gray) in implicit water, and (**c**) the most stable conformer of compound **2b** generated by iMTD-GC (salmon) and the 4th most stable one generated by LowModeMD (blue) in implicit toluene. IMHBs are shown as sky blue lines in all these models. In (**a**), the conformers superposed almost completely.

Furthermore, a comprehensive conformational similarity search, based on RMSD, for the SMD ensembles supported the previous findings. The analysis revealed a notable overlap in the conformational landscapes of compound **2b** in the water and toluene ensembles. After excluding the initial linear conformations, it was found that approximately 0.5% of the conformations within the water ensemble exhibited a high degree of structural similarity to a subset of the toluene ensemble (Figure 8a,b), with RMSD values below 1 Å. This subset of highly similar conformations constitutes approximately 3.3% of the entire toluene ensemble, strongly supporting the existence of congruent conformations accessible in both polar and nonpolar environments. In contrast, compound **2a** showed minimal conformational similarity in the two solvents. A comparison of its SMD ensembles, each containing 20,000 conformations, identified only a single conformation from the water ensemble that resembled a mere four conformations in the toluene ensemble. It is important to note that the subset of congruent conformations suggested by SMD differs from those generated by our iMTD-GC and LowModeMD conformational searches. This difference is likely due to the use of different force fields and solvent treatments, as SMD uses an explicit model while the other methods rely on implicit solvation. Nevertheless, both approaches consistently indicate a high probability of congruent conformations existing within the low-energy ensembles.

**Figure 8:**
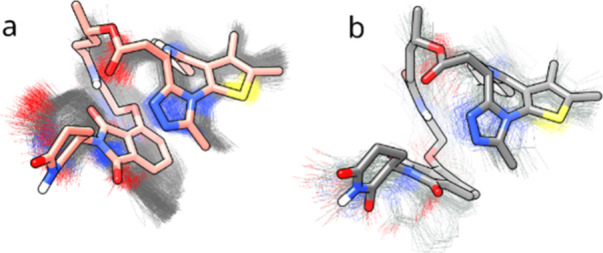
Visualization of the congruent conformational space identified by SMD for compound **2b**. The figure displays the subsets of conformations possessing an RMSD less than 1.0 Å from (a) the toluene ensemble and (b) the corresponding water ensemble. A representative structure for each subset is highlighted in stick style.

Taken together, the combined results from SMD, iMTD-GC and LowModeMD provided valuable insight into the dynamic conformational behavior of compound **2b**. In aqueous solution, it adopts a diverse set of conformations, including extended and semiextended forms. Notably, it can readily access the subset of congruent conformations, which is proposed to play a central role in its membrane permeability. This conformation subset facilitates the transition of compound **2b** into the lipid membrane, where it adopts a more folded conformation that is favored within the hydrophobic environment. Upon exiting the membrane, it can revert to the congruent conformations to re-enter the aqueous phase without incurring substantial energetic penalties during the transition. This conformational flexibility likely underlies the improved membrane permeability observed for compound **2b**, in contrast to compound **2a**.

## Conclusion

To address the issue of poor drug-likeness of PROTACs, we explored a strategy for enhancing the membrane permeability by employing the combination of two approaches (1) amide-to-ester substitution, and (2) the induction of conformational changes. We designed and synthesized three dBET1 ester analogs, **1**, **2a**, and **2b**, and assessed their degradability, membrane permeability, and stability. All three analogs exhibited better degradation. Further, analogs containing an extra methyl group demonstrated greater chemical stability relative to both dBET1 and **1**. Notably, all the ester analogs displayed better membrane permeability, and **2b** was classified as moderately permeable, exhibiting 3.4-fold higher permeability than its diastereomer **2a**. To investigate the origin of the diastereomeric difference in permeability, we performed SMD simulations in different solvents and analyzed conformation-dependent properties. The SMD analysis revealed that compounds **2a** and **2b** display different levels of chameleonicity, with **2b** exhibiting a stronger tendency to modulate its conformation to more polar ones in water and less polar ones in toluene. This difference in ability to adopt folded conformations characterized by low SA 3D PSA likely contributes significantly to the differences in permeability observed between the two diastereomers. These observations are supported by 2D NMR analyses together with calculations of the hydrogen-bond acidity descriptor (*A*_NMR_) for the amide NH in both diastereomers. Two distinct conformational searches (iMTD-GC and LowModeMD) revealed the existence of congruent conformations of **2b** among its lowest-energy conformers in both water and toluene, whereas this was not the case for **2a**. An overlap between the SMD water and toluene ensembles of compound **2b** further supports the presence of a subset of congruent conformations. This subset enables compound **2b** to experience a lower energetic penalty when transitioning between different solvents, which could also explain its improved membrane permeability compared to its diastereomer **2a**.

In parallel with our findings, Caron and co-workers have shown that linker methylation can enhance the oral bioavailability (F%) of VHL-based PROTACs, attributing this effect to methylation-induced chameleonic folding and its influence on efflux behavior.^39^ It should be noted that their study did not extensively examine the stereochemical effects, whereas the present work specifically focuses on how stereochemistry modulates conformational ensembles and molecular properties. In addition, our study does not evaluate full pharmacokinetic parameters such as F%, which were central to their analysis. Together, these complementary studies suggest that linker methylation and its stereochemical configuration may jointly influence PROTAC conformational behavior and pharmacokinetic outcomes.

Overall, our findings demonstrate that amide-to-ester substitution, combined with the introduction of a new chiral center via methyl group incorporation, induces conformational changes that ultimately enhance the permeability of one diastereomeric PROTAC ester over the other. These results offer valuable insights into how branching and chiral centers influence PROTAC linker design and underscore the impact of molecular configuration on both permeability and degrading activity, providing a basis for future efforts to optimize PROTAC pharmacokinetics and efficacy through targeted structural modifications.

## Experimental Section

### Chemistry

#### General Methods and Materials

All chemical reagents and solvents used for synthesis were purchased from commercial suppliers and used without further purification. Reactions were performed in glassware dried in an oven at 80 °C, and their progress was monitored by thin-layer chromatography (TLC) on glass plates of silica gel 60 F254 (Merck). Preparative thin layer chromatography (pTLC) was performed on glass plates of PLC silica gel 60 F254 (Merck). Flash column chromatography was performed on a Biotage (Charlotte, NC, USA) Isolera One system equipped with Biotage SNAP cartridges or Silica gel (Kanto Chemical. Co. Inc., Silica gel 60N, 40–50 μm). HPLC was carried out on PU-980 HPLC pump (JASCO), and SSC-3315 degassing unit with a MD-2018 Plus photodiode array detector (JASCO), SSC-2120 column oven, AS-4050 HPLC autosampler (JASCO), and LC-NetII/ADC interface box (JASCO) using a C18 reverse-phase column (Inertsil ODS-4, 150 × 4.6 mm, 5 μm (GL Science Inc.)). Preparative HPLC was performed on PU-4086 semipreparative pump (*JASCO*), UV-4075 UV/vis detector (*JASCO*), LC-NetII/ADC interface box (*JASCO*), FV-4000- 06 fraction valve unit (*JASCO*), FCC fraction collector controller (*JASCO*) and CHF122SC fraction collector (*ADVANTEC*). Reaction products were dried under reduced pressure.

Nuclear magnetic resonance (NMR) spectroscopy was performed on a JEOL JNM ECA-600 spectrometer (JEOL, Tokyo, Japan). The COSY and NOESY NMR spectra were recorded at 25 °C, using a 600 MHz Bruker NEO NMR spectrometer equipped with a cryogenic probe. Chemical shifts are expressed as parts per million (ppm, δ) and referenced to solvent signals (^1^H) as internal standards: CDCl_3_ (7.26), DMSO-*d*_*6*_ (2.05) or tetramethylsilane (TMS, ^1^H 0.00 ppm). Multiplicities are reported using the following abbreviations: s: singlet, d: doublet, t: triplet, dd: double doublet, td: triple doublet, m: multiplet, br: broad, *J* coupling constants in hertz. Electrospray ionization mass spectra (ESI-MS) were obtained on a Bruker micrOTOF II mass spectrometer. All final compounds were confirmed to have a purity of >95% by HPLC analysis.

##### Synthesis of *tert*-Butyl (2-((2-(2,6-Dioxopiperidin-3-yl)-1,3-dioxoisoindolin-4- yl)­oxy)­ethyl)­carbamate (**6**)

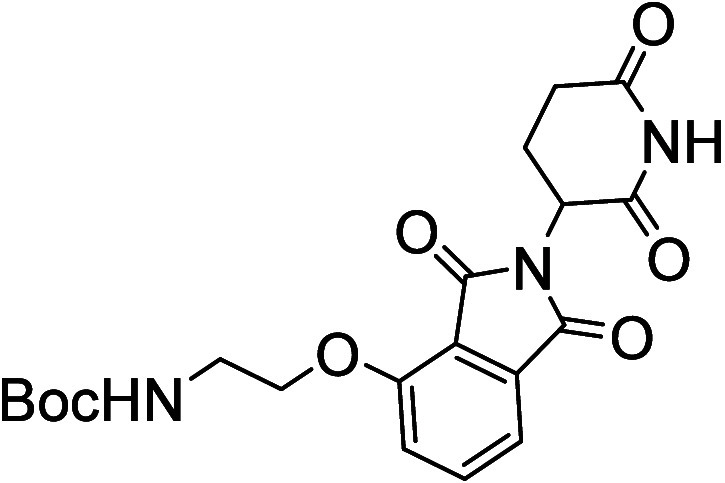



KI (85.2 mg, 513 μmol) and KHCO_3_ (37.4 mg, 374 mmol) were added to a mixture of **5** (164 mg, 733 μmol) and 4-hydroxythalidomide (201 mg, 733 μmol) in DMF (1.5 mL) under a nitrogen atmosphere. The mixture was stirred at 80 °C overnight, then the reaction was quenched with water. The organic layer was separated, and the aqueous layer was extracted three times with EtOAc. The combined organic layers were washed with water and brine, then dried over Na_2_SO_4_ and filtered. The filtrate was concentrated in vacuo, and the resulting residue was purified by column chromatography on silica gel (hexane:EtOAc = 3:1 to 1:3) to afford the product as a white powder (104.4 mg, 250.1 μmol, 34.1%). ^1^H NMR (600 MHz, CDCl_3_) δ 7.68 (dd, *J* = 8.3, 7.3 Hz, 1H), 7.61 (t, *J* = 7.8 Hz, 1H), 7.47 (d, *J* = 6.9 Hz, 1H), 7.41 (d, *J* = 6.9 Hz, 1H), 7.24 (d, *J* = 8.7 Hz, 1H), 7.20 (d, *J* = 8.7 Hz, 1H), 5.39 (s, 1H), 5.00–4.95 (m, 2H), 4.23 (t, *J* = 4.4 Hz, 2H), 4.12 (d, *J* = 7.3 Hz, 1H), 3.61 (d, *J* = 5.0 Hz, 2H), 2.90–2.76 (m, 5H), 2.15–2.11 (m, 2H), 2.05 (s, 1H), 1.44 (s, 9H), 1.26 (t, *J* = 7.1 Hz, 1H).

##### 4-(2-Aminoethoxy)-2-(2,6-dioxopiperidin-3-yl)­isoindoline-1,3-dione, HCl (**7**)

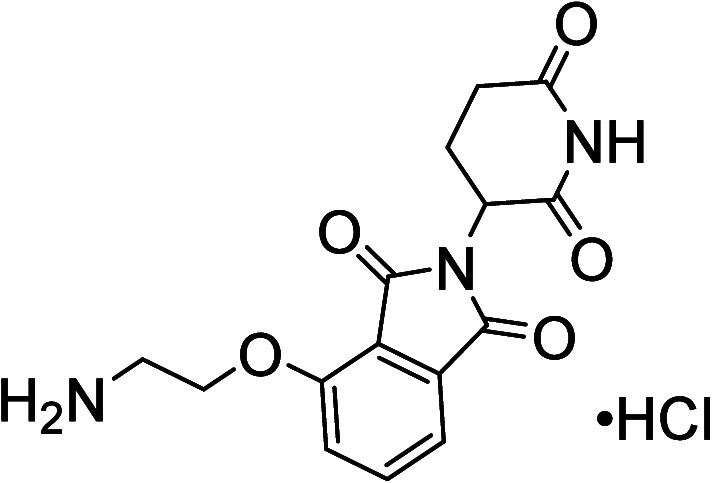



A solution of 4 M HCl in CPME (1.25 mL, 5.00 mmol) was added to solution of **6** (104 mg, 250 μmol) in DCM (2 mL) under a nitrogen atmosphere. The reaction mixture was stirred at room temperature overnight, then concentrated in vacuo to afford a crude white powder, which was used in the following step without further purification.

##### Synthesis of 4-(Benzyloxy)-*N*-(2-((2-(2,6-dioxopiperidin-3-yl)-1,3-dioxoisoindolin-4-yl)­oxy)­ethyl)­butanamide (**10**)

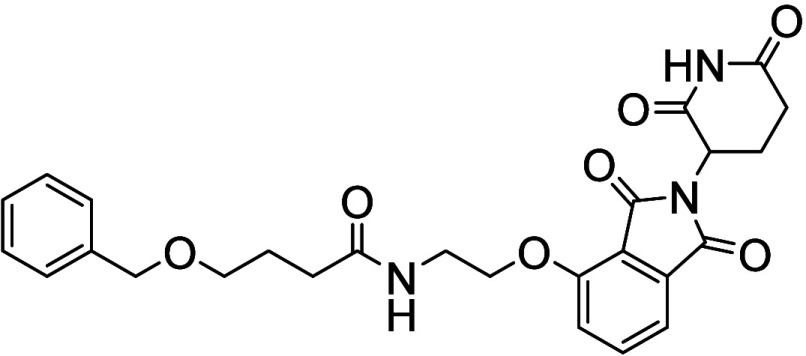



DIEA (41.7 μL, 240 μmol) and HATU (23.9 mg, 62.9 μmol) were added to a solution of **7** (27.5 mg, 77.7 μmol) and **8** (22.8 mg, 117 μmol) in THF (2 mL) under a nitrogen atmosphere. The mixture was stirred at room temperature for 5 h, then concentrated in vacuo, and the resulting residue was purified by pTLC (EtOAc) to afford **10** (16.2 mg, 32.8 μmol, 42%) as a yellow solid. ^1^H NMR (600 MHz, CDCl_3_) δ 8.68 (s, 1H), 7.68 (dd, *J* = 8.3, 7.3 Hz, 1H), 7.48 (d, *J* = 7.3 Hz, 1H), 7.32–7.29 (m, 4H), 7.27 (s, 1H), 7.26–7.22 (m, 2H), 6.72 (t, *J* = 5.5 Hz, 1H), 4.94 (q, *J* = 6.0 Hz, 1H), 4.49 (d, *J* = 18.3 Hz, 2H), 4.23–4.18 (m, 2H), 3.72–3.65 (m, 2H), 3.53–3.49 (m, 2H), 2.84–2.66 (m, 3H), 2.33 (t, *J* = 7.6 Hz, 2H), 2.09–2.06 (m, 1H), 1.96–1.92 (m, 2H). MS (ESI) *m*/*z* 440 [M + Na]^+^.

##### Sodium (*R*)-4-Hydroxypentanoate **15**

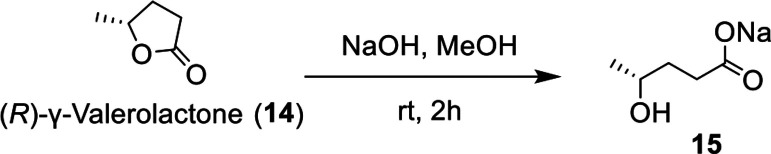



(*R*)-γ-Valerolactone (**14**) (50 mg, 0.50 mmol) was added to a solution of NaOH in MeOH (1.16 molar, 0.43 mL) and the mixture was stirred at rt for 2 h. The solvent was removed under reduced pressure to afford compound **15** as a white solid (69 mg, 0.49 mmol, 99%).

##### *N*-(2-((2-(2,6-Dioxopiperidin-3-yl)-1,3-dioxoisoindolin-4-yl)­oxy)­ethyl)-4-hydroxypentanamides **11** and **16**

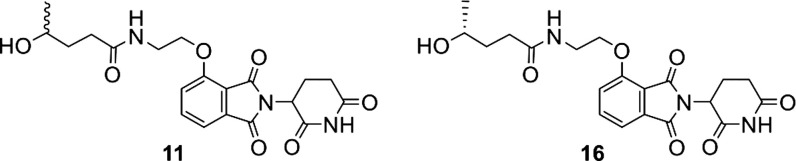



DIEA (95.4 mg, 129 μL, 738 μmol) and HATU (168 mg, 443 μmol) were added to a solution of **9** (52.2 mg, 148 μmol) or **15** (62.0 mg, 443 μmol) in DMF (1 mL) at room temperature under a nitrogen atmosphere. The mixture was stirred at the same temperature for 1.5 h, then the solvent was evaporated and the remaining residue was purified by column chromatography on silica gel eluted with 1–10% MeOH/CHCl_3_ followed by pTLC with 6% MeOH/CHCl_3_ to afford *N*-(2-((2-(2,6-dioxopiperidin-3-yl)-1,3-dioxoisoindolin-4-yl)­oxy)­ethyl)-4-hydroxypentanamide (**11**) or **16** (23 mg, 55 μmol, 37%) as a white residue. ^1^H NMR (600 MHz, MeOH-*d*_*4*_) δ 7.75 (dd, *J* = 8.5, 7.3 Hz, 1H), 7.44 (t, *J* = 7.3 Hz, 2H), 5.12 (dd, *J* = 12.8, 5.5 Hz, 1H), 4.27 (t, *J* = 5.3 Hz, 2H), 3.75 – 3.68 (m, 1H), 3.65 – 3.60 (m, 2H), 2.93 – 2.83 (m, 1H), 2.79 – 2.66 (m, 2H), 2.40 – 2.24 (m, 2H), 2.18 – 2.10 (m, 1H), 1.77 – 1.63 (m, 2H), 1.14 (d, *J* = 6.2 Hz, 3H). ^13^C NMR (151 MHz, MeOH-*d*_*4*_) δ 176.38, 174.60, 171.40, 168.44, 167.86, 157.54, 138.12, 135.01, 120.88, 118.29, 116.95, 69.28, 67.91, 50.45, 39.68, 35.78, 33.48, 32.15, 23.62, 23.42. HRMS [ESI] *m*/*z* calcd for C_20_H_23_N_3_O_7_Na [M + Na]^+^ 440.1428, found 440.1416.

##### Synthesis of 4-((2-((2-(2,6-Dioxopiperidin-3-yl)-1,3-dioxoisoindolin-4-yl)­oxy)­ethyl)­amino)-4-oxobutyl 2-((*S*)-4-(4-Chlorophenyl)-2,3,9-trimethyl-*6H*-thieno­[3,2-*f*]­[1,2,4]­triazolo­[4,3-*a*]­[1,4]­diazepin-6-yl)­acetate **1**

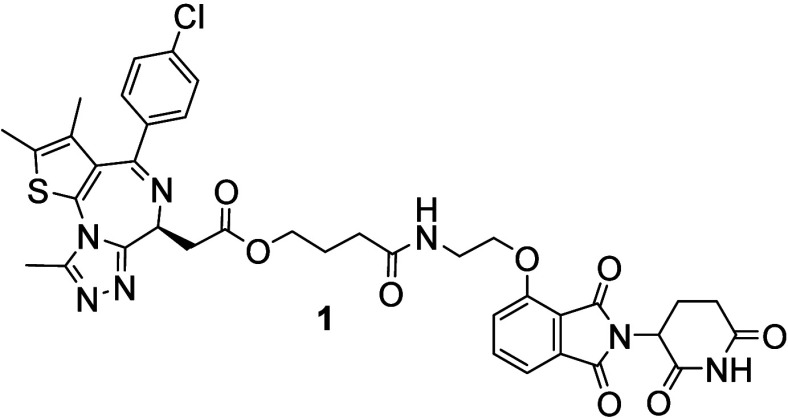



Pd/C was added to a solution of **10** (8.00 mg, 16.2 μmol) in THF (2 mL) and stirred under an H_2_ atmosphere at room temperature for 3 days. The suspension was filtered through Celite and washed with methanol. The solvent was removed under reduced pressure. The crude product was used in the following reaction without further purification.

DIEA (9.00 μL, 51.7 μmol) and HATU (10.2 mg, 27 μmol) were added to mixture of the crude product obtained in the previous step and JQ-1 carboxylic acid **12** (10.4 mg 25.9 μmol) in DCM under a nitrogen atmosphere. The mixture was stirred at room temperature overnight, then concentrated in vacuo and the resulting residue was purified by preparative pTLC (CHCl_3_/MeOH 15:1) to afford **1** (4.8 mg, 16.2 μmol, 38%) as a white solid. ^1^H NMR­(600 MHz,CDCl_3_) δ 9.07 (br s, 1H), 7.68 (ddd, *J* = 8.5, 7.3, 1.8 Hz, 1H), 7.50 – 7.47 (m, 1H), 7.40 (dd, *J* = 8.5, 2.7 Hz, 2H), 7.32 (dd, *J* = 8.8, 4.2 Hz, 2H), 7.29 – 7.27 (m, 1H), 7.16 (q, *J* = 5.7 Hz, 1H), 4.97 – 4.90 (m, 1H), 4.60 (td, *J* = 7.4, 0.9 Hz, 1H), 4.34 – 4.21 (m, 3H), 4.16 – 4.08 (m, 1H), 3.80 – 3.73 (m, 1H), 3.72 – 3.65 (m, 2H), 3.54 (dt, *J* = 16.6, 7.6 Hz, 1H), 2.87 – 2.76 (m, 2H), 2.74 – 2.66 (m, 1H), 2.65 (d, *J* = 1.7 Hz, 3H), 2.47 – 2.34 (m, 5H), 2.15 – 2.09 (m, 1H), 2.07 – 2.01 (m, 2H), 1.68 (s, 3H). ^13^C NMR (126 MHz, CDCl_3_) δ 172.90, 172.88, 171.59, 171.58, 171.17, 171.16, 168.25, 168.24, 166.92, 166.23, 166.21, 164.19, 164.16, 156.29, 156.26, 155.28, 150.06, 150.05, 136.87, 136.71, 136.54, 133.68, 133.66, 132.24, 132.21, 130.93, 130.89, 130.37, 130.34, 129.90, 129.88, 128.74, 128.73, 120.45, 120.36, 118.13, 118.08, 116.79, 116.77, 77.28, 77.03, 76.77, 68.98, 68.92, 63.86, 63.81, 53.86, 53.82, 49.32, 38.41, 38.38, 36.75, 36.72, 32.82, 32.81, 31.47, 24.85, 22.61, 22.58, 14.42, 13.12, 11.83. HRMS [ESI] *m*/*z* calcd for C_38_H_36_ClN_7_O_8_SNa [M + Na]^+^ 808.1927, found 808.1949; purity 98.9%.

##### (2*R*)-5-((2-((2-(2,6-Dioxopiperidin-3-yl)-1,3-dioxoisoindolin-4-yl)­oxy)­ethyl)­amino)-5-oxopentan-2-yl 2-((6*S*)-4-(4-Chlorophenyl)-2,3,9-trimethyl-6*H*-thieno­[3,2-*f*]­[1,2,4]­triazolo­[4,3-*a*]­[1,4]­diazepin-6-yl)­acetate **2a and 2b**

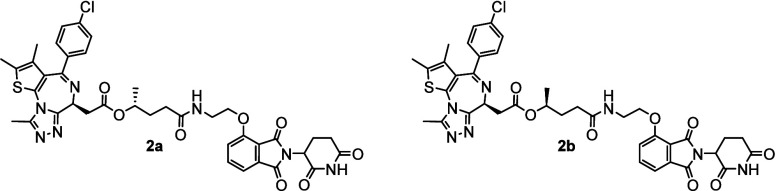



To a solution of (+)-JQ-1 carboxylic acid **12** (10.1 mg, 25.2 μmol) in DCM (0.5 mL), thionyl chloride (36.8 μL, 504 μmol) was added under a nitrogen atmosphere at room temperature. The reaction mixture was stirred at the same temperature for 1.5 h, then concentrated in vacuo, and the resulting toffee-like residue **13** was used in the following step without further purification.

To the above residue, a solution of **11** (5.00 mg, 12.0 μmol) in DCM (0.5 mL) was added followed by DIEA (3.00 μL, 17.2 μmol) at room temperature under a nitrogen atmosphere. The mixture was stirred at the same temperature overnight, then concentrated in vacuo and the residue was purified by reverse-phase HPLC (InertSustain C18, 5 μm, 250 × 20 mm, 5% MeOH in 0.1% aq. FA to 50% over 30 min) to afford **2a** (2 mg, 2 μmol, 20%) as a white powder and 2**b** (2 mg, 2 μmol, 20%) as a white powder. **2a**: ^1^H NMR (600 MHz, MeOH-*d*_*4*_) δ 8.34 (br s, 1H), 7.76 – 7.70 (m, 1H), 7.45 – 7.37 (m, 6H), 7.28 – 7.03 (m, 1H), 5.11 – 5.04 (m, 1H), 5.02 – 4.96 (m, 1H), 4.60 – 4.54 (m, 1H), 4.32 – 4.25 (m, 2H), 3.70 – 3.57 (m, 2H), 3.54 – 3.43 (m, 2H), 2.88 – 2.78 (m, 1H), 2.75 – 2.63 (m, 5H), 2.44 (s, 3H), 2.29 (t, *J* = 7.6 Hz, 2H), 2.14 – 2.07 (m, 1H), 1.96 – 1.83 (m, 2H), 1.69 – 1.66 (m, 3H), 1.24 (dd, *J* = 6.3, 2.1 Hz, 3H). ^13^C NMR (151 MHz, MeOH-*d*_*4*_) δ 174.17, 173.13, 170.69, 169.90, 167.03, 164.86, 156.18, 155.32, 150.82, 136.65, 136.58, 133.67, 132.16, 130.56, 130.50, 129.90, 128.44, 119.68, 117.11, 115.58, 70.71, 70.67, 67.98, 53.52, 49.07, 48.44, 48.16, 38.32, 36.27, 31.59, 31.37, 30.74, 22.26, 18.80, 13.00, 11.53, 10.19. HRMS [ESI] *m*/*z* calcd for C_39_H_38_ClN_7_O_8_SNa [M + Na]^+^ 822.2083, found 822.2091; purity 98.5%.

**2b**: ^1^H NMR (600 MHz, MeOH-*d*_*4*_) δ 8.37 – 8.22 (m, 1H), 7.72 – 7.65 (m, 1H), 7.47 – 7.35 (m, 6H), 5.07 – 4.95 (m, 2H), 4.43 (t, *J* = 7.3 Hz, 0.5H *epimer*), 4.36 (dd, *J* = 6.1, 5.8 Hz, 0.5H *epimer*), 4.27 – 4.17 (m, *J* = 6.1, 5.4 Hz, 2H), 3.77 – 3.69 (m, 1H), 3.56 – 3.37 (m, 3H), 2.90 – 2.77 (m, 1H), 2.76 – 2.63 (m, 5H), 2.44 (s, 3H), 2.39 – 2.28 (m, 2H), 2.20 – 2.08 (m, 1H), 1.99 – 1.82 (m, 2H), 1.67 (s, 3H), 1.27 (dd, *J* = 6.1, 5.3 Hz, 3H). ^13^C NMR (151 MHz, MeOH-*d*_*4*_) δ 174.10, 174.00, 173.17, 173.15, 170.87, 170.83, 169.89, 169.86, 166.99, 166.93, 164.86, 156.17, 155.23, 155.17, 150.87, 150.82, 136.62, 136.61, 136.58, 133.54, 133.47, 132.17, 132.11, 131.86, 130.57, 130.36, 129.88, 129.86, 128.42, 119.72, 117.04, 115.50, 70.41, 70.32, 68.21, 68.14, 53.54, 53.48, 49.11, 49.09, 48.17, 38.25, 38.16, 36.28, 31.49, 31.46, 31.30, 31.24, 30.77, 22.25, 22.18, 19.05, 19.00, 13.02, 11.54, 10.24. HRMS [ESI] *m*/*z* calcd for C_39_H_38_ClN_7_O_8_SNa [M + Na]^+^ 822.2083, found 822.2075; purity 97.9%.

The same reaction was reconducted using compound **16** to afford compound **2a** as the sole product.

### Biology

#### Cell Culture

MCF-7 cells and HCT-116 cells expressing HiBiT-BRD4 were cultured in Dulbecco’s modified Eagle’s medium (DMEM) supplemented with 10% heat-inactivated fetal bovine serum (FBS) and penicillin/streptomycin at 37 °C under a humidified atmosphere of 5% CO_2_ in air.

#### Compound Treatment

Compounds were prepared as stock solutions in DMSO and diluted 1,000-fold in the culture medium to a final DMSO concentration of 0.1%. Compound treatment was performed by either adding the compound to the medium or replacing the medium entirely.

#### Western Blot

Cells were lysed using RIPA buffer (Nacalai Tesque). Lysates were centrifuged at 13,500 × g for 5 min at 4 °C, and the supernatant was collected. Protein concentrations were determined using the bicinchoninic acid (BCA) protein assay, and total protein levels were normalized. Protein samples were mixed with 5× Laemmli buffer (0.25% bromophenol blue (BPB), 0.5 M dithiothreitol (DTT), 50% glycerol, 10% SDS, and 0.25 M Tris-HCl, pH 6.8), heated at 95 °C for 5 min, and cooled on ice. Proteins were separated by sodium dodecyl sulfate-polyacrylamide gel electrophoresis (SDS-PAGE) using SuperSep Ace 10–20% or 5–12% gels (198–15041/192–15201, Wako Pure Chemical Industries) or 4–20% or 7.5% Mini-PROTEAN TGX Precast Protein Gels, 15-well (4561026, Bio-Rad). Separated proteins were transferred onto PVDF membranes (Bio-Rad). The membranes were blocked at room temperature for 20 min using Bullet Blocking One (Nacalai Tesque), then incubated with the primary antibody (anti-BRD4 E2A7X, cat No. 3698S, Cell Signaling Technology, 1:1000) diluted in Can Get Signal Immunoreaction Enhancer Solution 1 (NKB-101, *TOYOBO*). The membranes were rinsed with Milli-Q five times and washed with TBS-T (5 min). Secondary antibodies (antirabbit HRP conjugate, 1:5000; antitubulin nFAB rhodamine conjugate, cat. No. 12004164, Bio-Rad, 1:2000) were diluted in Can Get Signal Immunoreaction Enhancer Solution 2 (NKB-101, Toyobo) and applied to the membranes. After incubation, the membranes were rinsed with Milli-Q five times and washed with TBS-T (5 min). To detect BRD4 signals, the bands were visualized using ImmunoStar LD (292–69903, Wako Pure Chemical Industries). Chemiluminescence signals for BRD4 and rhodamine fluorescence signals for tubulin were captured using Chemi-Doc Touch MP (Bio-Rad).

#### HiBiT Assay

A HCT116 cell line in which a HiBiT-tag was knocked-in at the N-terminus of endogenous BRD4 locus was previously described (ref: Kaiho-Soma et al., Mol Cell 2021).^40^ HiBiT assay was performed using the Nano-Glo HiBiT Lytic Detection System (Cat. No. N3030, Promega) according to the manufacturer’s protocol. The chemiluminescence intensity was measured using EnVision (PerkinElmer) and normalized by the cell viability assessed with CCK-8 (Cat. No. CK04, Dojindo) at 1 h prior to HiBiT assay to calculate relative luminescence unit (RLU).

#### Solubility Assay

The solubility test was performed using Japanese Pharmacopoeia (JP) first test fluid (pH 1.2) and JP second test fluid (pH 6.8). Solutions of the compounds were prepared by diluting 10 mM DMSO stock solution 2 μL:165 μL in JP first or second fluid and mixed at 37 °C for 4 h by rotation at 1000 rpm. The mixed solution was loaded into 96-well MultiScreen Filter Plates (product number MSHVN4510, 0.45 μm hydrophilic PVDF membrane; Millipore, Bedford, MA), and filtration was performed by centrifugation. The obtained filtrates were analyzed by HPLC with UV detection at 254 nm. The solubility was determined by comparing the peak area of the filtrate with that of a 100 μM standard solution. When the peak area of the filtrate was larger than that of the standard solution, it was described as >100 μM. Two technical replicates were performed.

#### PAMPA Assay to Determine the Passive Membrane Diffusion Rates

A Corning Gentest Precoated PAMPA Plate System was used in the PAMPA permeability test. The acceptor plate was prepared by adding 200 μL of 0.1 M phosphate buffer (pH 7.4) supplemented with 5% DMSO to each well, and then 300 μL of 10 μM compounds in 0.1 M phosphate buffer (pH 6.4) with 5% DMSO was added to the donor wells. The acceptor plate was then placed on top of the donor plate and incubated at 37 °C for 4 h without agitation. After the incubation, the plates were separated and the solutions from each well of both the acceptor plate and the donor plate were transferred to 96-well plates and mixed with acetonitrile. The final concentrations of compounds in both the donor wells and acceptor wells, as well as the concentrations of the initial donor solutions, were analyzed using liquid chromatography-tandem mass spectrometry (LC-MS/MS). The permeability of the compounds was calculated as described in a previous study.^41^ Antipyrine (100 μM, 100% gastrointestinal absorption in humans), metoprolol (500 μM, 95% absorption) and sulfasalazine (500 μM, 13% absorption) were used as reference compounds. The permeabilities of antipyrine, metoprolol and sulfasalazine were 19.5, 1.66, and 0.072 × 10^–6^ cm/s, respectively. Four technical replicates were performed.

#### Hepatic Microsomal Stability Assay

Disappearance of the parent compound over time was measured by using the amount of drug at time zero as a reference. After 5 min of preincubation, 1 mM NADPH (final concentration; the same applies to the following) was added to a mixture containing 1 μM of the compound, 0.2 mg/mL of mouse liver microsomes (Sekisui XenoTech LLC, Kansas City, KS), 1 mM EDTA and 0.1 M phosphate buffer (pH 7.4). The mixture solution was incubated at 37 °C for 30 min with rotation at 60 rpm. An aliquot of 50 μL of the incubation mixture was added to 250 μL of chilled acetonitrile/internal standard (IS, Methyltestosterone). After centrifugation at 3150 × g for 15 min at 4 °C, the supernatants were analyzed by LC-MS/MS. Hepatic microsomal stability (mL/min/kg, CLint) was calculated according to a previous report,^42^ using 45.4 mg MS protein/g liver and 87.5 g liver/kg body weight as scaling factors. Two technical replicates were performed.

#### Stability in Media

Disappearance of the parent compound over time was measured by using the amount of drug at time zero as a reference. The mixture solution of 1 μM of the compound and cell media was incubated at 37 °C for 6 h. An aliquot of 50 μL of the incubation mixture was added to 200 μL of chilled acetonitrile/IS. After centrifugation at 10000 × g for 5 min at 4 °C, the supernatants were analyzed by LC-MS/MS. Two technical replicates were performed.

#### LC-MS/MS Quantification Method

We utilized an LC-MS8060 instrument equipped with a Shimadzu Nexera series LC system (Shimadzu, Kyoto, Japan) for our analysis. All compounds were analyzed in multireaction monitoring mode under electron spray ionization conditions. The analytical column employed was a CAPCELLPAK C18 MGIII (3 μm × 2.0 mm ID × 35 mm; OSAKA SODA, Osaka, Japan) maintained at 50 °C. The gradient mobile phase consisted of 0.1% formic acid in water (mobile phase A) and 0.1% formic acid in acetonitrile (mobile phase B) at a total flow rate of 1 mL/min. Initially, the mobile phase composition was 10% B and was held constant for 0.5 min. It was then linearly increased to 90% B over 1 min, followed by a constant hold for 0.8 min. The mobile phase was then returned to the initial condition of 10% B over 0.01 min and re-equilibrated for 1 min. The transitions (precursor ion > product ion) of dBET1, 1, 2a, 2b and IS (methyltestosterone) were 785.2 > 383.0, 786.2 > 386.1, 798.2 > 273.2, 798.2 > 273.2 and 303.1 > 109.1 (positive), respectively.

#### Compound Uptake Evaluation

The culture medium was prepared with dBET1, as well as ester PROTACs, at a final concentration of 10 μM. The evaluations were performed in triplicate. MCF-7 cells were treated with compounds for the designated time periods (0, 30 min, 6 h). Following incubation, a portion of the medium was collected, while the remaining medium was removed, and cells were washed sequentially with ice-cold medium and ice-cold PBS. Cells were then resuspended in 100 μL of PBS and collected using a cell scraper. For 0-h samples, the compound-containing medium was removed immediately after treatment, and cells were washed and collected as described above. Cells were lysed, and acetonitrile was added to the lysates. Each mixture was vortexed, centrifuged, and the supernatant was collected. The compound concentration in the supernatant was determined by LC-MS/MS. The intracellular concentration was calculated based on the measured compound concentration, the number of cells collected, and the estimated volume per cell.

### Computational Methods

#### Steered Molecular Dynamics Simulation

The 3D structures of both diastereomeric PROTACs were generated and subjected to energy minimization using Maestro (Maestro, Schrödinger, New York, USA, 2021). The stereochemistry of the thalidomide chiral center was set to the *R*-configuration. The 3D structures were further processed using the ″Ligand Reader and Modeler″ tool available in the online CHARMM-GUI input generator (www.charmm-gui.org). Molecular dynamics (MD) input files, including equilibration and production parameters, were subsequently generated using the CHARMM36m force field. To solvate the PROTACs with water, the ″Solution Builder″ module in CHARMM-GUI was used to construct a periodic water box around the molecules. Simulations were conducted under isothermal–isobaric (NPT) conditions at 300 K. Additionally, the ″Multicomponent Assembler″ tool was employed to generate periodic simulation boxes for toluene and a 1:1 DMSO-water mixture.

Equilibration (0.25 ns) and production (20 ns) simulations were performed using the CUDA-accelerated NAMD3 (www.ks.uiuc.edu/Research/namd/). Steered molecular dynamics (SMD) simulations were incorporated by modifying the production input file with the following parameters: SMD = on, SMDk = 7.0 kcal/mol/Å, SMDvel = 2 × 10^–5^ Å/ts, and SMDdir = (0.0, 1.0, 0.0). Furthermore, the occupancy factor in the input PDB files was adjusted by setting the value to 1 for PROTAC atoms and 0 for solvent atoms to ensure that SMD forces were applied only to the PROTAC molecules.

ChimeraX (https://www.cgl.ucsf.edu/chimerax/) and VMD (http://www.ks.uiuc.edu/Research/vmd/) were used for visualization, and solvent removal from the production trajectories.

The processed trajectories were then saved for further analysis using VMD (http://www.ks.uiuc.edu/Research/vmd/).

#### Calculations of Molecular Properties

The formation of IMHBs was explored with VMD using the H-Bond plugin. The standard criteria for hydrogen bond formation were applied, considering hydrogen atoms bound to nitrogen or oxygen as donors and nitrogen, oxygen, or sulfur atoms with lone pairs as acceptors. A relaxation threshold of 4 Å for bond distance and 20° for the angle between the hydrogen bond donor (HBD) and hydrogen bond acceptor (HBA) was permitted.

The SA 3D PSA (calculated using a solvent probe radius of 1.4 Å) and *R*_*gyr*_ were calculated using PyMOL and MDTraj,^43^ respectively. The ensemble similarity searches were conducted using an RMSD-based method calculated with MDTraj.

#### Clustering of Simulation Trajectories

The MD trajectory of each simulation was analyzed using MDTraj. All heavy atoms were selected for structural comparisons, and pairwise RMSD values between frames were computed to generate an RMSD matrix. K-means clustering (n = 10) was applied using Python to partition the conformational ensemble. For each cluster, the frame closest to the cluster centroid was identified as the representative conformation.

#### iMTD-GC and LowModeMD Conformational Searches

Both diastereomers were subjected to conformational searches using the GFN2-xTB method, as implemented in the CREST software,^36^ with the ALPB implicit solvation model applied for both water and toluene. Subsequently, additional conformational searches were performed using the LowModeMD method implemented in MOE (Molecular Operating Environment (MOE), 2022 Chemical Computing Group ULC, 910–1010 Sherbrooke St. W., Montreal, QC H3A 2R7) with the Amber:EHT force field and the R-field implicit solvation model for the same solvents. The resulting lowest-energy conformers from each solvent for each compound were then analyzed using RMSD-based similarity searches with MDTraj.^43^

#### Graphical and Data Analysis

Data was collected in .csv files, and all statistical analyses and visualizations were performed using Python libraries in the Jupyter Notebook environment.

## Supplementary Material





## Notes

Hill coefficient calculations; BRD4 degradation activity data for dBET1 and its ester analogs; HibiT-based quantitative analyses of compounds-induced degradation; intracellular concentration measurements of the compounds; molecular docking studies; representative conformations of **2a** and **2b** in different solvents; comparative analyses of the conformational ensembles of **2a** and **2b** in water, toluene, and DMSO–water mixtures; ^1^H and ^13^C NMR spectra and HRMS data of the synthesized compounds; COSY and NOESY spectra; HPLC purity traces for compounds **1**, **2a**, and **2b**; and *A*_NMR_ calculation data (PDF)
Molecular formula strings (SMILES codes); aqueous solubility data; PAMPA permeability measurements; and DC_50_ values for the studied compounds (CSV)

## ABBREVIATIONS

bRo5: beyond Rule of 5
IMHBs: intramolecular hydrogen bonds
iMTD-GC: iterative metadynamics and genetic crossing
MOE: molecular operating environment
PAMPA assay: parallel artificial membrane permeability assay
PSA: polar surface area
*R*_gyr_: radius of gyration
SA 3D PSA: solvent-accessible 3D polar surface area
SMD: steered molecular dynamics
NOESY: nuclear Overhauser effect spectroscopy
*A*_NMR_: hydrogen-bond acidity descriptor
