# Supplementary material for: Diastereomeric Branched-Ester dBET1 Analogs Exhibit Conformation-Dependent Differences in Passive Membrane Permeability
Source: J Med Chem. 2026 Jan 20;69(3):2900–15. doi: 10.1021/acs.jmedchem.5c02791 (PMC12910643; doi:10.1021/acs.jmedchem.5c02791)
Supplement: Supplementary file 1 [file jm5c02791_si_001.pdf]

Supporting Information

Diastereomeric Branched-Ester dBET1 Analogs Exhibit Conformation-Dependent Differences in Passive Membrane Permeability

Mazin A. S. Abdelwahid,[1,†] Eisuke Hayakawa,[1,†]Keigo Hirai,[1] Mayumi Ishii,[2] Kayoko Kanamitsu,[2] Saori Yasuda,[1] Fumiaki Ohtake,[3,4] Shinichi Sato,[1,5] Shusuke Tomoshige,[1,*] Minoru Ishikawa[1,*]

[1] Graduate School of Life Sciences, Tohoku University, 2-1-1 Katahira, Aoba-ku, Sendai 980-8577, Japan

[2] Drug Discovery Initiative, The University of Tokyo, 7-3-1 Hongo, Bunkyo-ku, Tokyo 113-0033, Japan

[3] School of Pharmacy and Pharmaceutical Sciences, Hoshi University, 2-4-41 Ebara, Shinagawa-Ku, Tokyo 142-8501, Japan.

[4] Institute for Advanced Life Sciences, Hoshi University, 2-4-41 Ebara, Shinagawa-Ku, Tokyo 142-8501, Japan.

[5] Frontier Research Institute for Interdisciplinary Sciences, Tohoku University, 6-3 Aramaki aza-Aoba, Aoba-ku, Sendai, 980-8577, Japan

† These authors contributed equally to this work

Corresponding Authors

Shusuke Tomoshige: stomoshi@tohoku.ac.jp

Minoru Ishikawa: minoru.ishikawa.e4@tohoku.ac.jp

Table of Contents

Supplementary tables S2

Supplementary Figures S3-S13

HPLC Spectra + NMR spectra + HRMS spectra S13-S52

Western blot images...S53-S54

Supplementary Table

Table S1: Hill coefficients calculated from dose-response curves fitted across the concentration range (1000, 3000, 10000 nM) exhibiting the hook effect.

Cmpd	dBET1	1	2a	2b
Hill coefficient	0.801	NC	2.73	2.87

NC, not calculated due to the difficulty of appropriate curve fitting.

Supplementary Figures

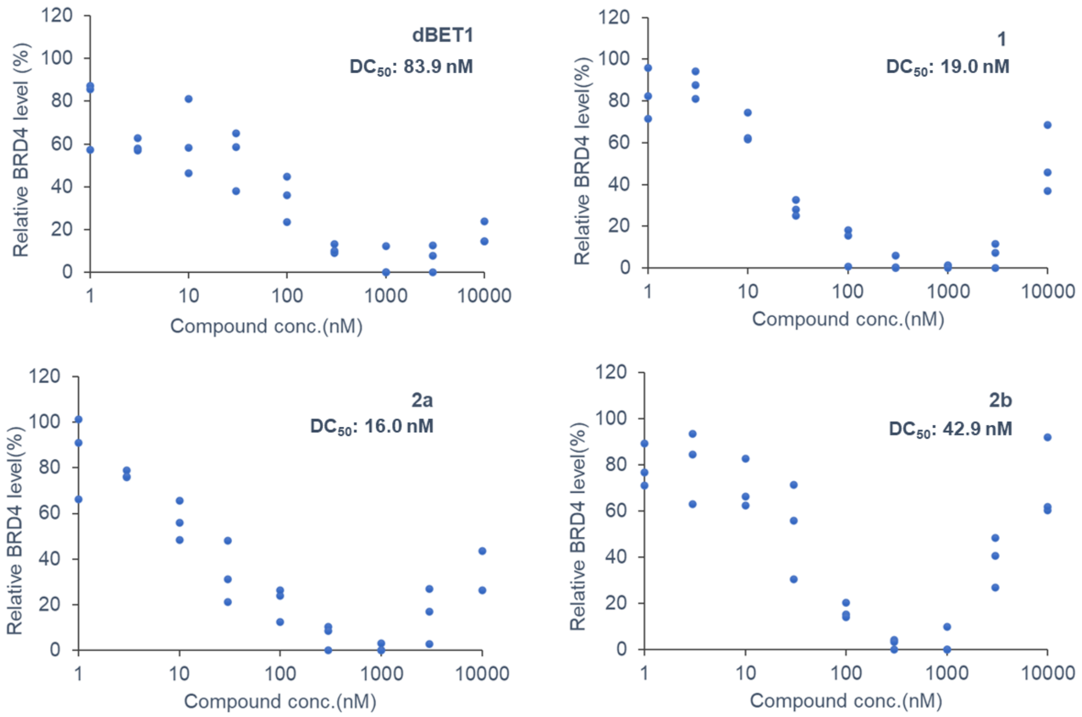

Figure S1: BRD4 degradation-inducing activity of dBET1 and its ester analogs. MCF-7 cells were treated with each compound at 10, 30, 100, 300, 1000, 3000, and 10000 nM for 4 hours, followed by western blot analysis of BRD4 abundance in the cells. Plots show data from three independent experiments, which are normalized to means of DMSO control.

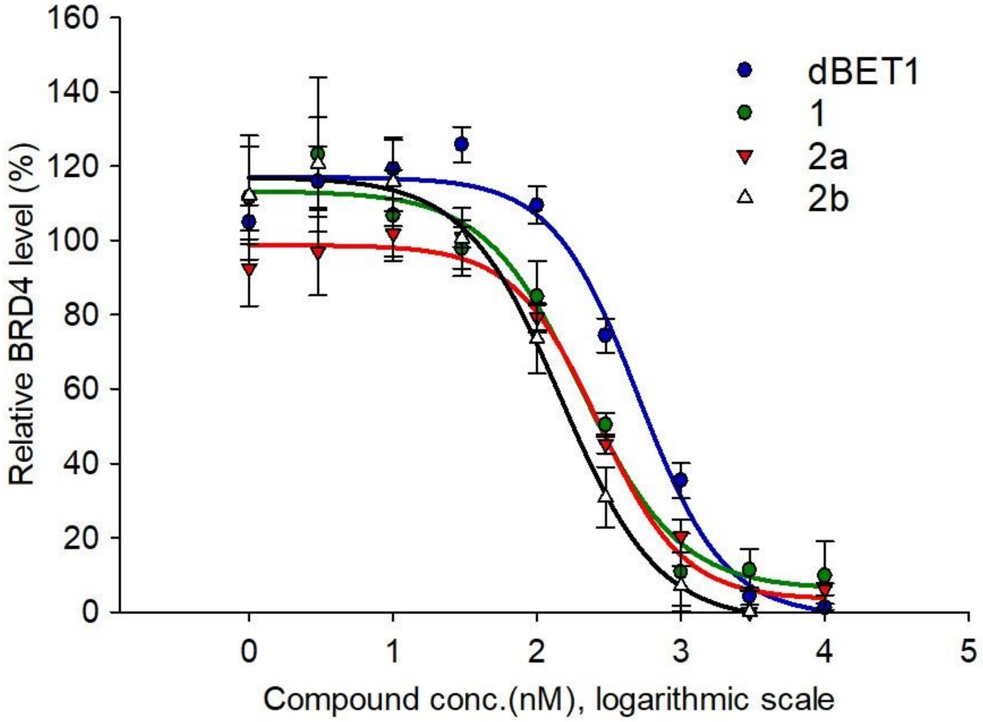

Cmpd	dBET1	**1**	**2a**	**2b**
DC_{50} ± SE (nM)	510 ± 1.2	210 ± 1.4	270 ± 1.2	150 ± 1.3

Figure S2: HiBiT-based analysis of BRD4 degradation-inducing activity of dBET1 and its ester analogs. HCT-116 cells expressing HiBiT-BRD4 were treated with each compound at 10, 30, 100, 300, 1000, 3000, and 10000 nM for 4 hours, followed by Luc assay in the cells. Plots show the mean ± SE of three independent experiments, which are normalized to means of DMSO control. The data points at high-concentration range showing the hook effect were removed for graph visibility.

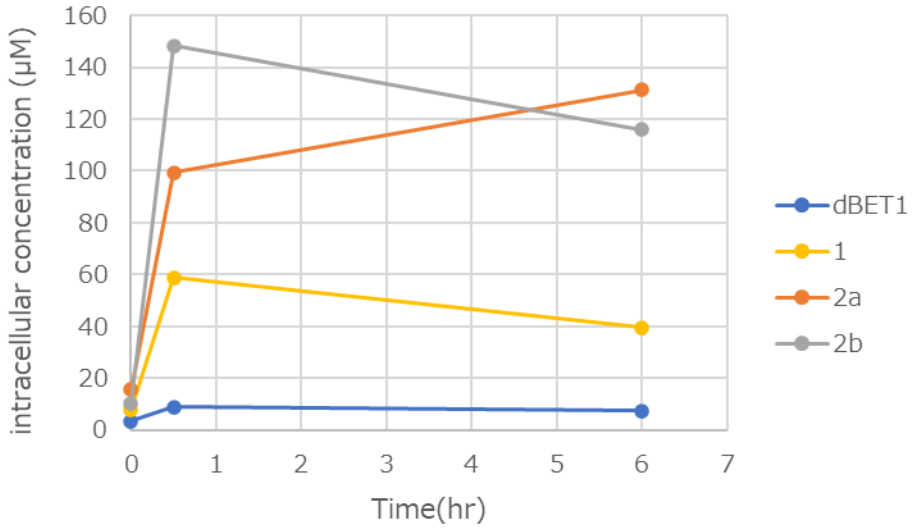

Figure S3: Intracellular concentration of PROTACs. HeLa cells were treated with each PROTAC for the indicated times. Subsequently, cells were harvested, and the intracellular concentrations of PROTACs were determined by LC-MS/MS.

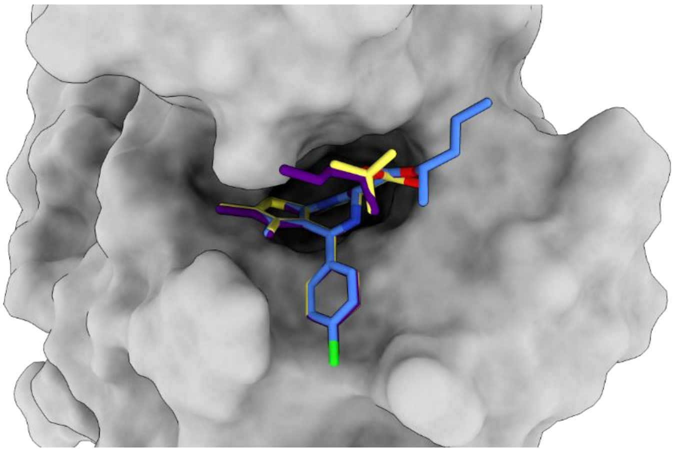

Figure S4: Docking poses of the JQ1 sec-amyl ester enantiomers (cornflower blue, MOE S-score = –8.145; purple, S-score = –8.225) overlaid with the co-crystallized JQ1 ligand (yellow).

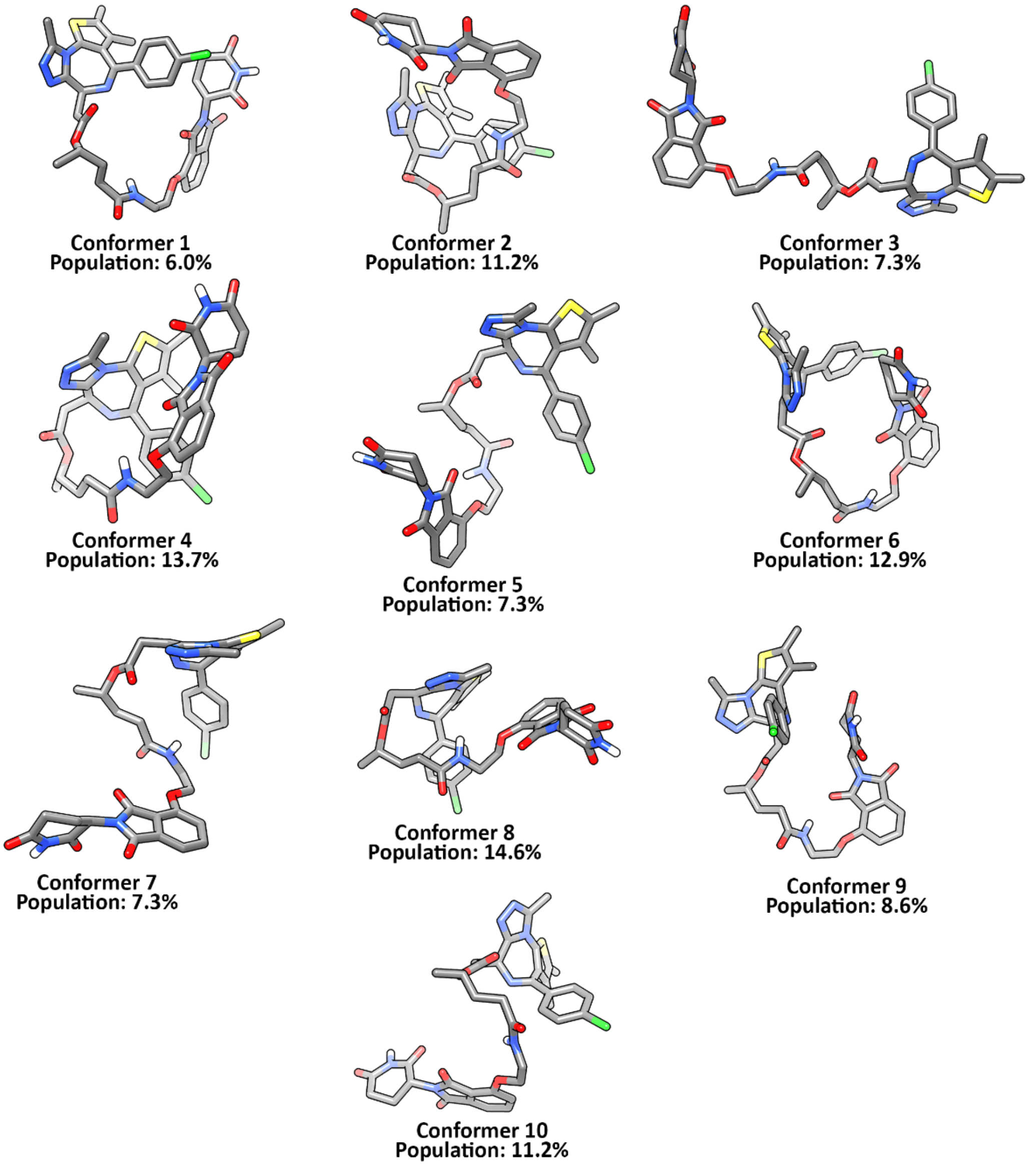

Figure S5: Representative conformations of the compound **2b** ensemble clustering in water

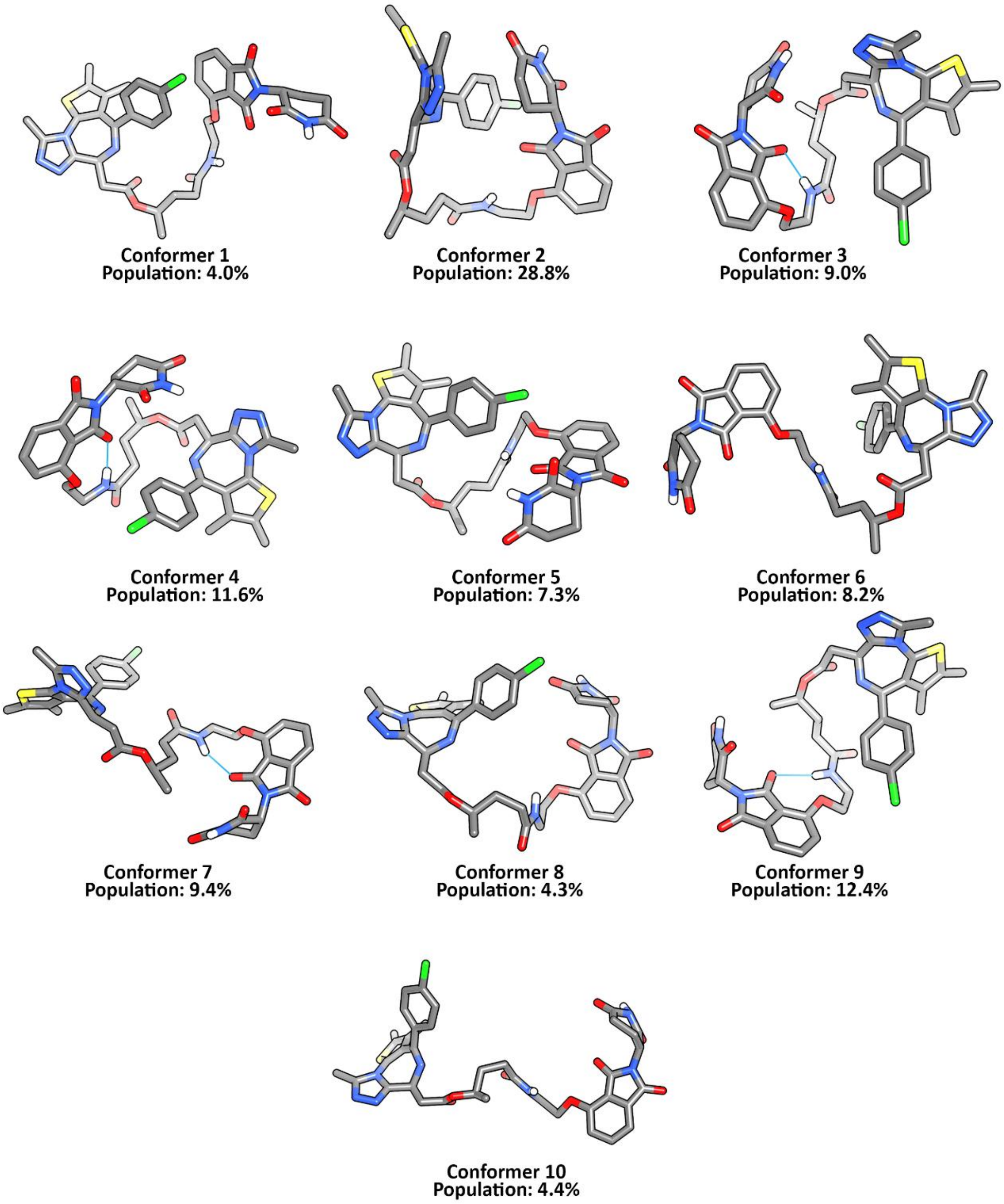

Figure S6: Representative conformations of the compound **2a** ensemble clustering in water

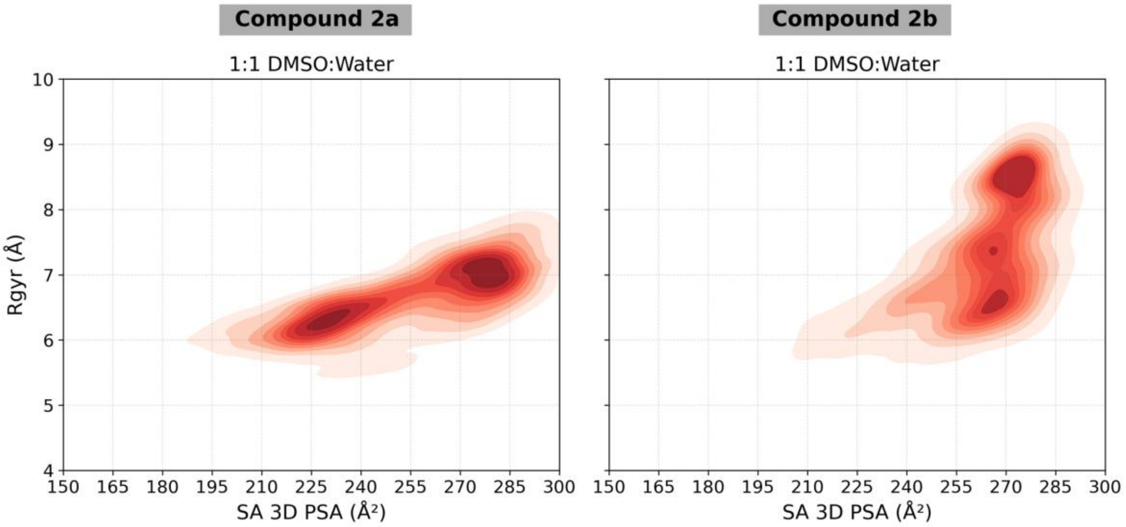

Figure S7: Comparative SMD analysis of the conformational ensembles of 2a and 2b in 1:1 DMSO-water mixture. The plots show the relationship between R_{gyr} and 3D PSA. Data is shown for **2a** (left column) and **2b** (right column) simulated in 1:1 DMSO-water mixture. Darker red regions indicate a higher population density of conformations.

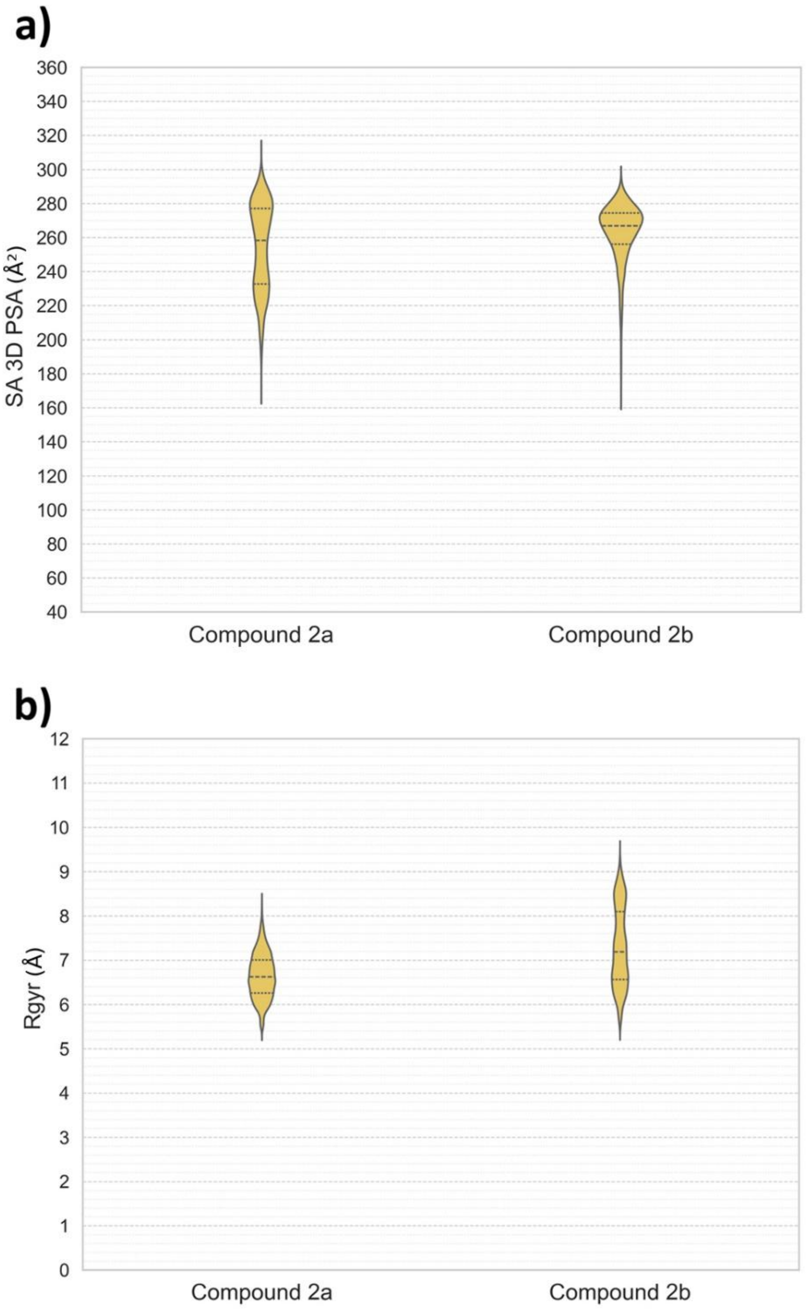

Figure S8: Violin plots of the SA 3D PSA distribution of **2a** and **2b** in 1:1 DMSO-water (**a**), and the R_{gyr} distribution of **2a** and **2b** in 1:1 DMSO-water (**b**). Dashed lines inside the violins indicate the quartiles.

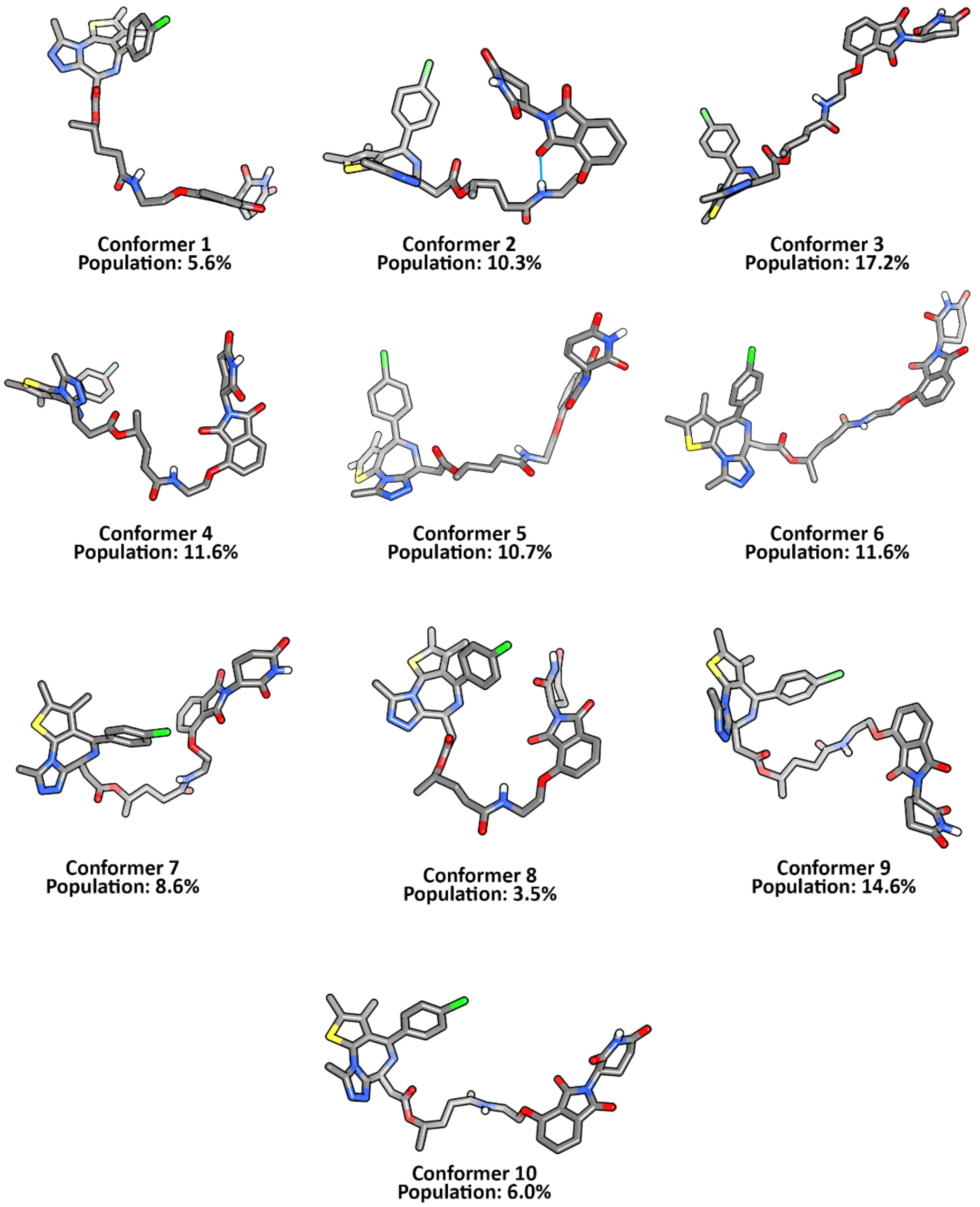

Figure S9: Representative conformations of compound **2b** ensemble clustering in DMSO-water

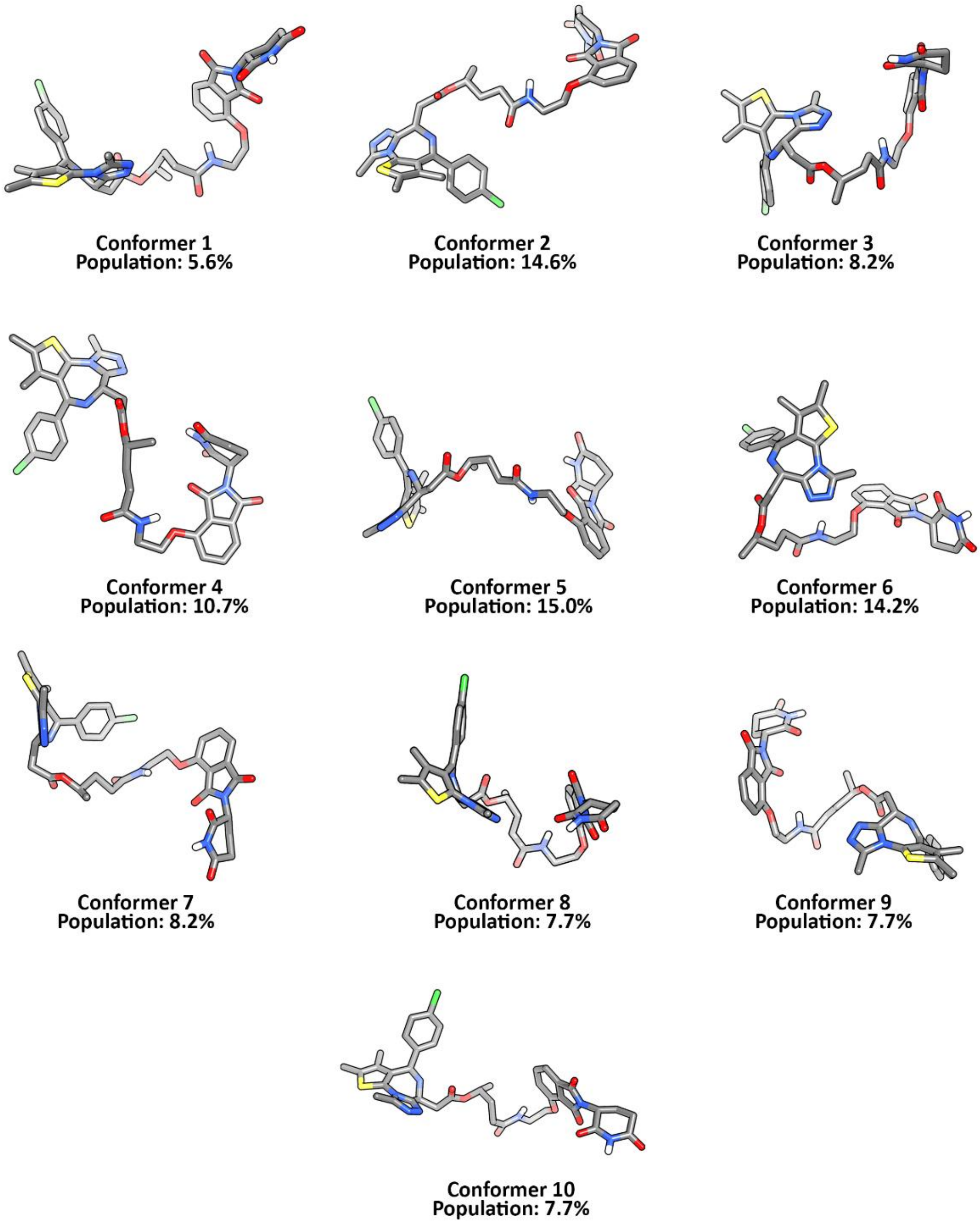

Figure S10: Representative conformations of compound **2a** ensemble clustering in DMSO-water

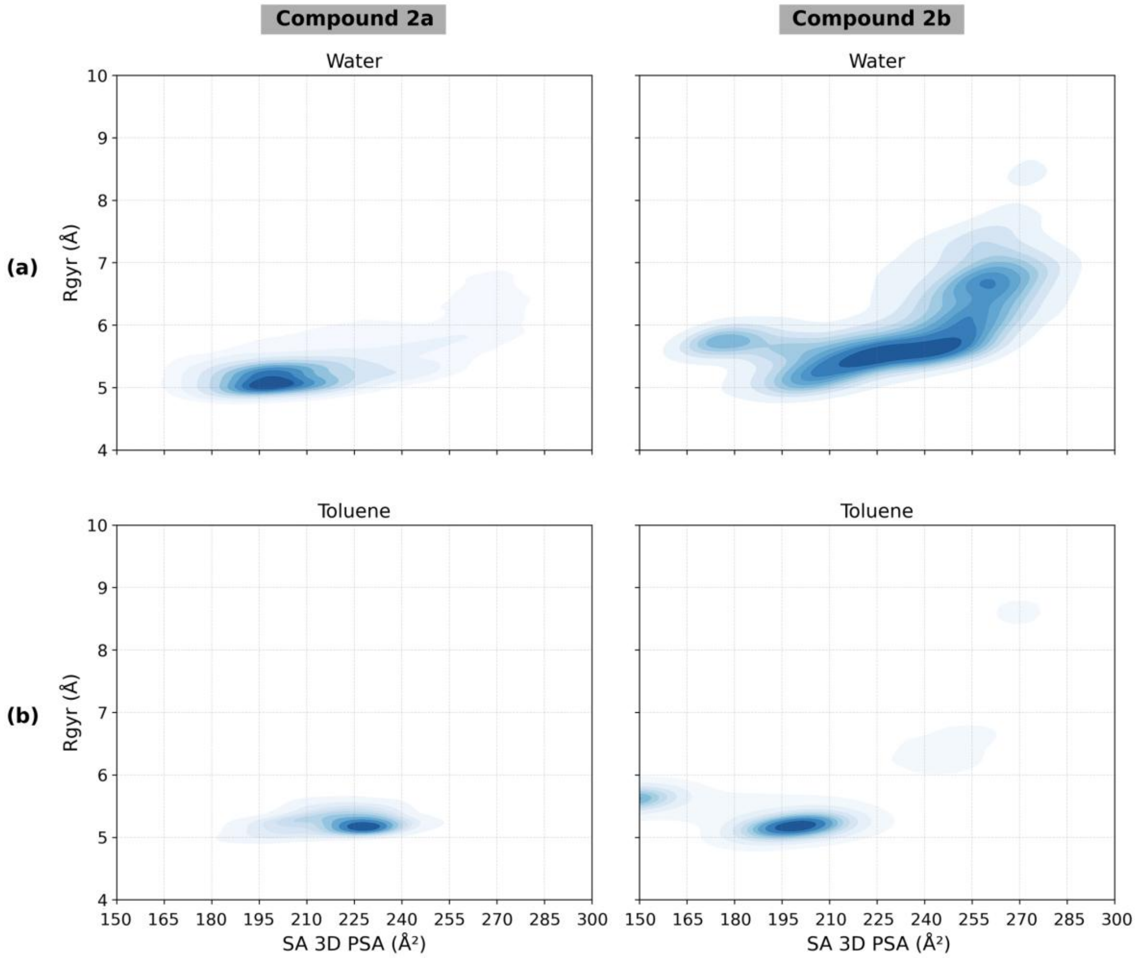

Figure S11: A comparative analysis of the conformational ensembles of **2a** and **2b** generated by the second run of SMD. The plots show the relationship between R_{gyr} and SA 3D PSA. Data is shown for **2a** (left column) and **2b** (right column) simulated in three different solvent environments: water (**a**) and toluene (**b**). Darker blue regions indicate a higher population density of conformations.

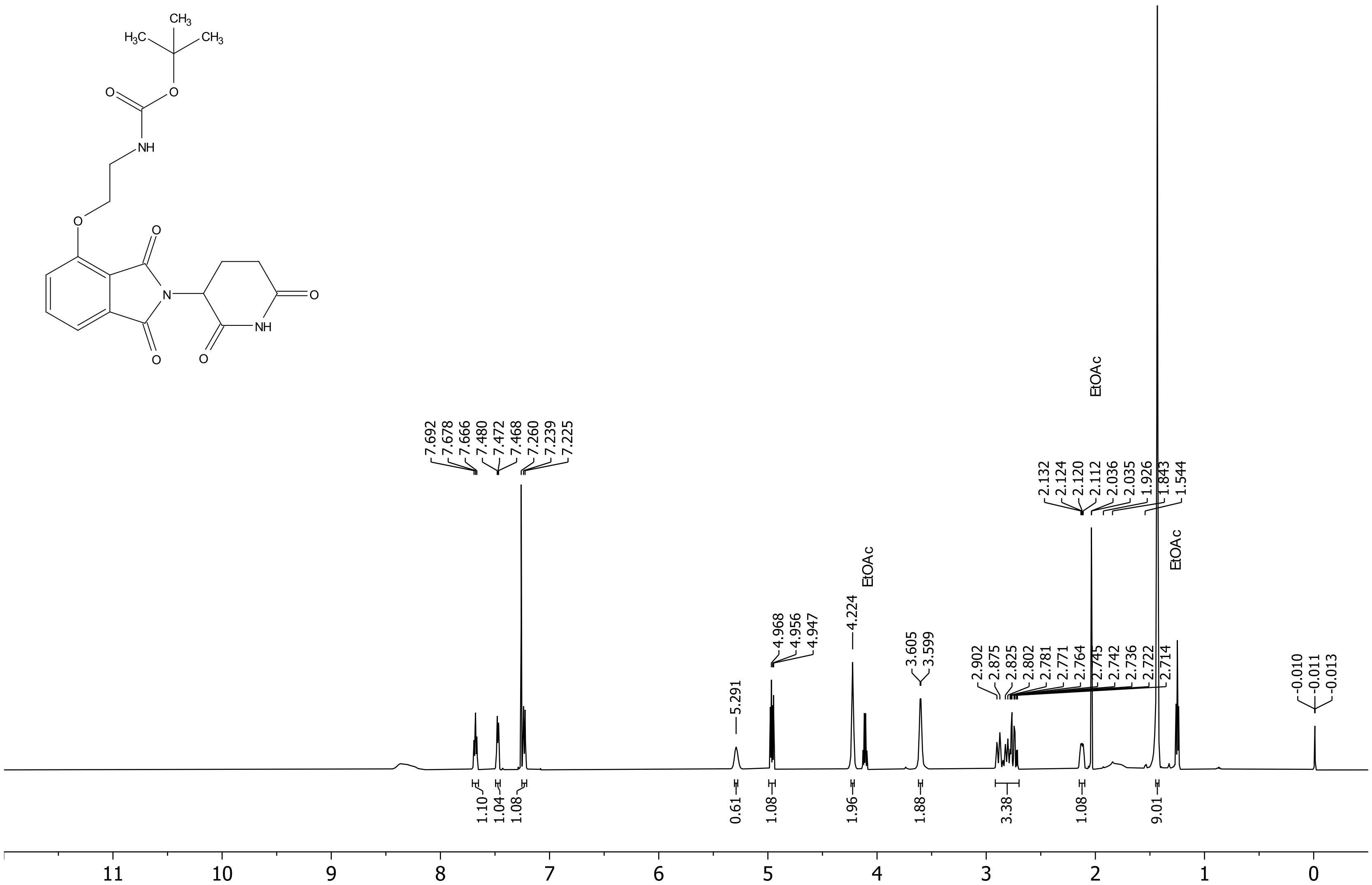

Figure S12: ^{1}H NMR (600 MHz, $CDCl_3$) of Compound **6**

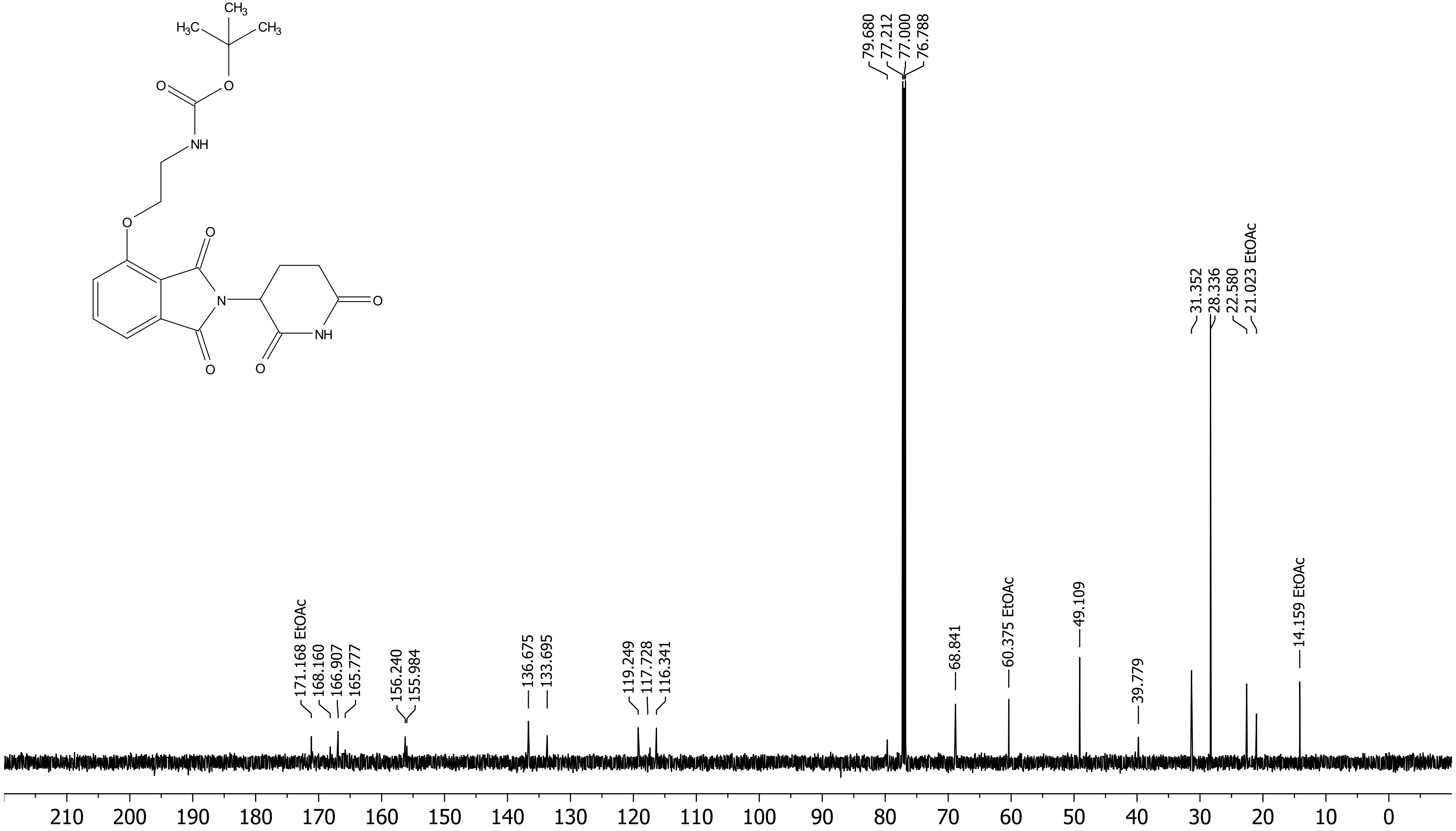

Figure S13: ^{13}C NMR (150 MHz, $CDCl_3$) of Compound **6**

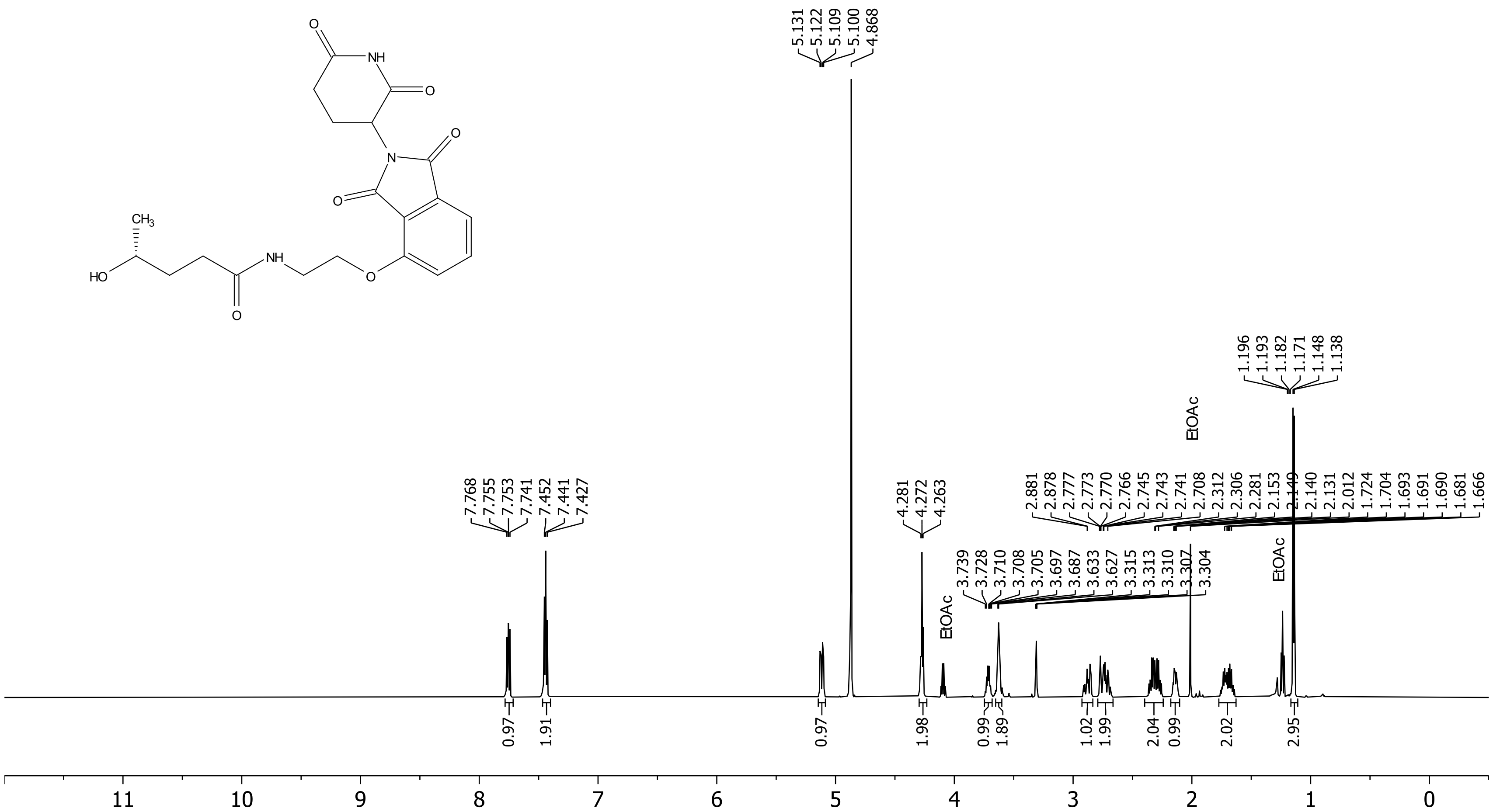

Figure S14: ^{1}H NMR (600 MHz, MeOH-d_4) of Compound **16**

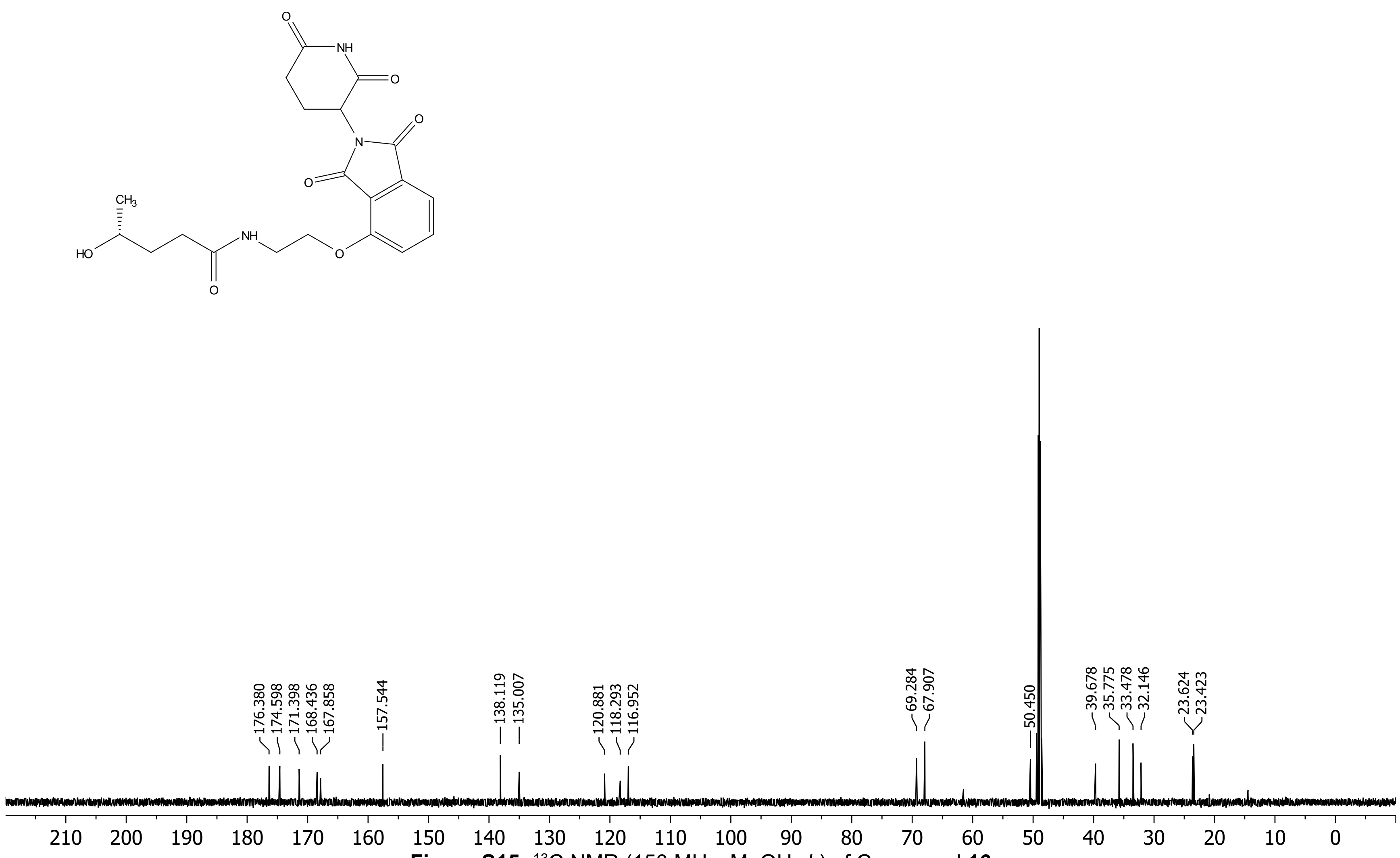

Figure S15: ^{13}C NMR (150 MHz, MeOH-d_4) of Compound **16**

Display Report

Analysis Info

Analysis Name	D:\Data\Kassei\A40\A40_006_20240501-B_P1-A-3_01_842.d	Acquisition Date	5/1/2024 3:55:28 PM
Method	lc_esi_pos_low.m	Operator	8ken
Sample Name	A40_006_20240501-B	Instrument	micrOTOF II 8213750.10430
Comment			

Acquisition Parameter

Source Type	ESI	Ion Polarity	Positive	Set Nebulizer	1.6 Bar
Focus	Not active			Set Dry Heater	200 °C
Scan Begin	50 m/z	Set Capillary	4500 V	Set Dry Gas	7.0 l/min
Scan End	3000 m/z	Set End Plate Offset	-500 V	Set Divert Valve	Waste

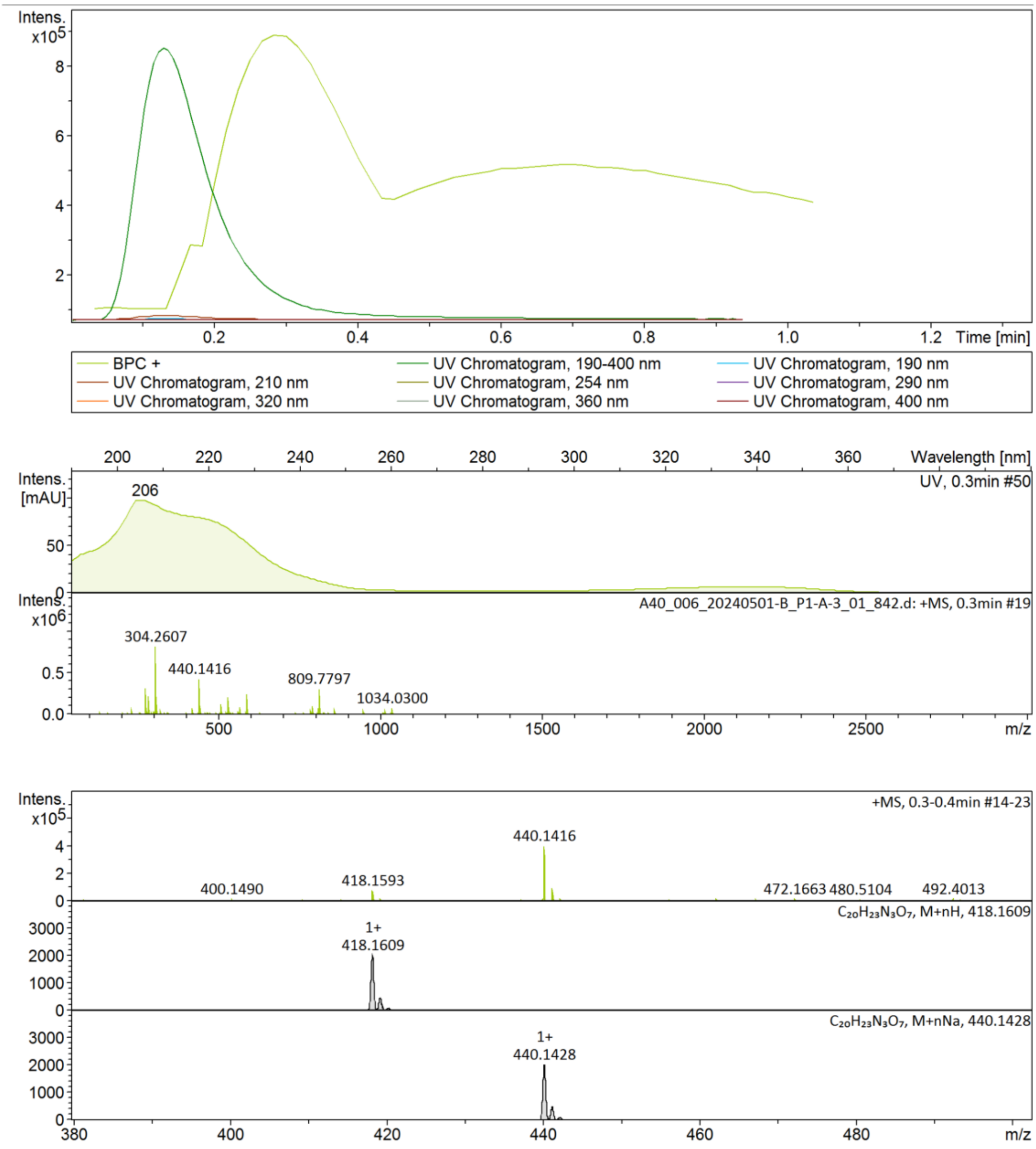

Figure S16: HRMS spectrum of compound **16**

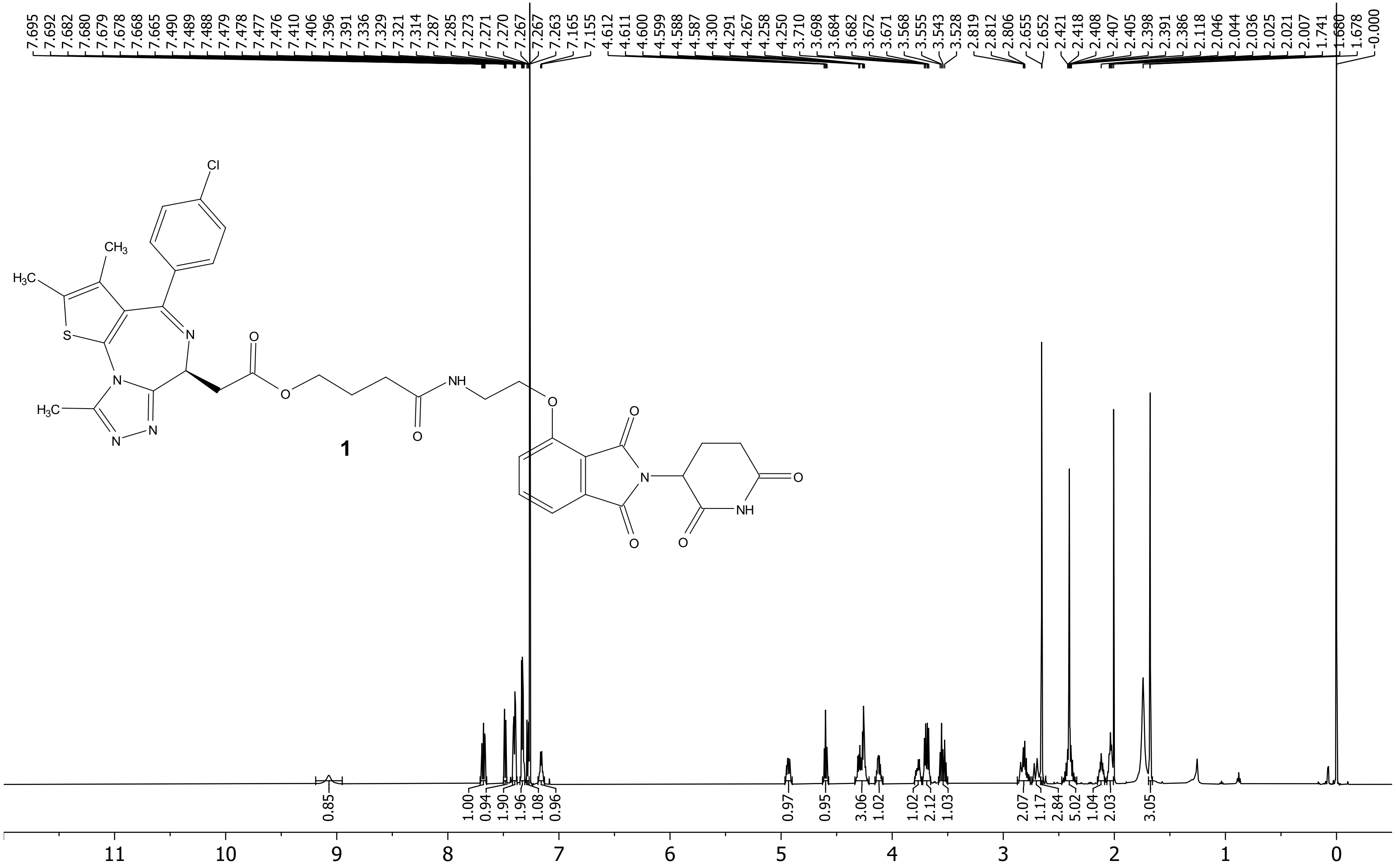

Figure S17: ^{1}H NMR (600 MHz, $CDCl_3$) of Compound **1**

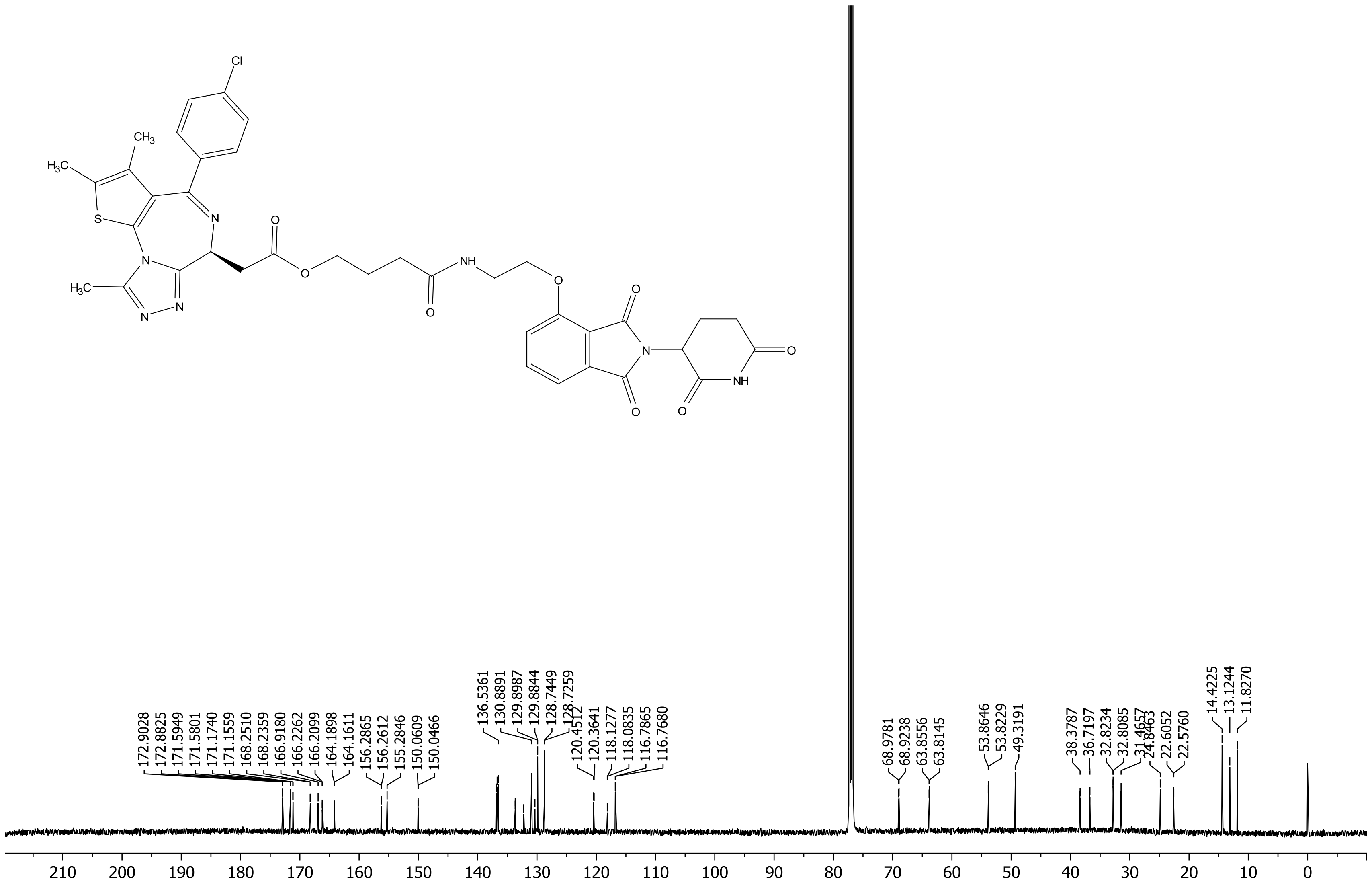

Figure S18: ^{1}H NMR (125 MHz, $CDCl_3$) of Compound **1**

Display Report

Analysis Info

Analysis Name	D:\Data\Kassei\H13\H13_264a.d	Acquisition Date	11/28/2022 6:46:25 PM
Method	LC_esi_pos_low.m	Operator	8ken
Sample Name		Instrument	micrOTOF II 8213750.10430
Comment			

Acquisition Parameter

Source Type	ESI	Ion Polarity	Positive	Set Nebulizer	1.6 Bar
Focus	Not active			Set Dry Heater	200 °C
Scan Begin	50 m/z	Set Capillary	4500 V	Set Dry Gas	7.0 l/min
Scan End	2000 m/z	Set End Plate Offset	-500 V	Set Divert Valve	Waste

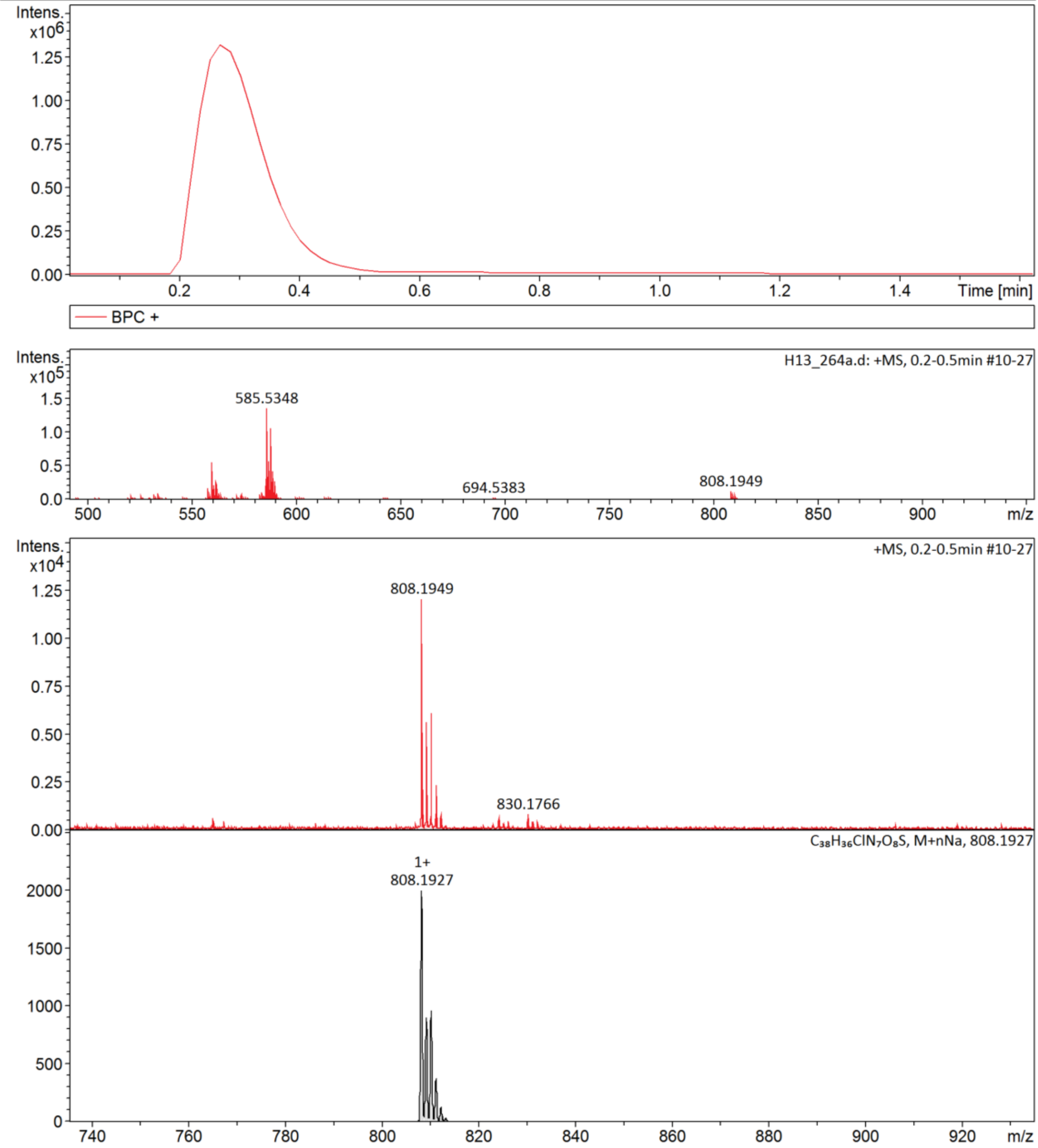

Figure S19: HRMS spectrum of compound **1**

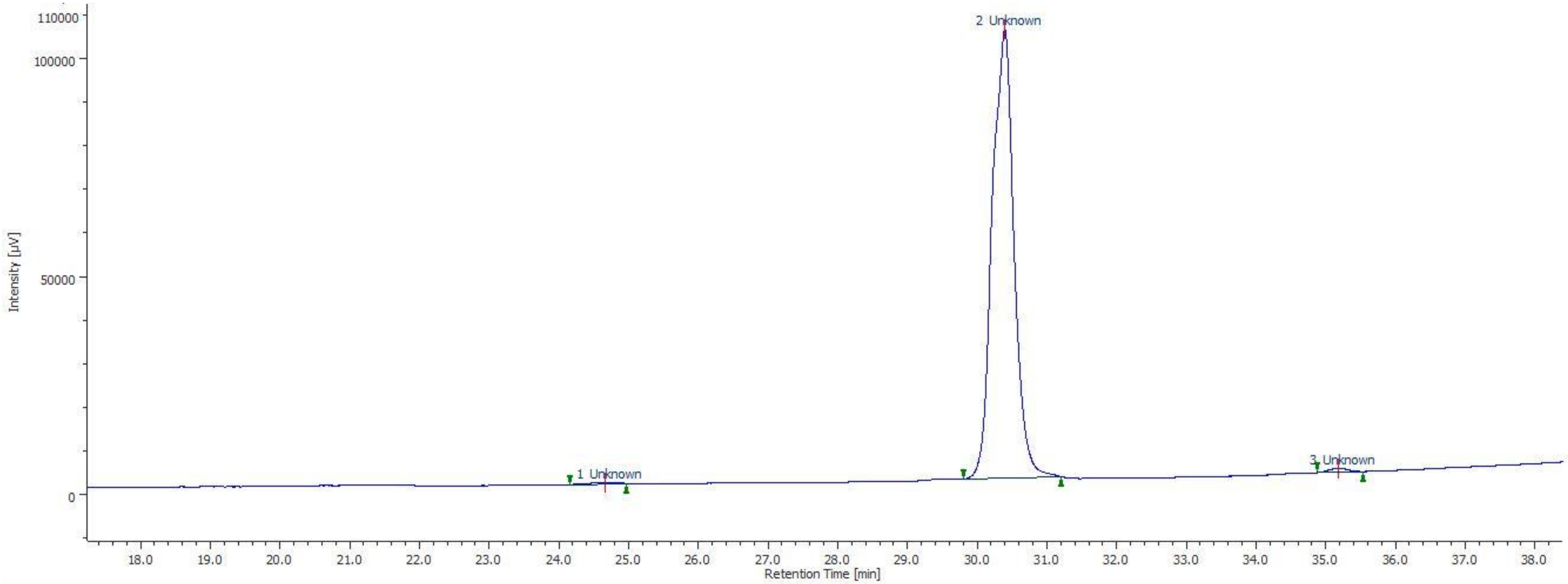

ピーク情報: 2024.7.10 purified 264a 4 − 2024.7.4 早川さんstock 264a 100uM 50uL*

#	ピーク名	tR	面積	高さ	面積%	高さ%	ピークスタート	ピークエンド	ベースラインスタート	ベースラインエンド	検出方法
1	Unknown	24.663	8722	361	0.387	0.348	24.160	24.963	24.160	24.963	マニュアル
2	Unknown	30.387	2227213	102692	98.872	98.786	29.800	31.190	29.800	31.190	マニュアル
3	Unknown	35.173	16697	900	0.741	0.866	34.857	35.523	34.857	35.523	マニュアル

Figure S20: HPLC trace of compound **1**. ‘面積%’ represents Area%.

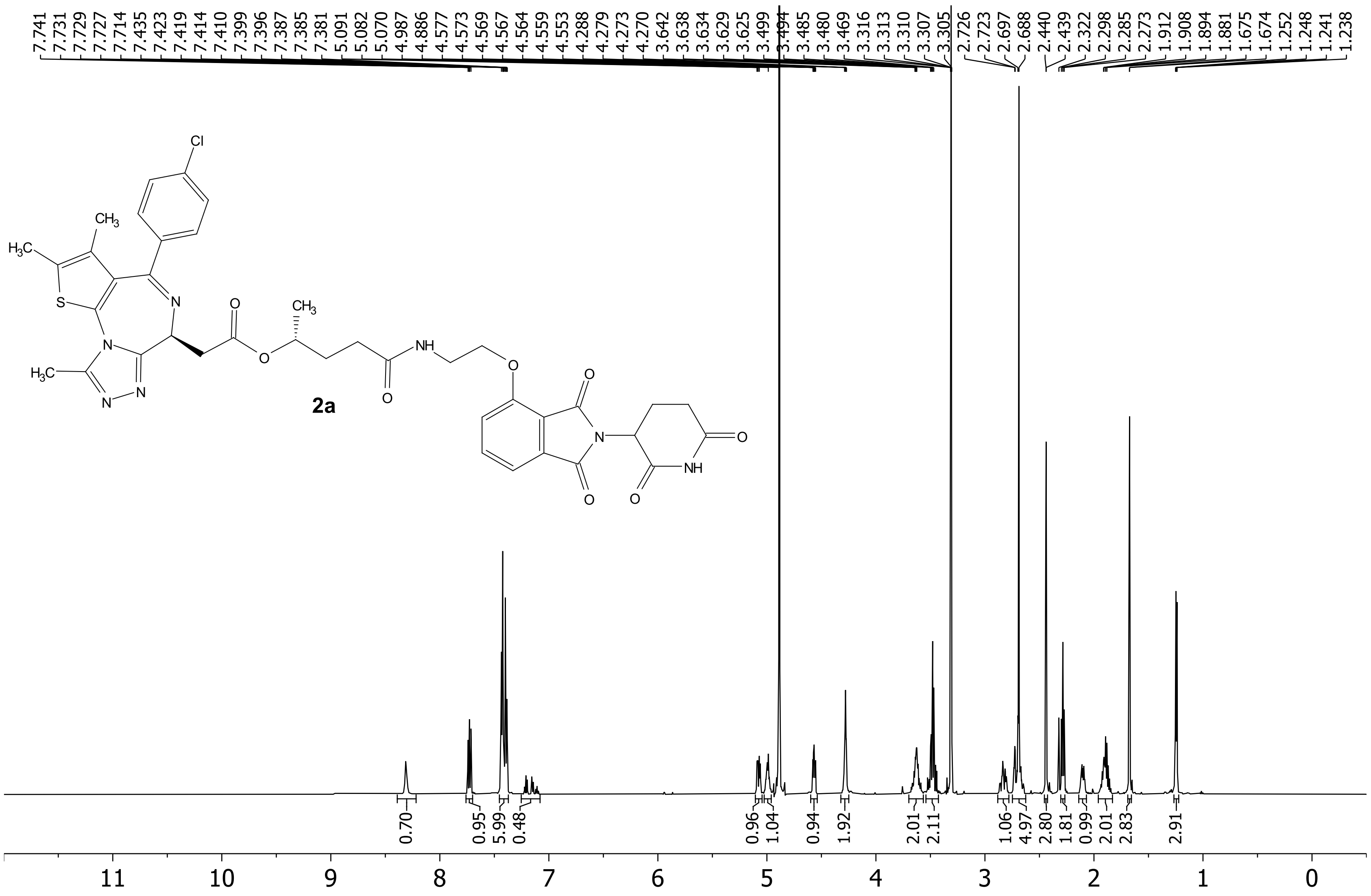

Figure S21: ^{1}H NMR (600 MHz, MeOH-d_4) of Compound **2a**

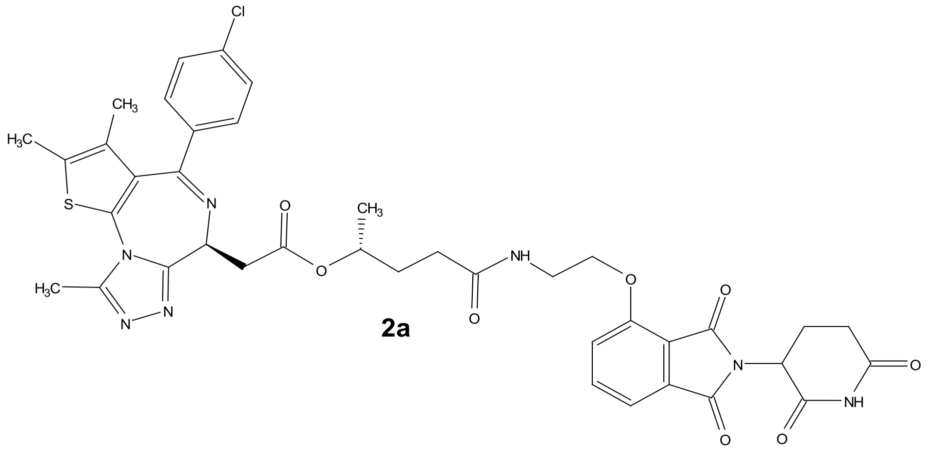

2a

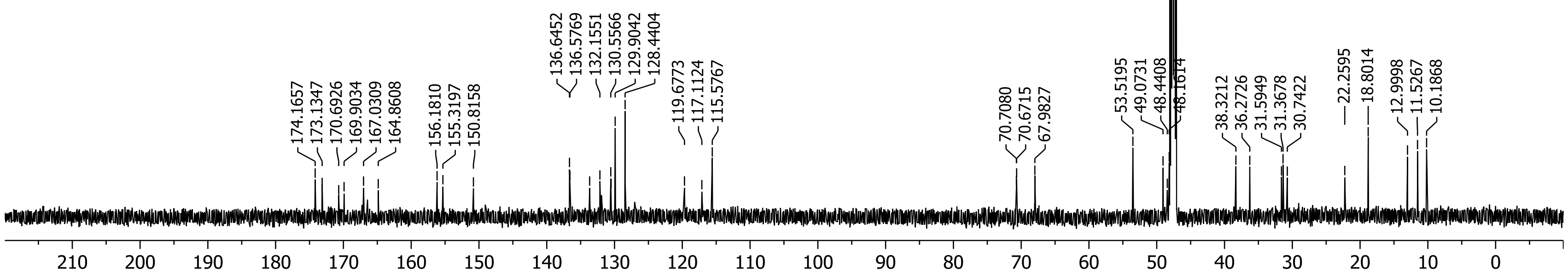

Figure S22: ^{13}C NMR (150 MHz, MeOH-d_4) of Compound **2a**

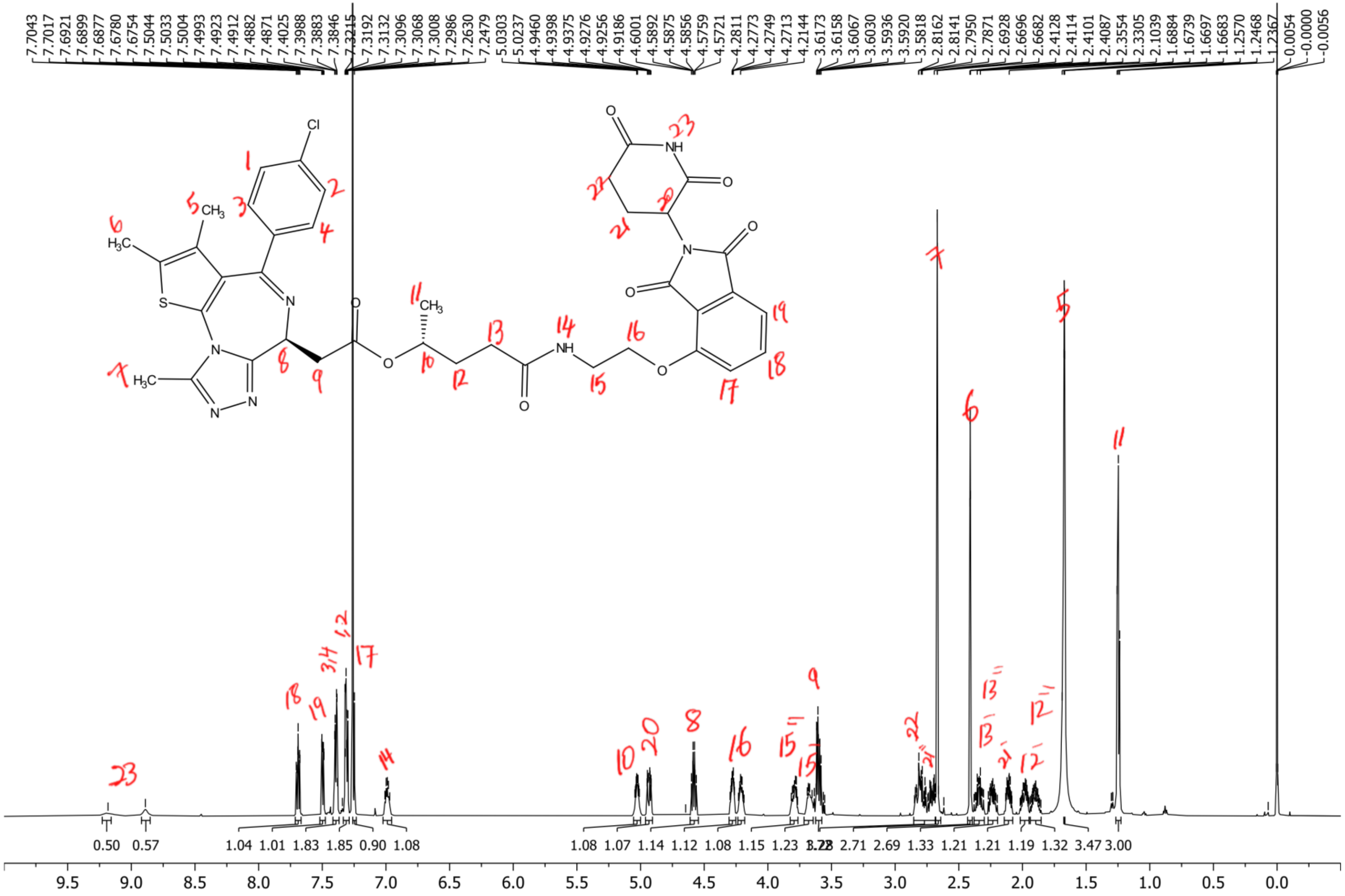

Figure S23: ^1H NMR (600 MHz, $CDCl_3$) of Compound **2a**

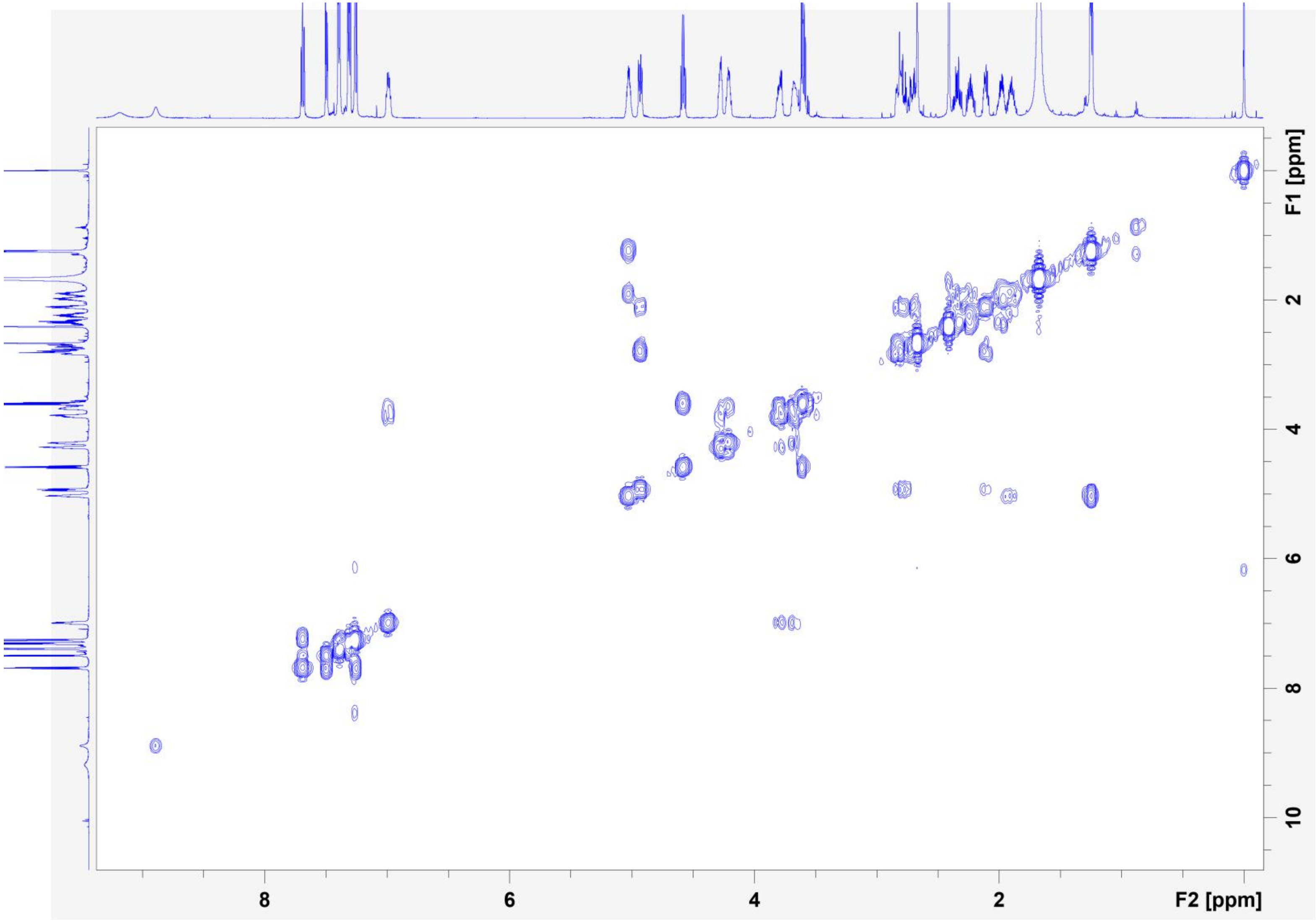

Figure S24: COSY spectrum (600 MHz, $CDCl_3$) of compound **2a**

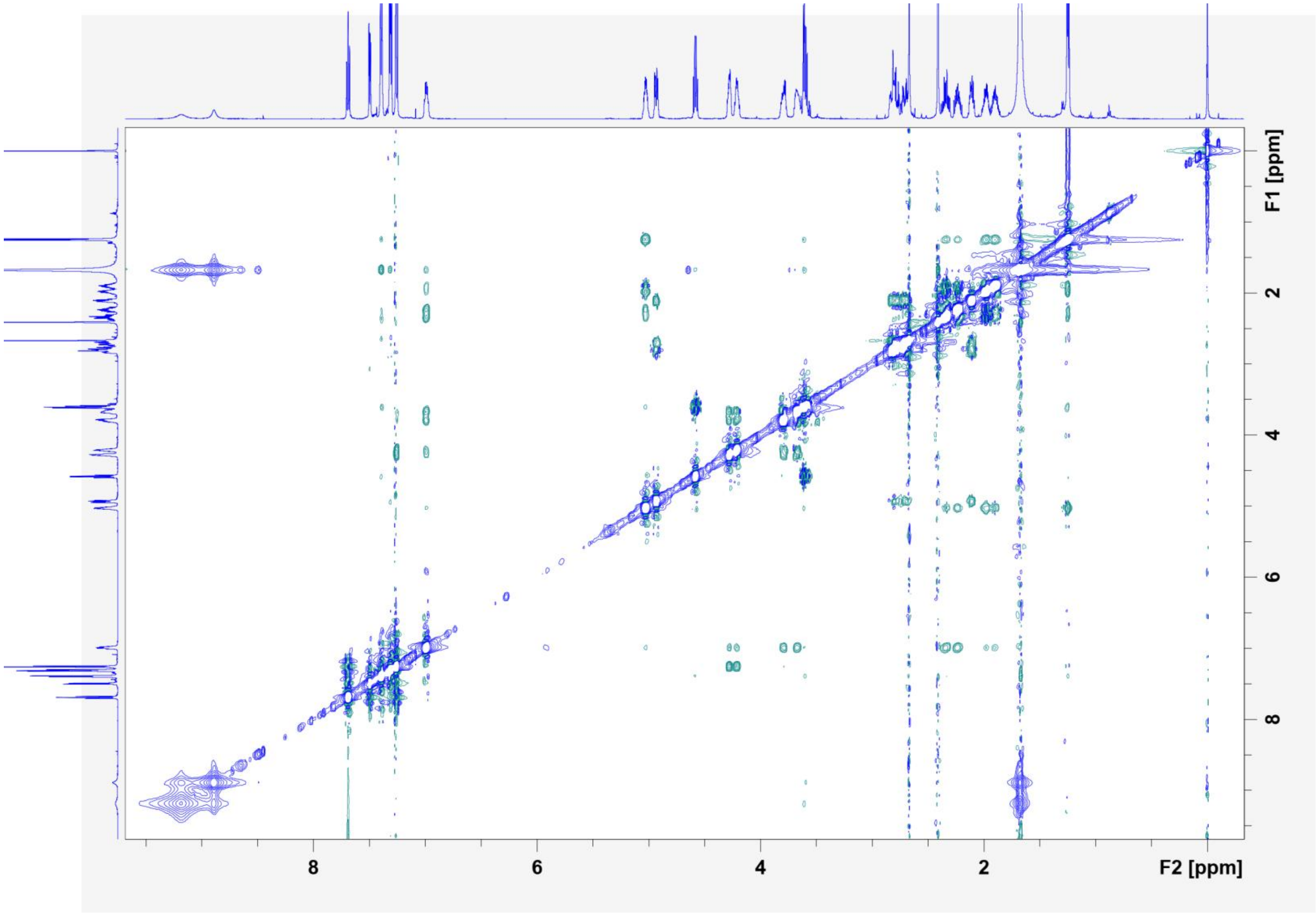

Figure S25: NOESY spectrum (600 MHz, $CDCl_3$) of compound **2a** recorded with a mixing time of 300 ms

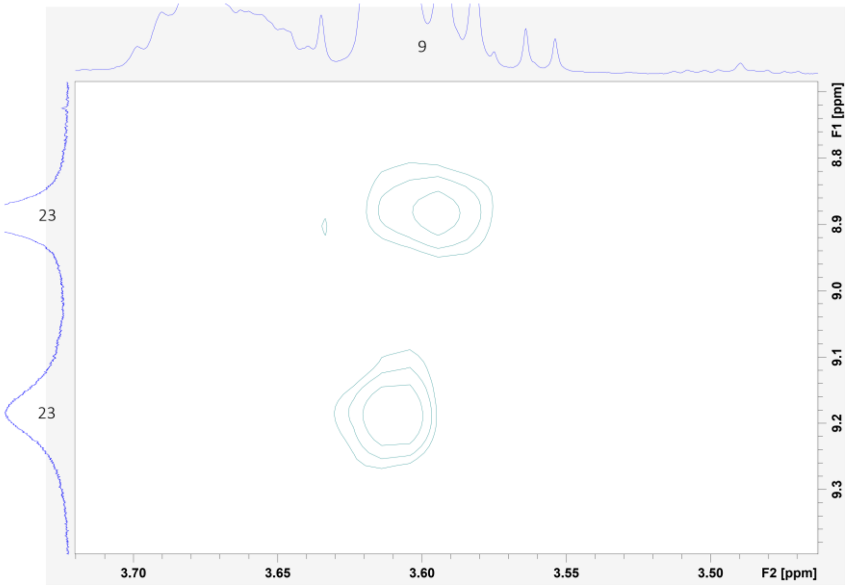

Figure S26: The NOE correlations of **2a** (600 MHz, $CDCl_3$) between -H23 and -H9

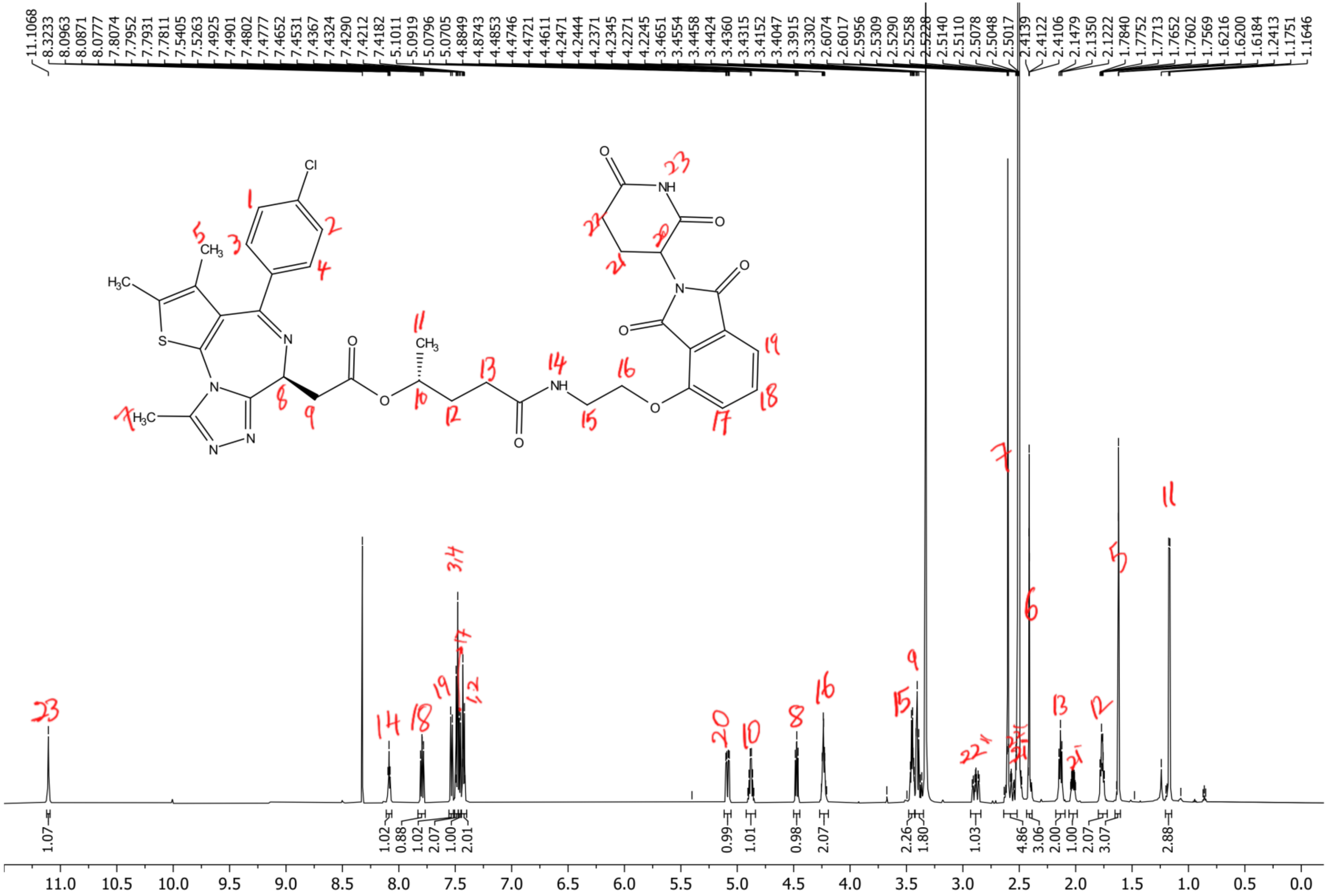

Figure S27: ^{1}H NMR (600 MHz, DMSO-d_6) of Compound **2a**

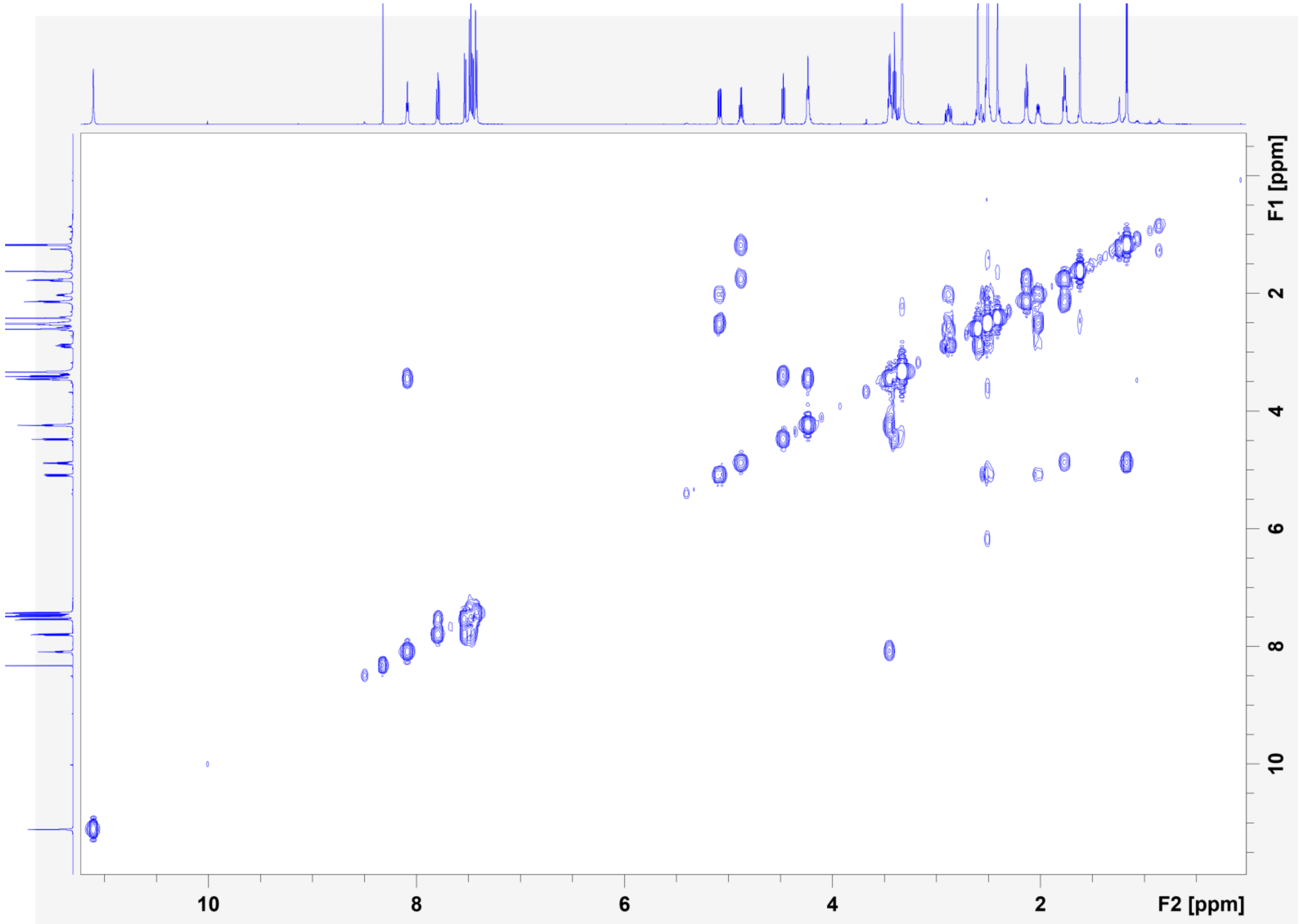

Figure S28: COSY spectrum (600 MHz, DMSO-d_6) of compound **2a**

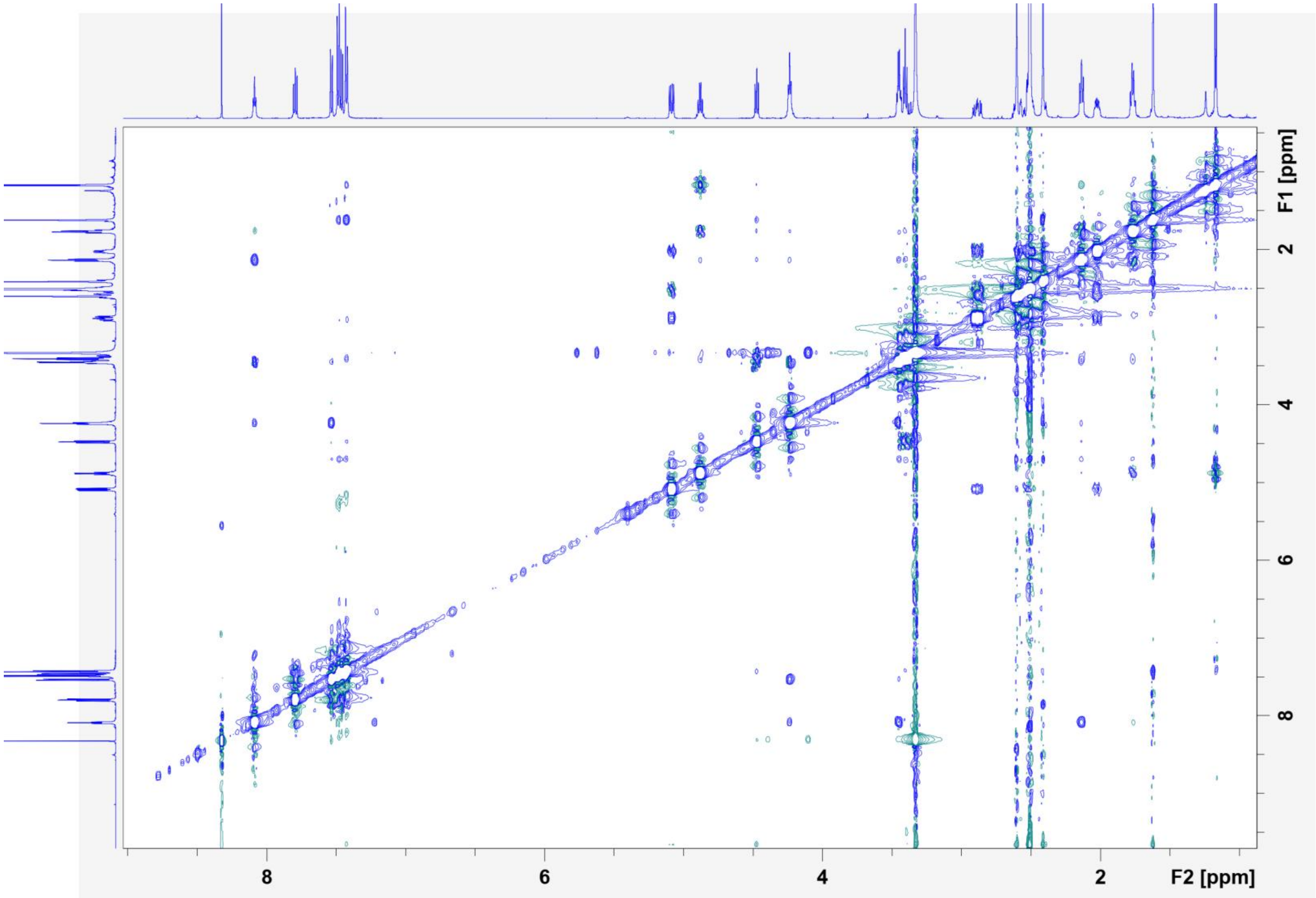

Figure S29: NOESY spectrum (600 MHz, DMSO-d_6) of compound **2a** recorded with a mixing time of 300 ms

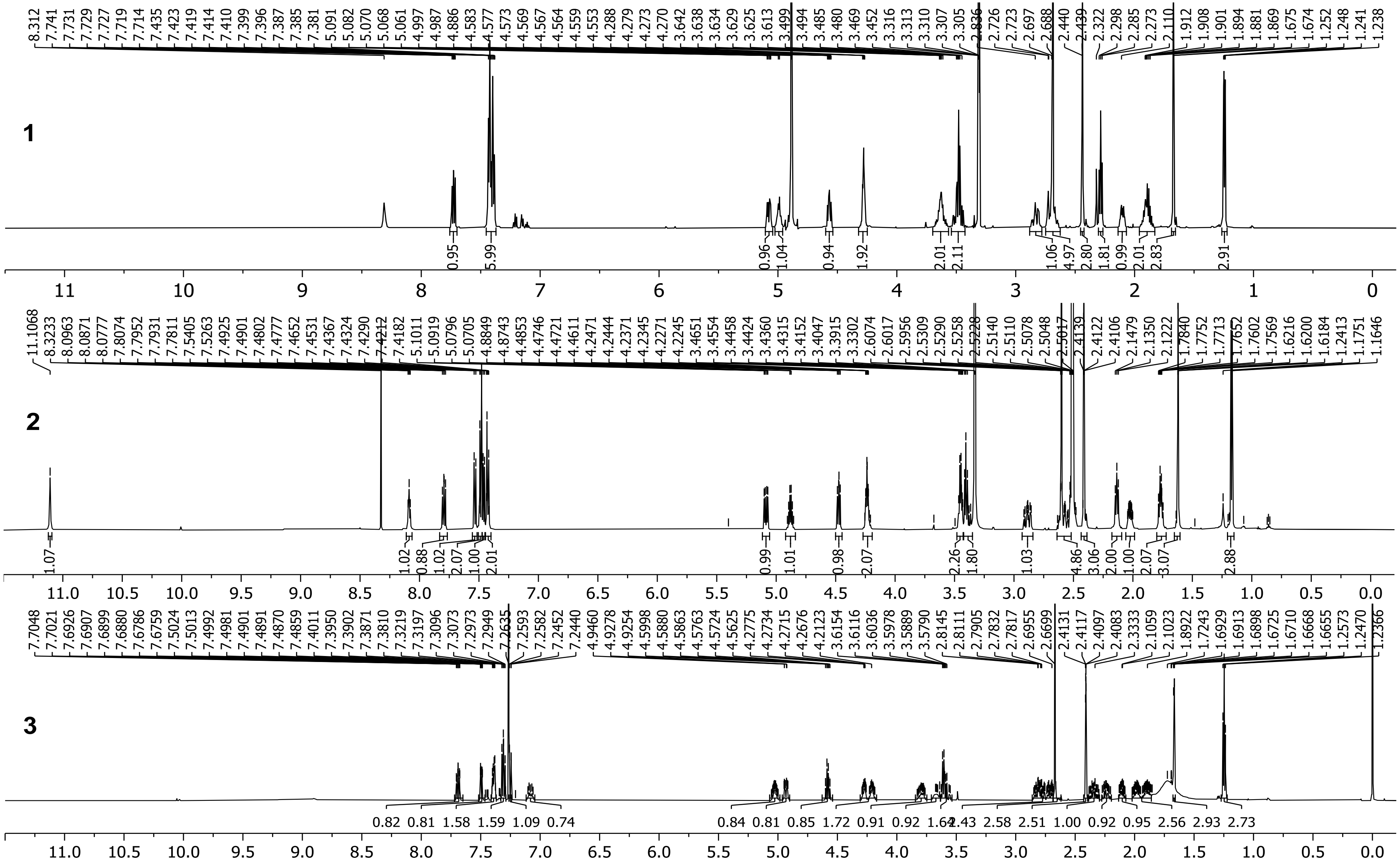

Figure S30: Comparison of the ^{1}H NMR spectra of compound **2a**: 1. (600 MHz, MeOH-d_4), 2. (600 MHz, DMSO-d_6) and 3. (600 MHz, $CDCl_3$)

Display Report

Analysis Info

Analysis Name	D:\Data\Kassei\H03\H03_234a.d	Acquisition Date	12/16/2021 6:43:51 PM
Method	LC_esi_pos_low.m	Operator	8ken
Sample Name	ESI-L	Instrument	micrOTOF II 8213750.10430
Comment			

Acquisition Parameter

Source Type	ESI	Ion Polarity	Positive	Set Nebulizer	1.6 Bar
Focus	Not active			Set Dry Heater	200 °C
Scan Begin	50 m/z	Set Capillary	4500 V	Set Dry Gas	7.0 l/min
Scan End	3000 m/z	Set End Plate Offset	-500 V	Set Divert Valve	Waste

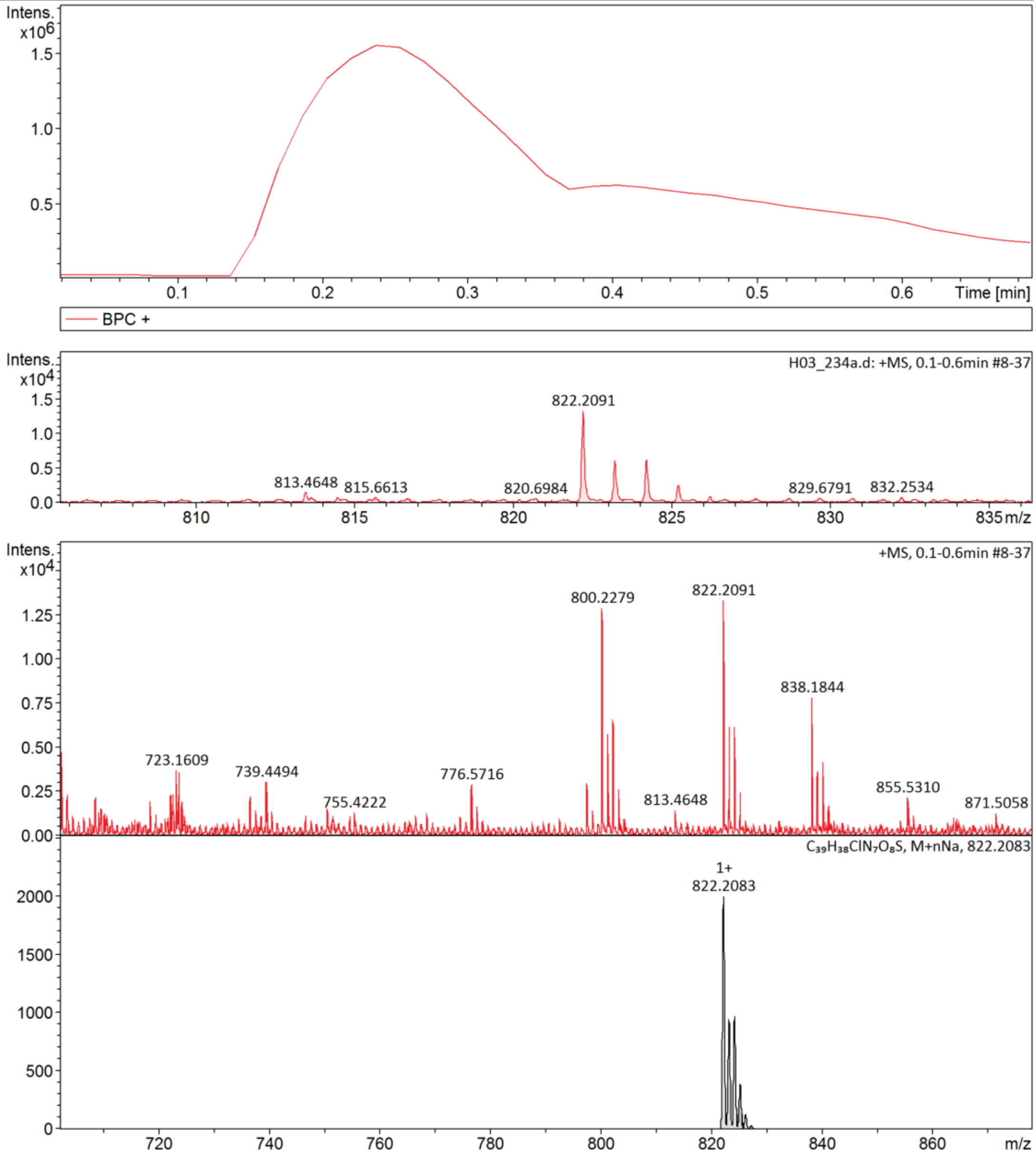

Figure S31: HRMS spectrum of compound **2a**

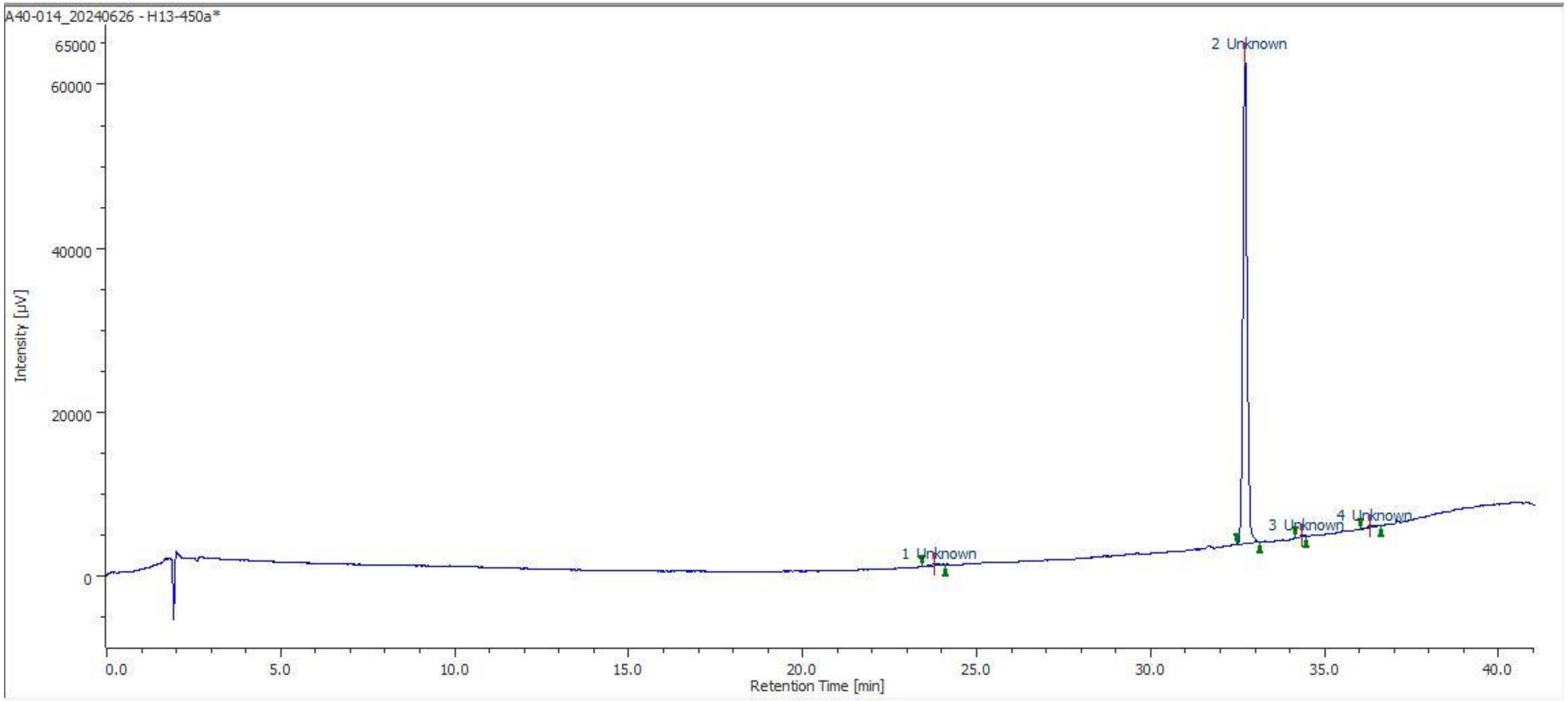

ピーク情報: A40-014_20240626 - H13-450a*

#	ピーク名	tR	面積	高さ	面積%	高さ%	ピークスタート	ピークエンド	ベースラインスタート	ベースラインエンド	検出方法
1	Unknown	23.767	3518	254	0.680	0.419	23.413	24.077	23.413	24.077	マニュアル
2	Unknown	32.683	509712	59714	98.527	98.705	32.440	33.103	32.440	33.103	マニュアル
3	Unknown	34.327	1327	223	0.256	0.369	34.120	34.433	34.120	34.433	マニュアル
4	Unknown	36.277	2774	307	0.536	0.507	35.997	36.583	35.997	36.583	マニュアル

Figure S32: HPLC trace of compound **2a**. '面積%' represents Area%.

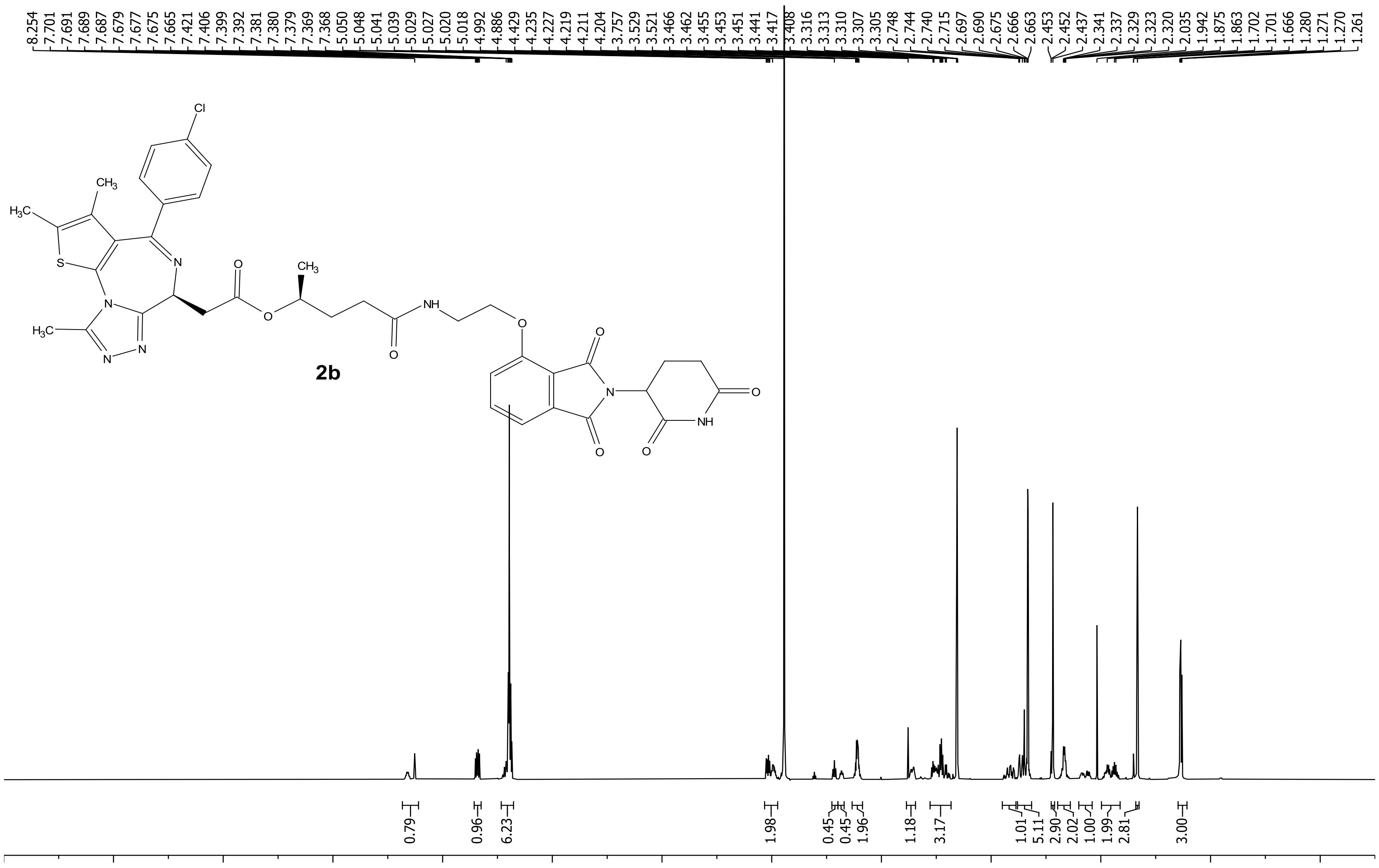

Figure S33: ^{1}H NMR (600 MHz, MeOH-d_4) of Compound **2b**

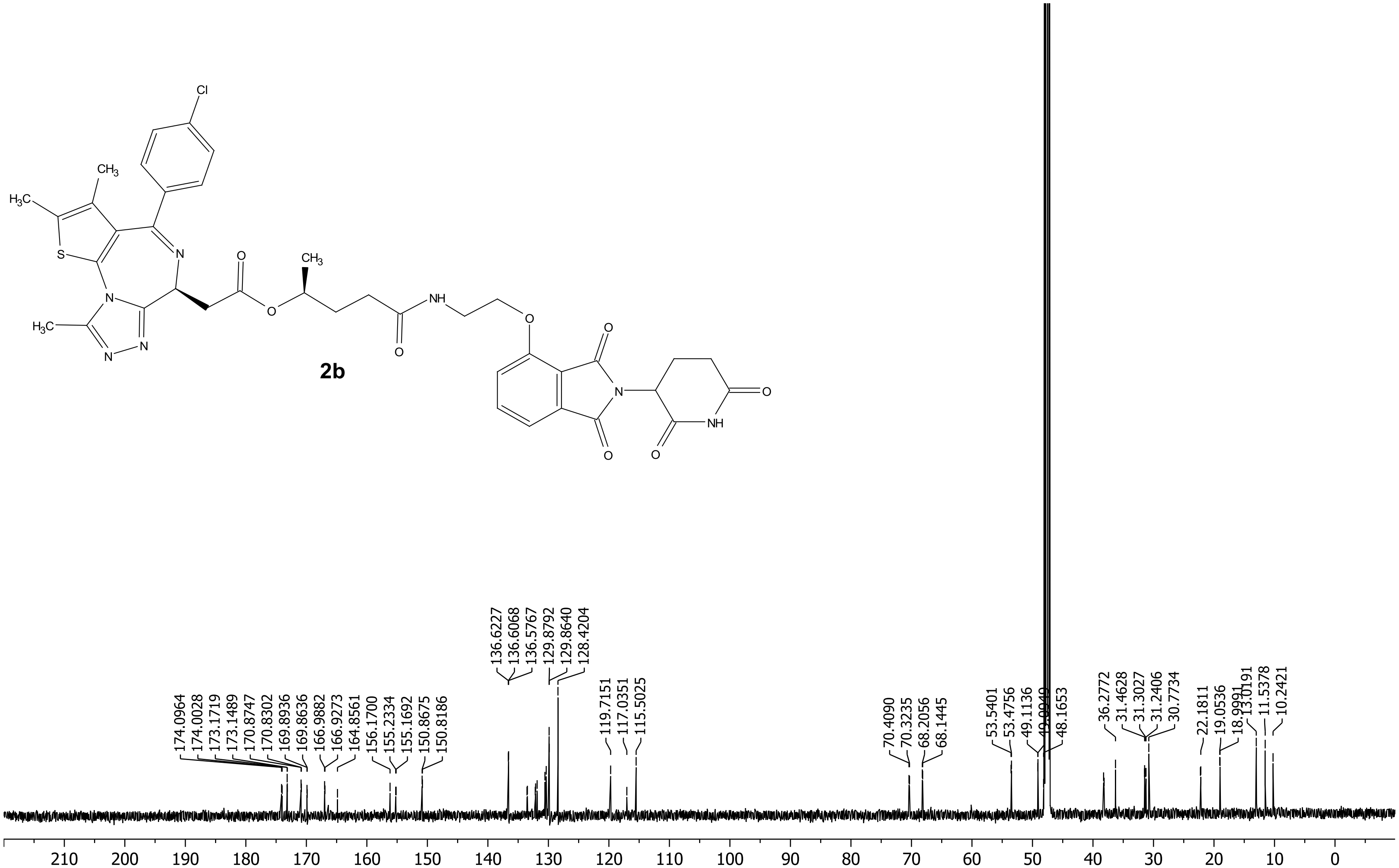

Figure S34: ^{13}C NMR (150 MHz, MeOH-d_4) of Compound **2b**

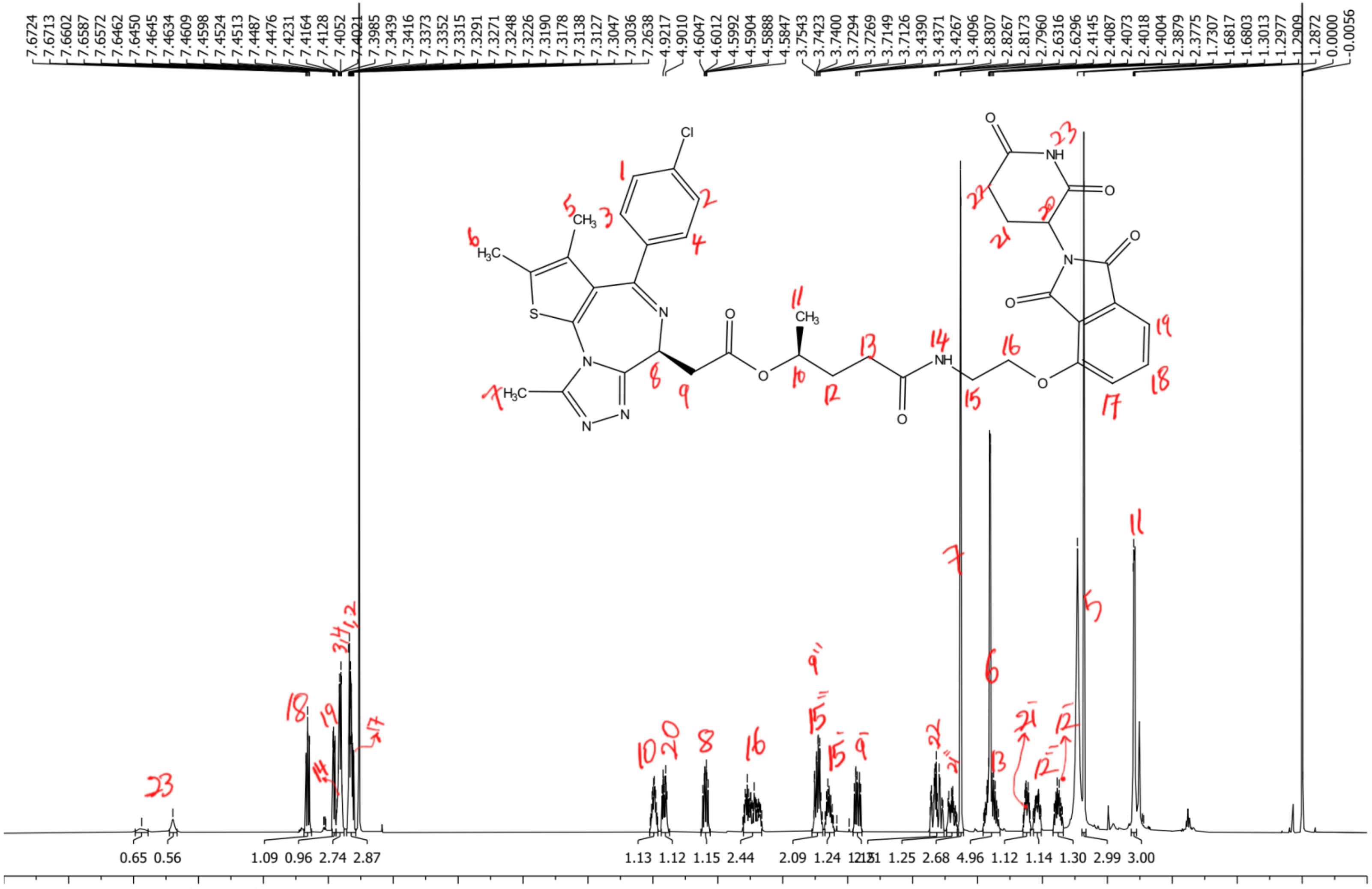

Figure S35: ^{1}H NMR (600 MHz, $CDCl_3$) of Compound **2b**

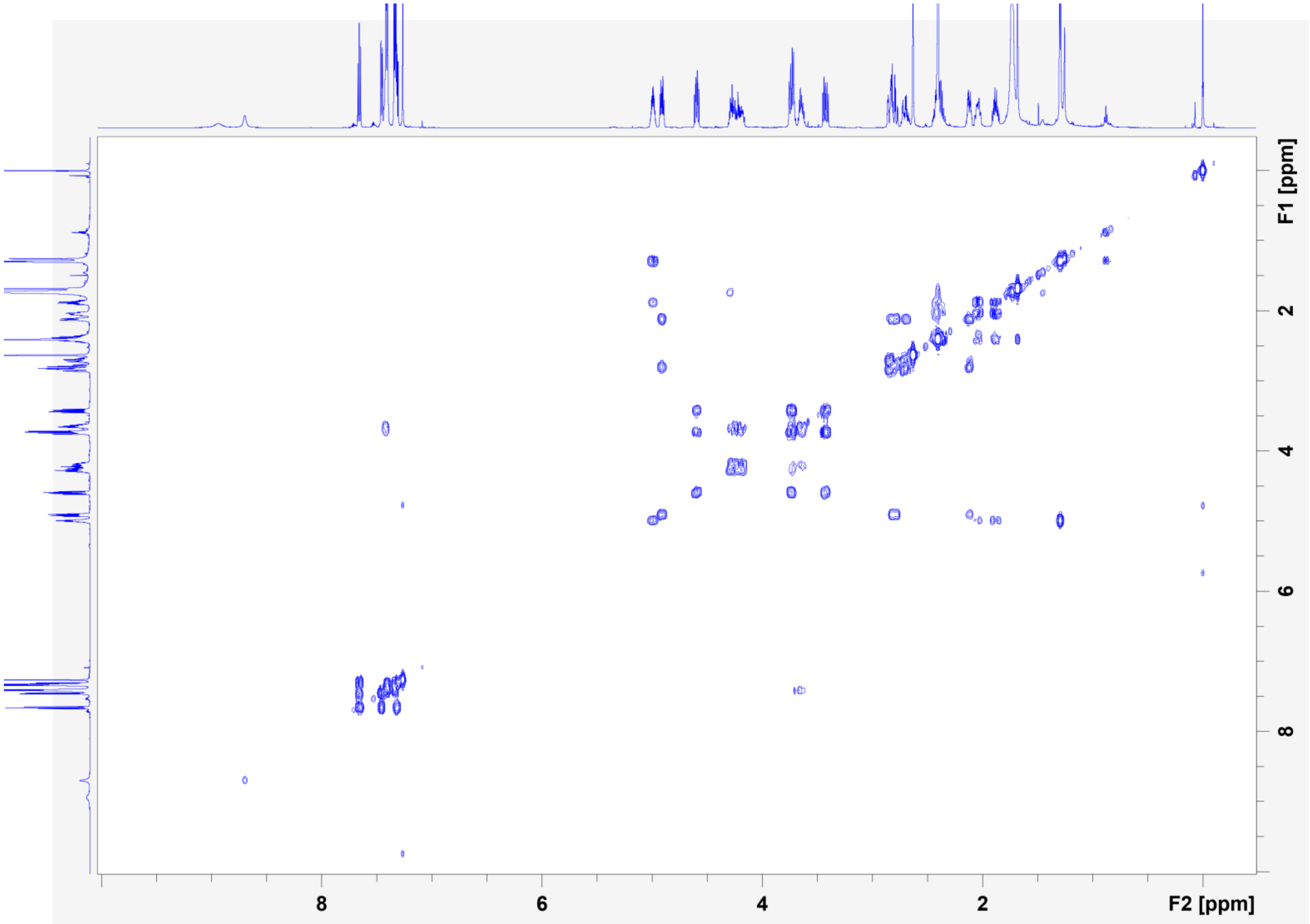

Figure S36: COSY spectrum (600 MHz, $CDCl_3$) of compound **2b**

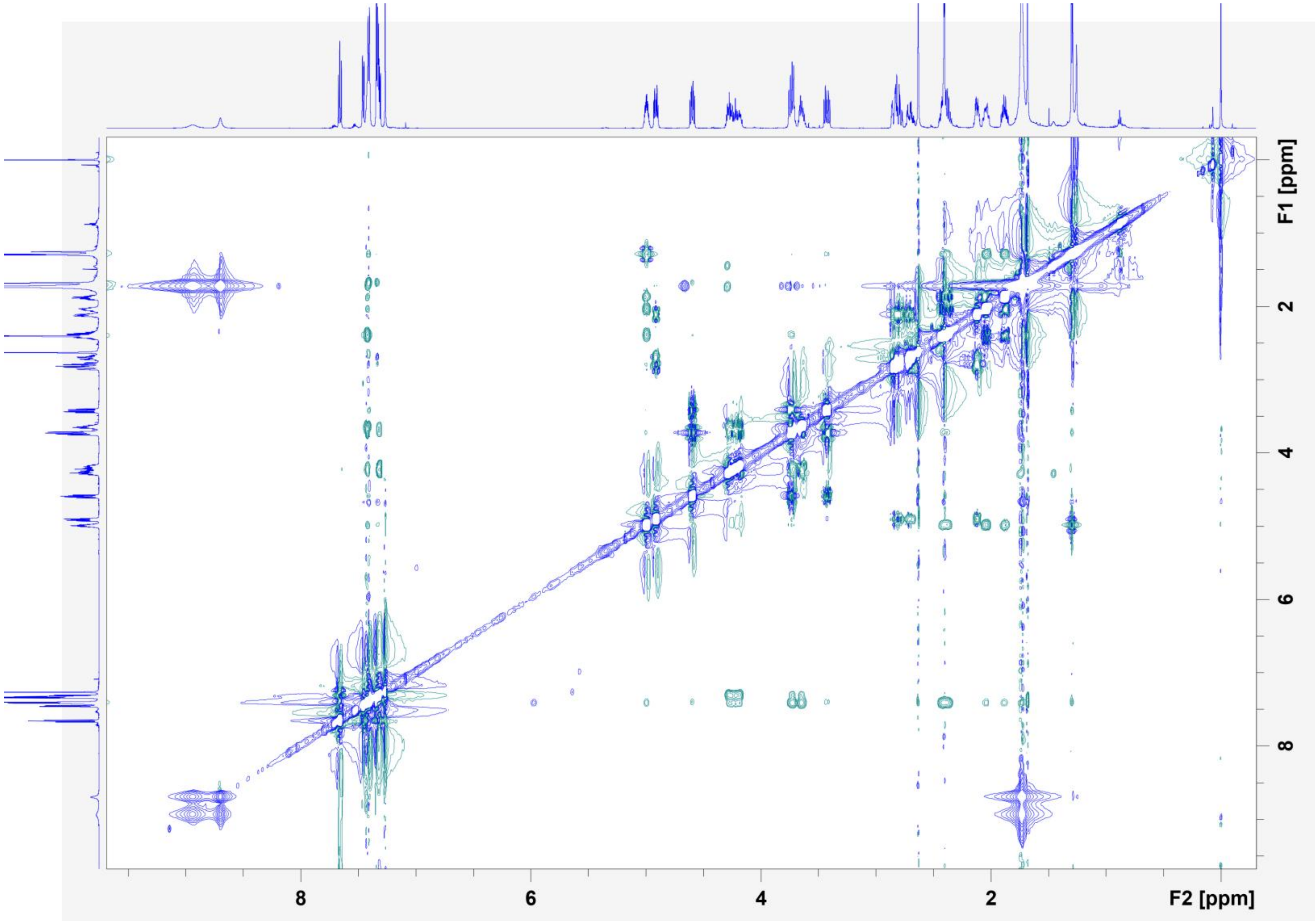

Figure S37: NOESY spectrum (600 MHz, $CDCl_3$) of compound **2b** recorded with a mixing time of 300 ms

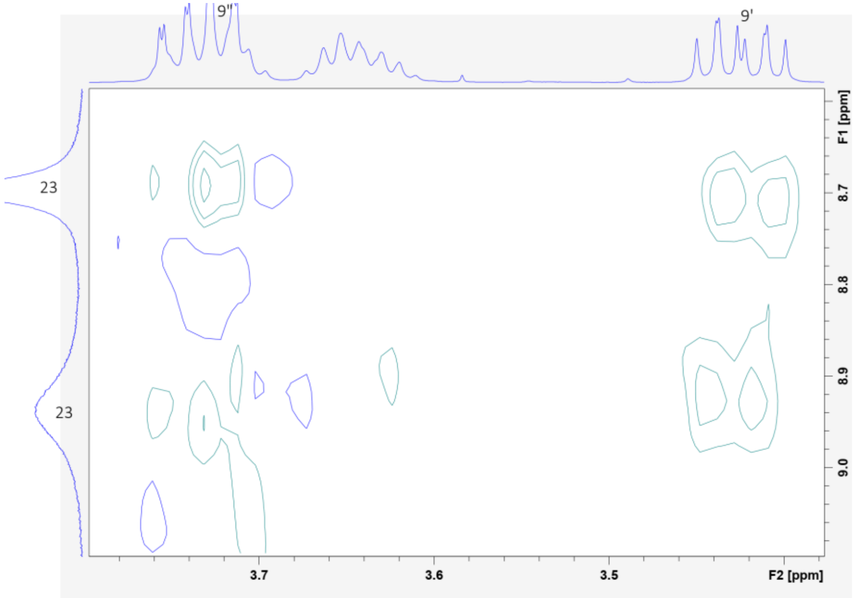

Figure S38: The NOE correlations of **2b** (600 MHz, $CDCl_3$) between -H23 and -H9

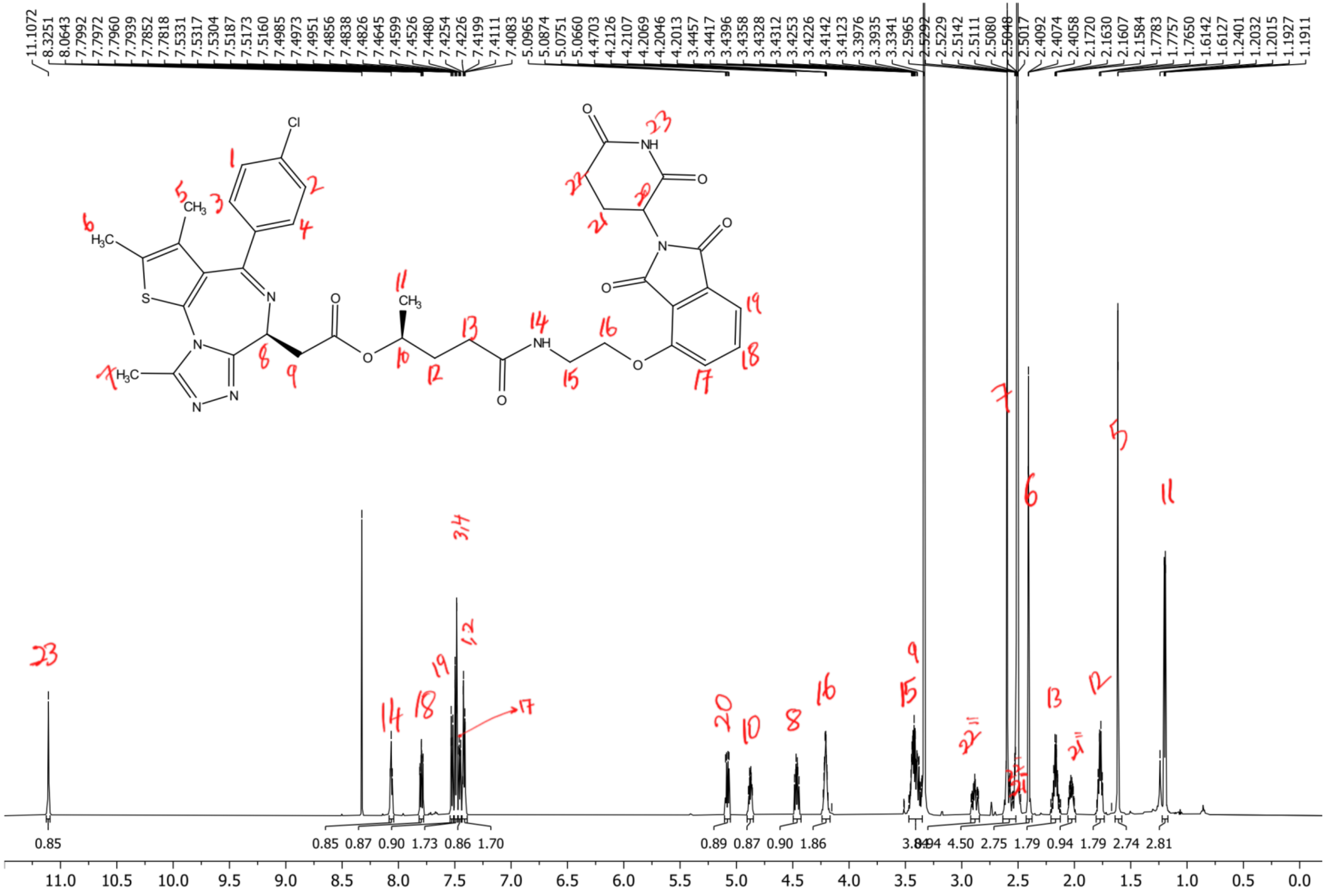

Figure S39: ^{1}H NMR (600 MHz, DMSO-d_6) of Compound **2b**

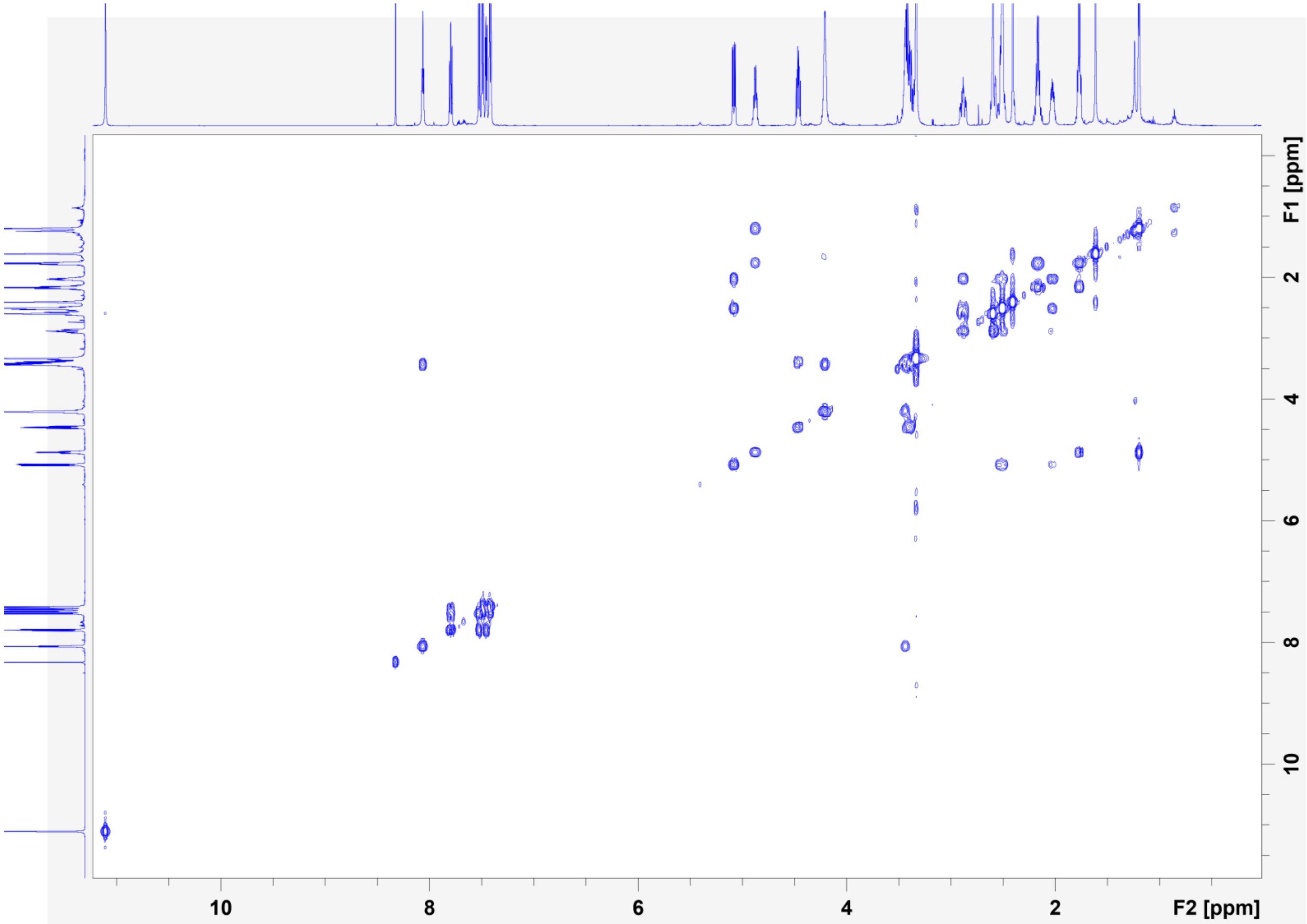

Figure S40: COSY spectrum (600 MHz, DMSO-d_6) of compound **2b**

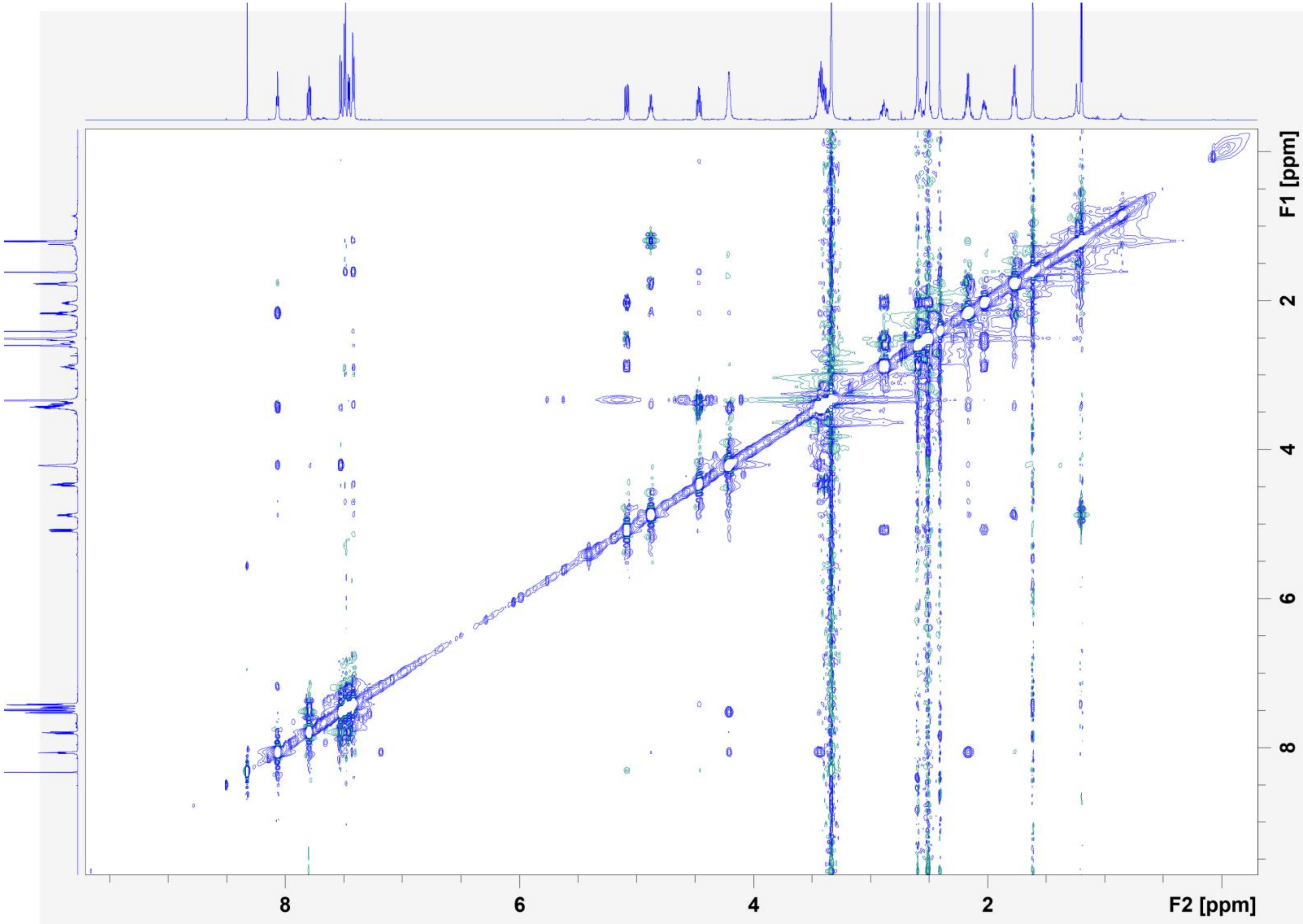

Figure S41: NOESY spectrum (600 MHz, DMSO-d_6) of compound **2b** recorded with a mixing time of 300 ms

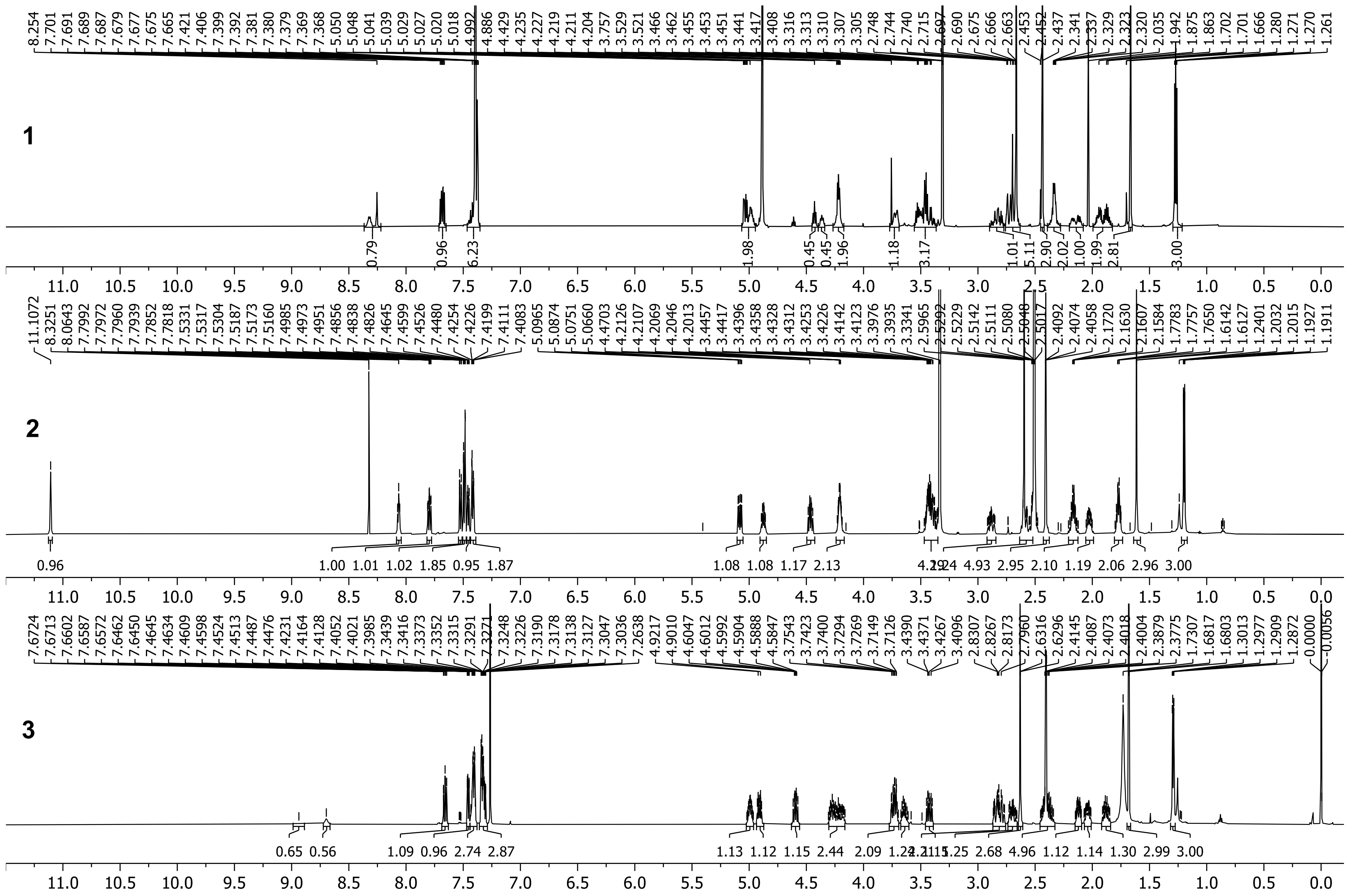

Figure S42: Comparison of the ^{1}H NMR spectra of compound **2b**: 1. (600 MHz, MeOH-d_4), 2. (600 MHz, DMSO-d_6) and 3. (600 MHz, $CDCl_3$)

Display Report

Analysis Info

Analysis Name	D:\Data\Kassei\H03\H03_234b2.d	Acquisition Date	12/17/2021 3:09:59 AM
Method	LC_esi_pos_low.m	Operator	8ken
Sample Name	ESI-L	Instrument	micrOTOF II 8213750.10430
Comment			

Acquisition Parameter

Source Type	ESI	Ion Polarity	Positive	Set Nebulizer	1.6 Bar
Focus	Not active			Set Dry Heater	200 °C
Scan Begin	50 m/z	Set Capillary	4500 V	Set Dry Gas	7.0 l/min
Scan End	3000 m/z	Set End Plate Offset	-500 V	Set Divert Valve	Waste

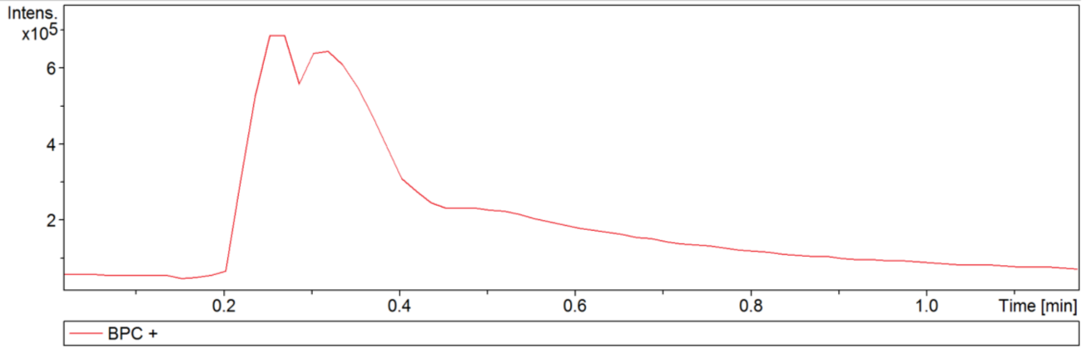

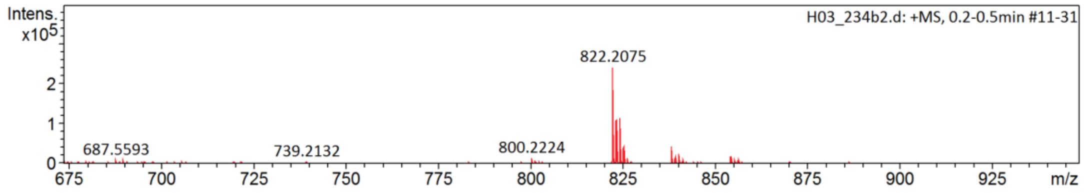

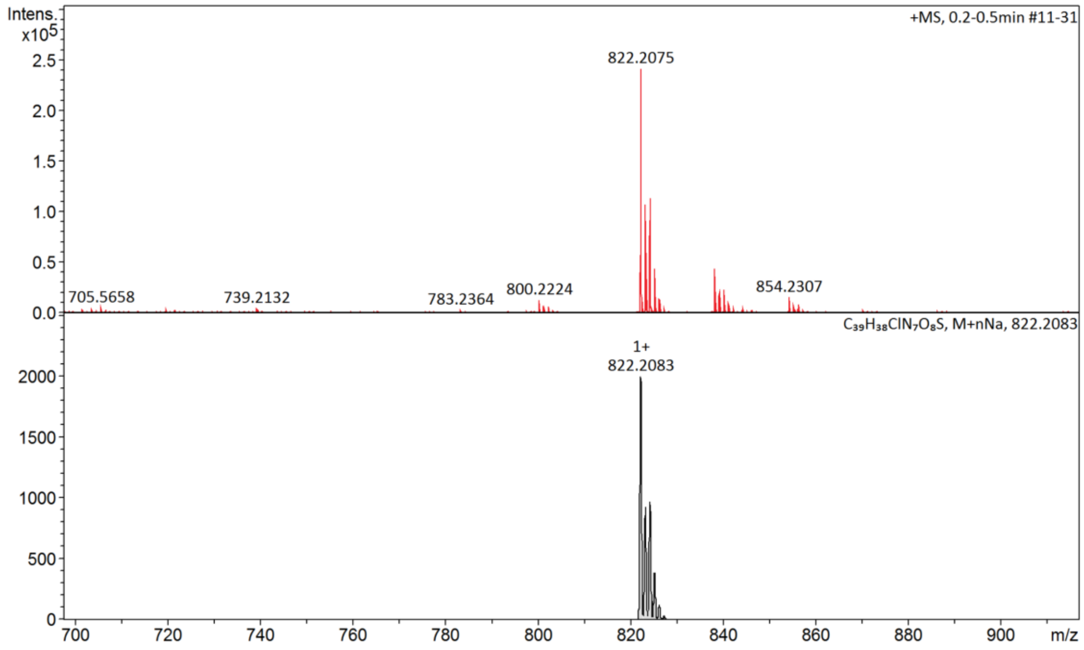

Figure S43: HRMS spectrum of compound **2b**

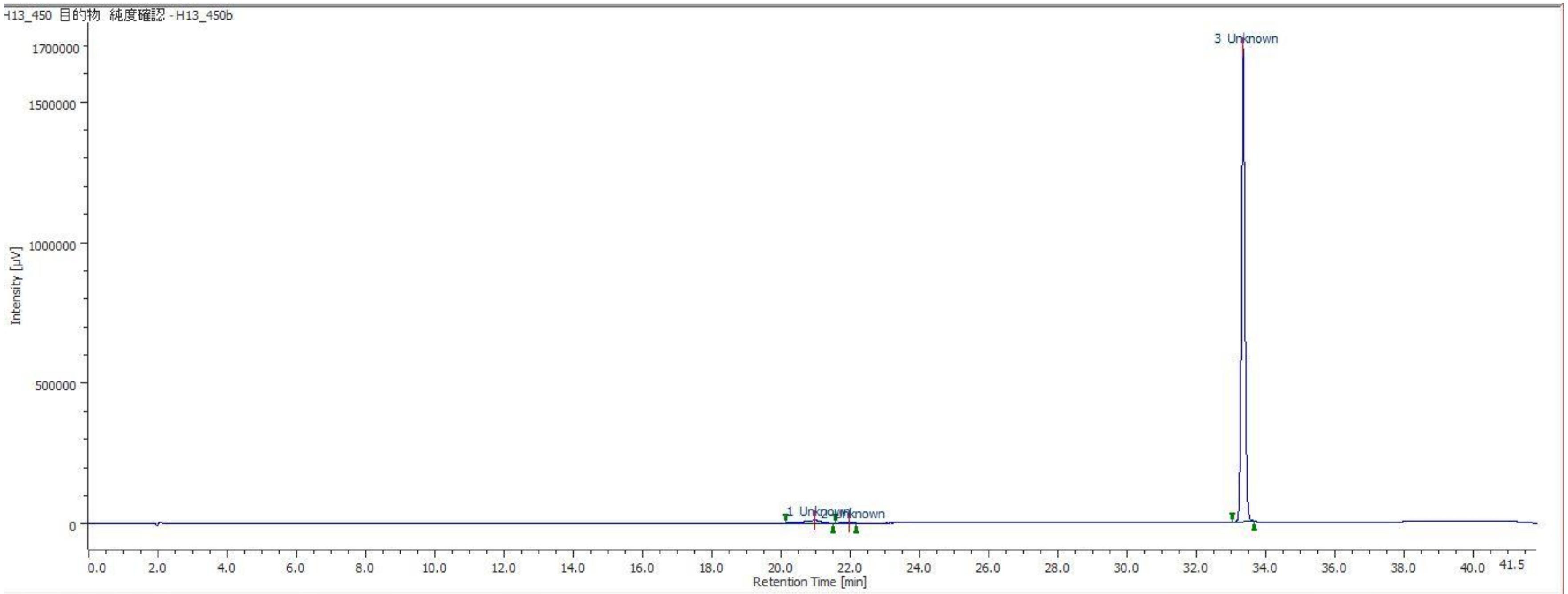

ピーク情報: H13_450 目的物 純度確認 – H13_450b

#	ピーク名	tR	面積	高さ	面積%	高さ%	ピークスタート	ピークエンド	ベースラインスタート	ベースラインエンド	検出方法
1	Unknown	20.950	247244	8923	2.022	0.526	20.123	21.477	20.123	21.477	マニュアル
2	Unknown	21.957	15452	1131	0.126	0.067	21.557	22.157	21.557	22.157	マニュアル
3	Unknown	33.323	11964944	1685023	97.852	99.407	32.993	33.630	32.993	33.630	マニュアル

Figure S44: HPLC trace of compound **2b**. '面積%' represents Area%.

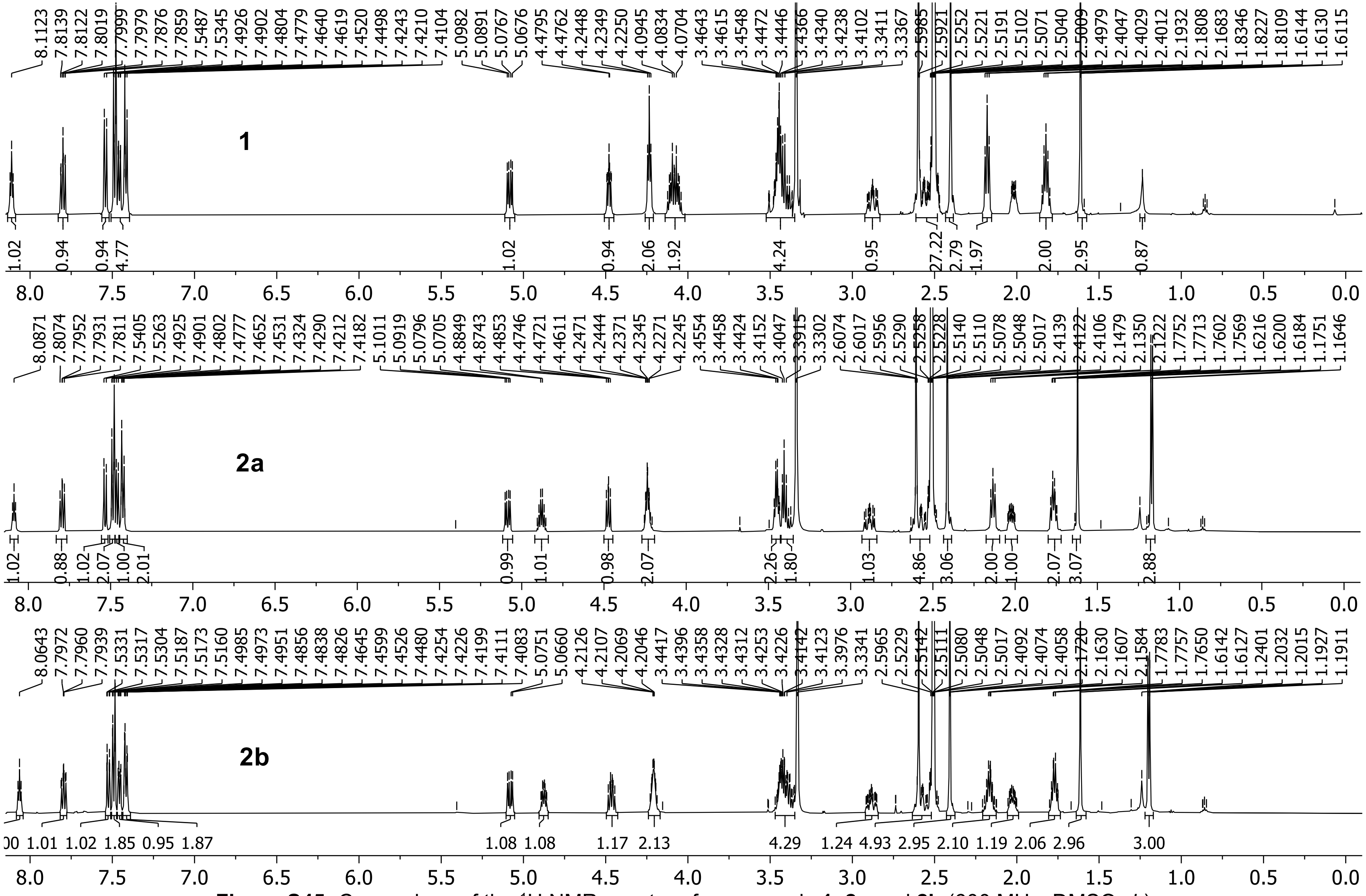

Figure S45: Comparison of the ^{1}H NMR spectra of compounds **1**, **2a** and **2b** (600 MHz, DMSO-d_6)

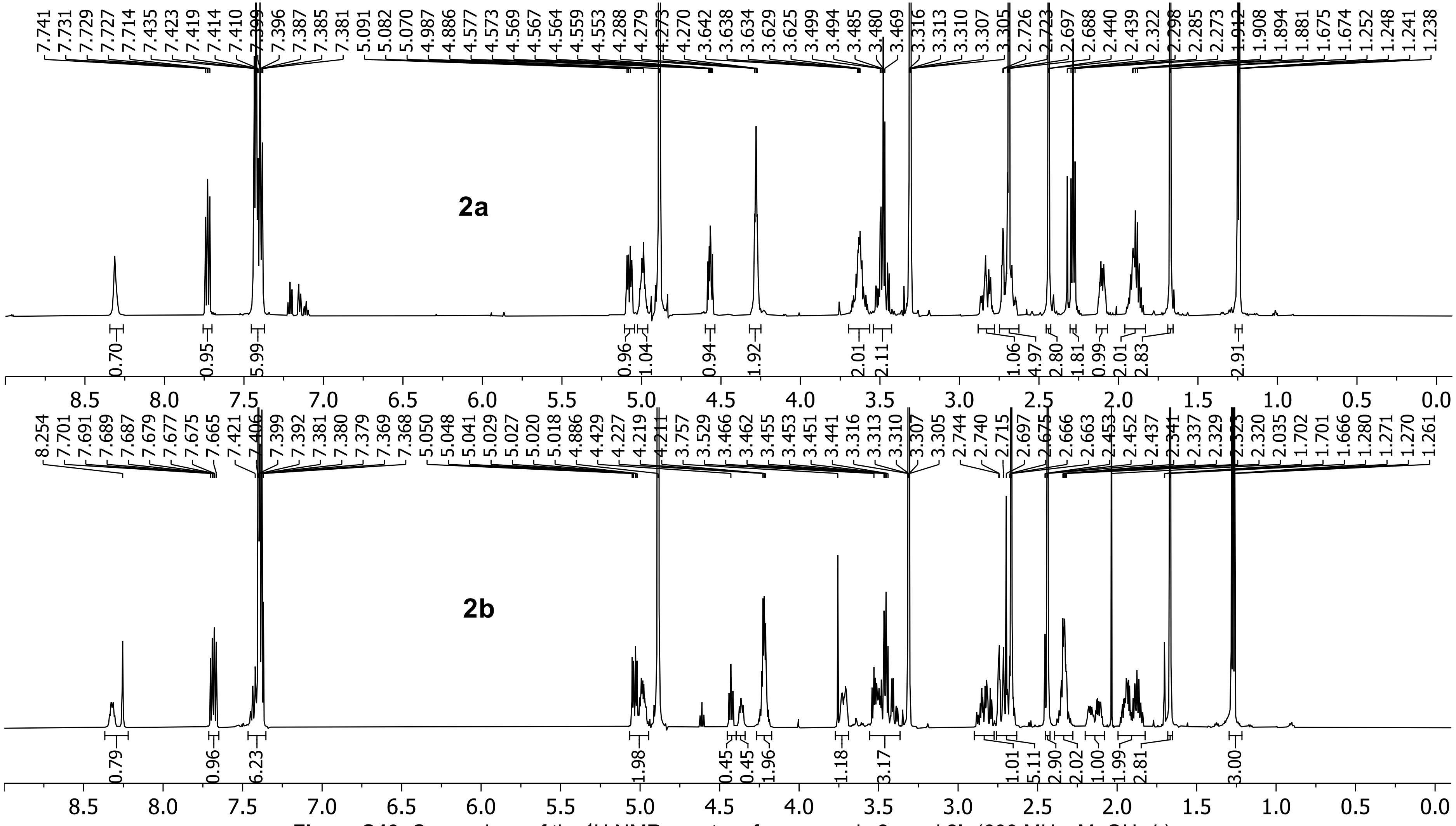

Figure S46: Comparison of the ^{1}H NMR spectra of compounds **2a** and **2b** (600 MHz, MeOH-d_4)

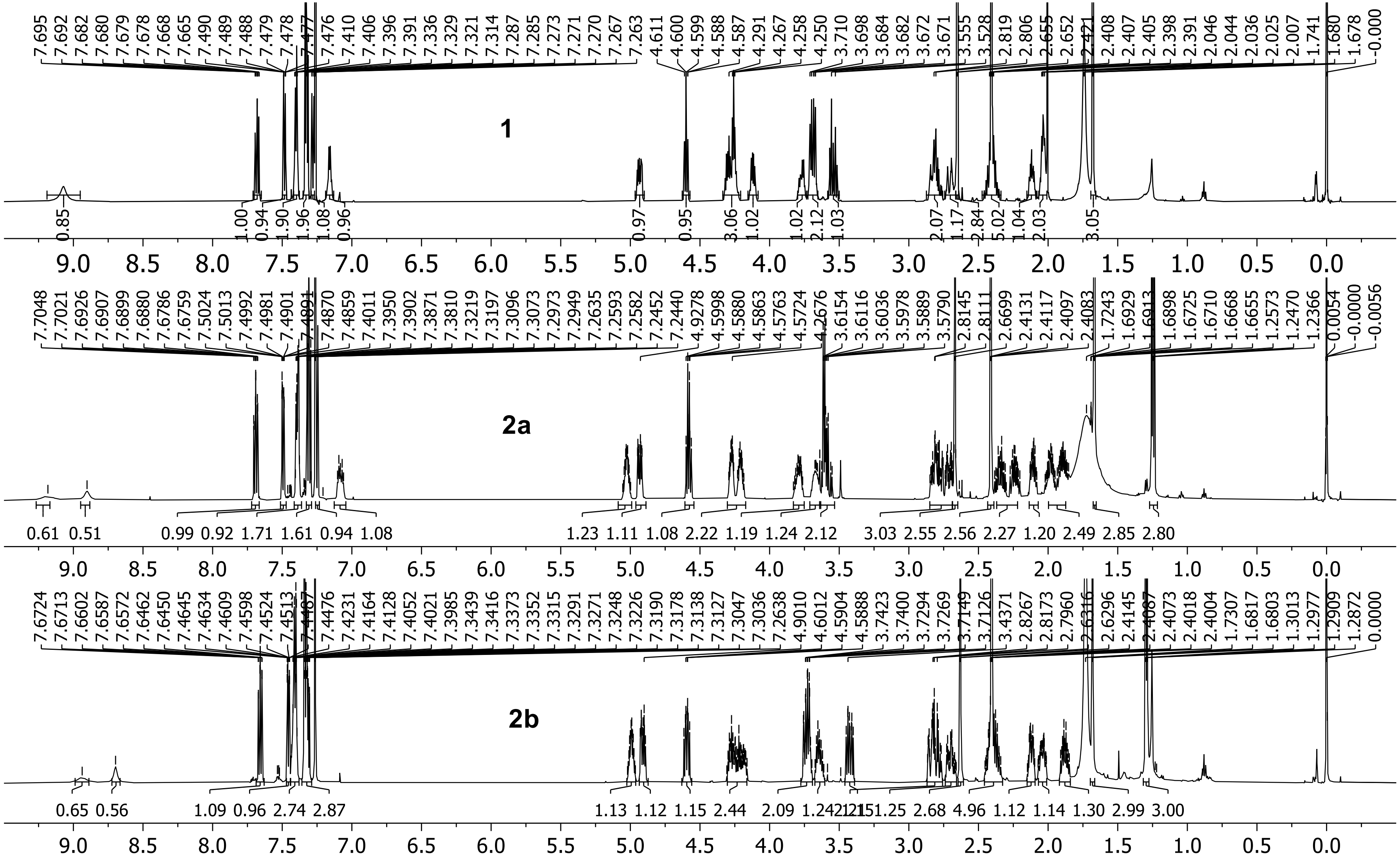

Figure S47: Comparison of the ^1H NMR spectra of compounds **2a** and **2b** (600 MHz, $CDCl_3$)

I)

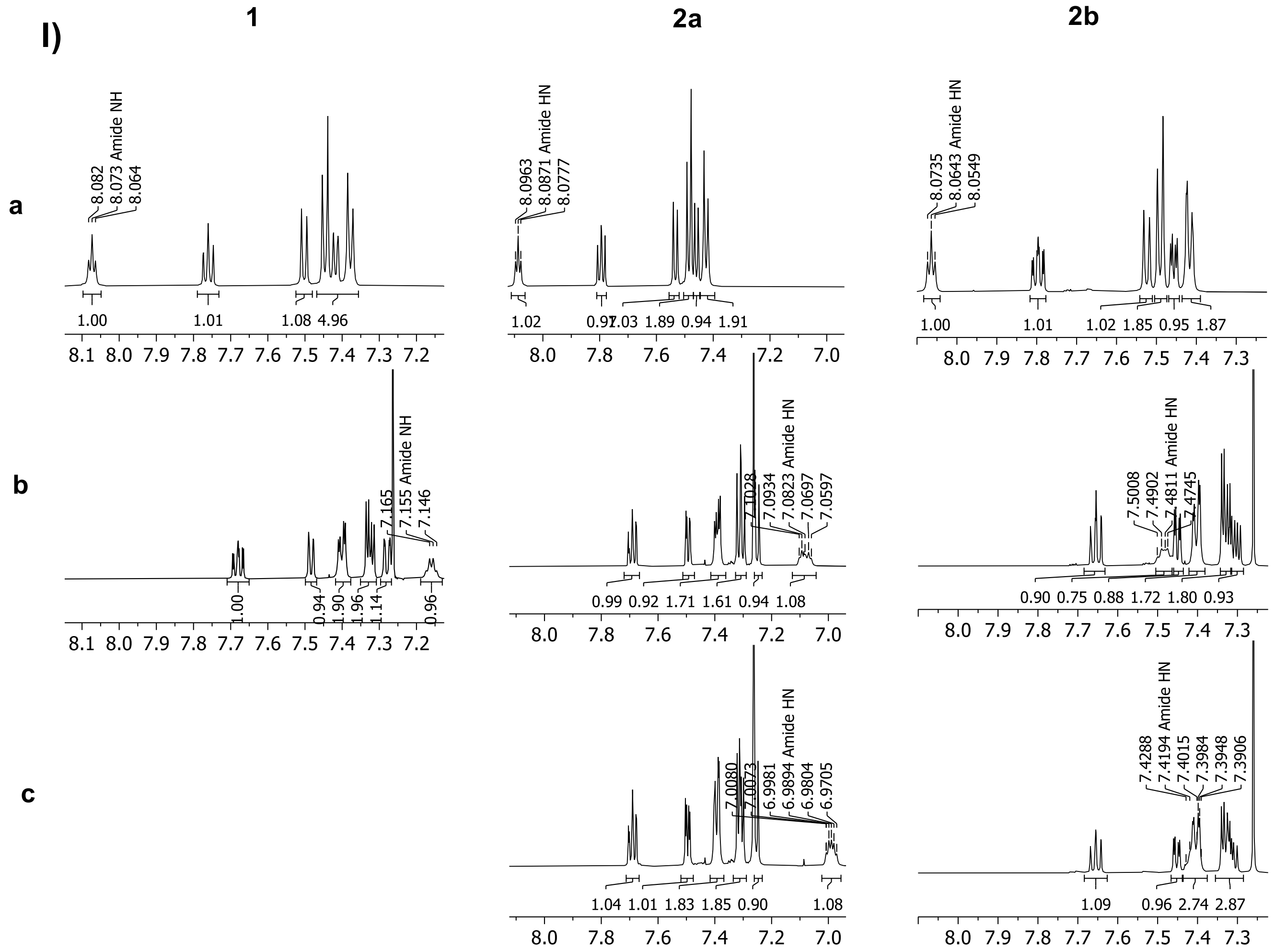

II)

	CDCl₃ (ppm)		DMSO-d_6 (ppm)	$\Delta\delta$ (δ_{DMSO} − δ_{CDCl_3})		A_{NMR} (= 0.0065 + 0.133$\Delta\delta$)[a]	
measurement	1	2		1	2	1	2
**1 (amide NH)**	8.07		7.16	0.91		0.13	
**2a (amide NH)**	6.99	7.08	8.09	1.1	1.0	0.15	0.14
**2b (amide NH)**	7.43	7.48	8.07	0.64	0.59	0.09	0.08

Figure S48: I) Partial ^{1}H NMR of compound **1** (left column), **2a** (middle column) and **2b** (right one) showing the chemical shift of amide NH a. (600 MHz, DMSO-d_6) b. and c. (600 MHz, $CDCl_3$). **II)** Tabulated chemical shift of amide NH in both compounds in $CDCl_3$ and DMSO-d_6 and calculated A_{NMR} values.

a. Reference: J. Org. Chem. 2006, 71, 9, 3389–339

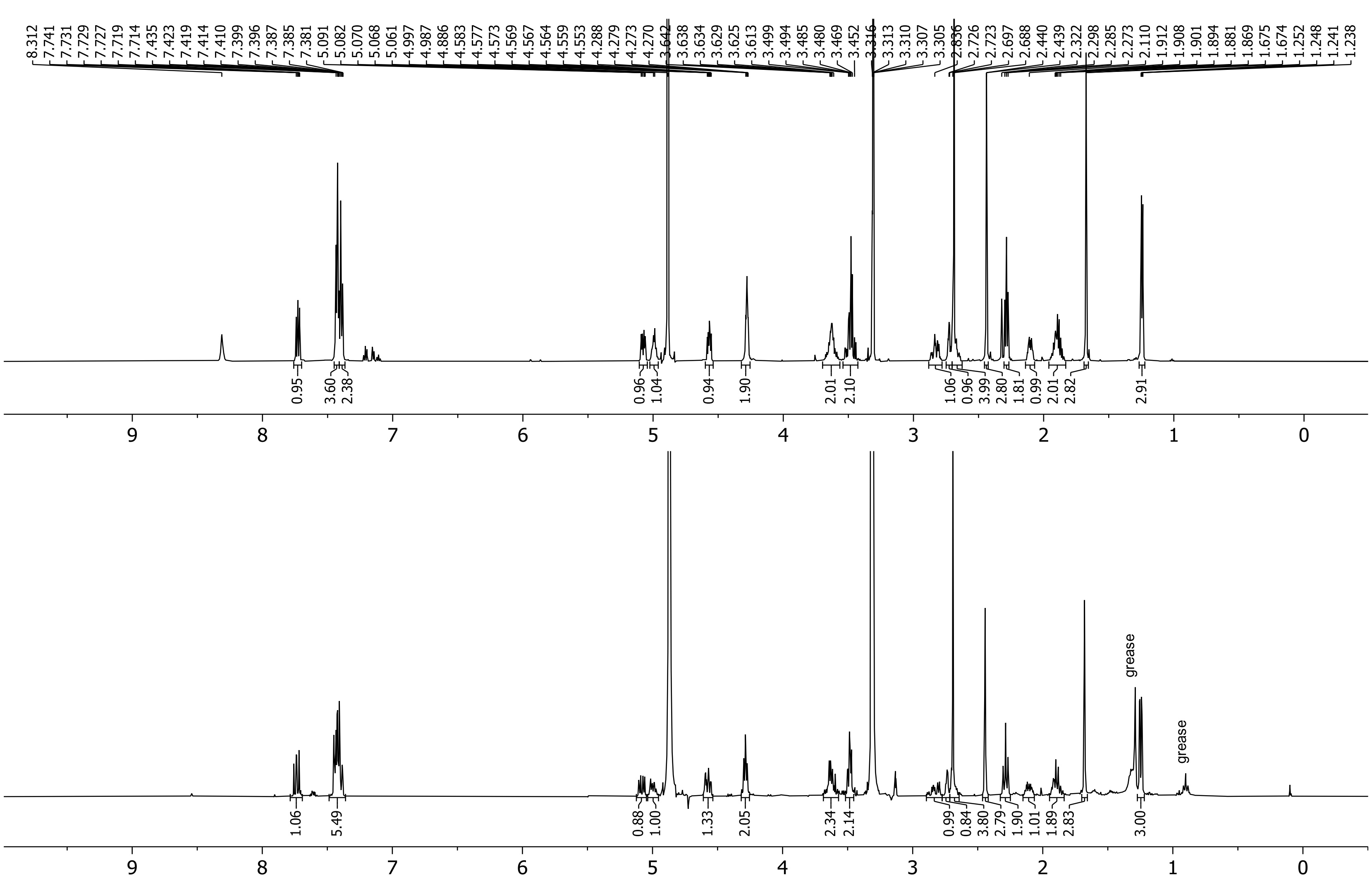

Figure S49: Comparison of the ^{1}H NMR spectra of compound **2a**: (top) isolated from the diastereomeric mixture (600 MHz, MeOH-d_4); (bottom) obtained via chiral pool synthesis starting from an enantiomerically pure starting material (500 MHz, MeOH-d_4).

HPLC charts

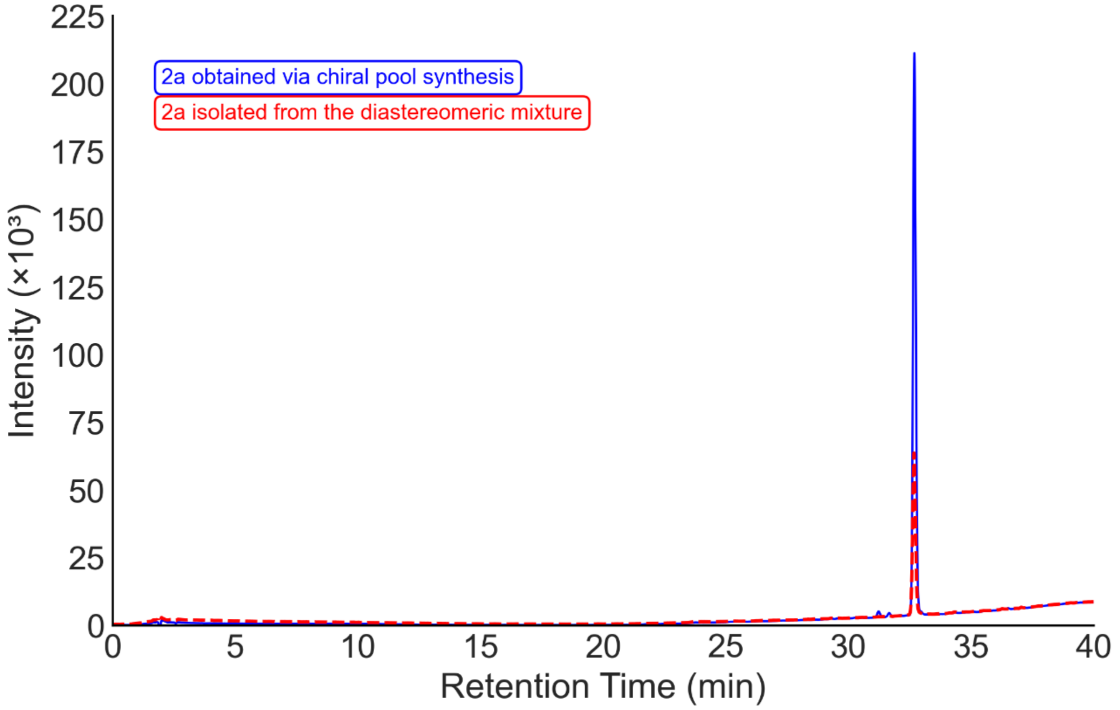

Figure S50: HPLC chromatogram of compound **2a**: the **red dashed line** represents the sample isolated from the diastereomeric mixture, while the **blue line** corresponds to the sample obtained via chiral pool synthesis using an enantiomerically pure starting material.

Western blot images used for Figure 2

Cropped images

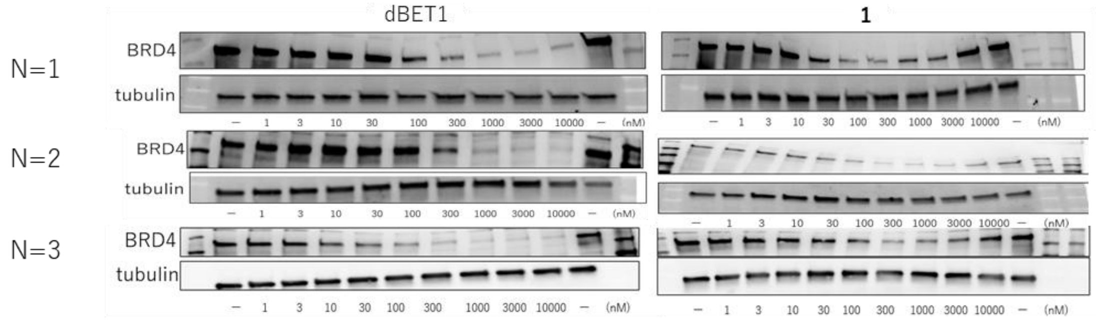

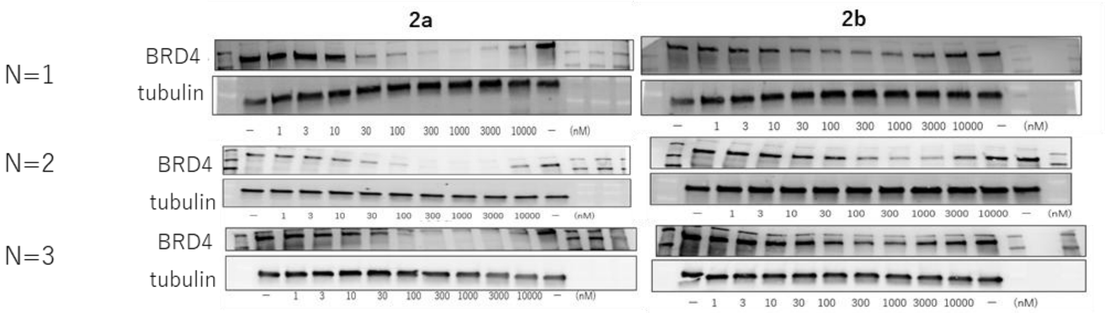

Uncropped images

N=1

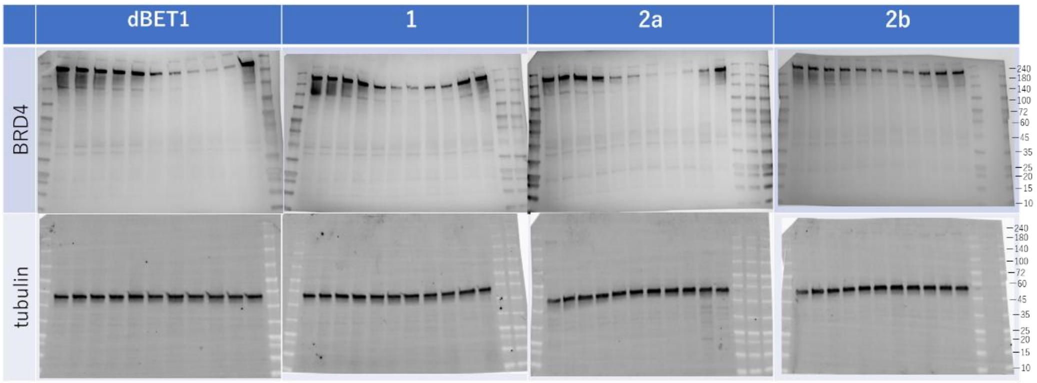

N=2

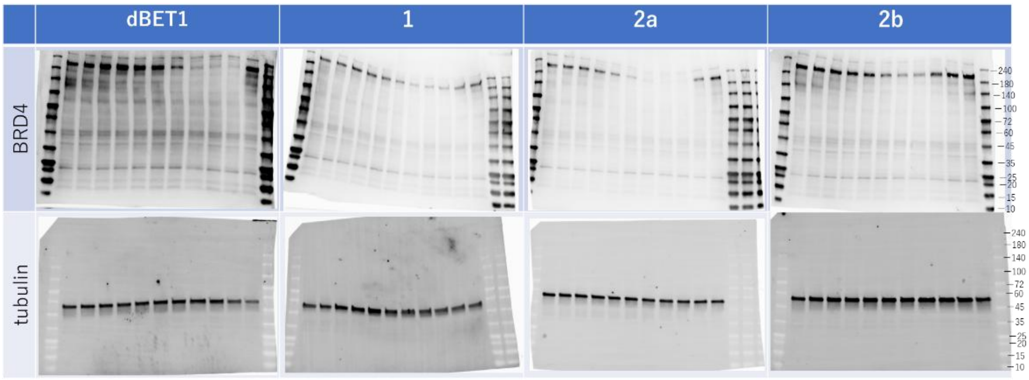

N=3

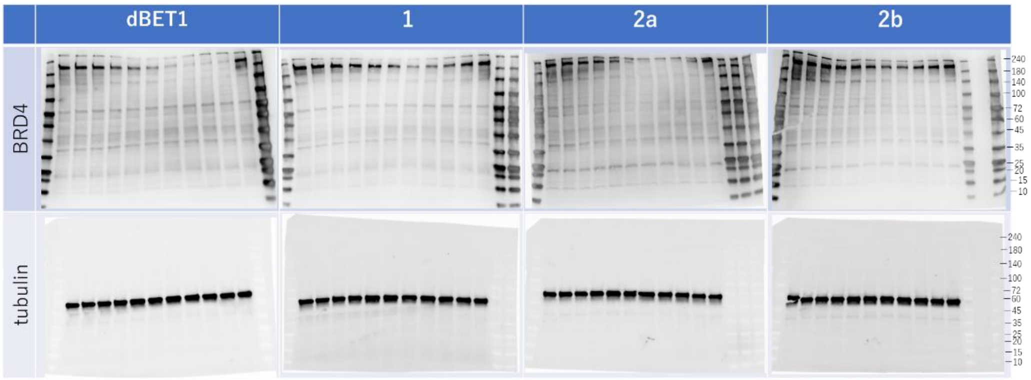
dBET1
1
2a
2b
BRD4
tubulin
240
180
140
100
72
60
45
35
25
20
15
10
